# Synthesis and Persistence Length Study of Defect-Free and Non-Aggregated Conjugated Ladder Polymers

**DOI:** 10.1021/jacsau.5c01162

**Published:** 2025-12-04

**Authors:** James Shao-Jiun Yang, Vijaya Sundar Jeyaraj, Guorong Ma, Daniel Doria, Xiaodan Gu, Daniel Tabor, Lei Fang

**Affiliations:** † Department of Chemistry, 14736 Texas A&M University, College Station, Texas 77843, United States; ‡ School of Polymer Science and Engineering, Center for Optoelectronic Materials and Devices, 5104 The University of Southern Mississippi, Hattiesburg, Mississippi 39406, United States; § Center for Functional Organic Materials, 621074 Yongjiang Laboratory, Ningbo, Zhejiang 315201, China

**Keywords:** conjugated ladder polymers, polymer synthesis, polymer conformation, persistence length, semiflexibility, neutron scattering, machine learning

## Abstract

A fundamental understanding of the solution properties of conjugated ladder polymers (CLPs) is essential for advancing their design, synthesis, and solution processing toward high-performance optoelectronic applications. Nevertheless, elucidating the solution conformation of CLPs remains a significant challenge in the field of polymer physics, owing to the difficulty of synthesizing defect-free samples, their intrinsically low solubility that results in weak signals and limited analytical accuracy, the pronounced tendency of CLPs to aggregate even when dissolved, and the absence of reliable theoretical models. Here, these fundamental challenges are addressed by the synthesis, neutron scattering measurements, and computational simulations of two model CLPs, **LP1** and **LP2**. Owing to their bulky three-dimensional side chains, **LP1** and **LP2** exhibit a non-aggregated character and high dispersibility as single polymer chains. Small-angle neutron scattering revealed unexpectedly low persistence lengths (*L*_p_) of 3–5 nm. The *L*_p_ being similar to those of non-ladder conjugated polymers such as P3HT indicates the long-range conformational semiflexibility of CLPs despite them possessing a ladder-type constitution. Machine learning-based molecular dynamics simulations further showed that the semiflexibility of these CLP chains mainly results from the pronounced out-of-plane deformations, which is synergistically influenced by the steric congestion of the side chains. Overall, a comprehensive experimental and computational approach demonstrates that CLPs, despite their fused-ring polyaromatic backbones, are best described as ribbon-like semiflexible chains, in contrast to the common belief that they are rigid-rod polymers.

## Graphical abstract

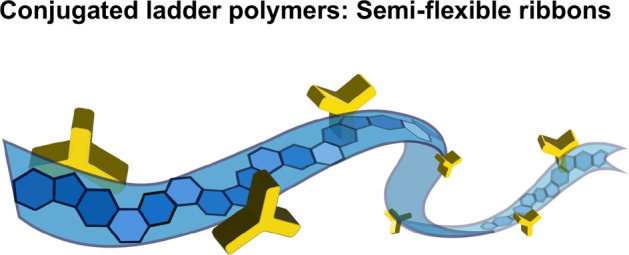

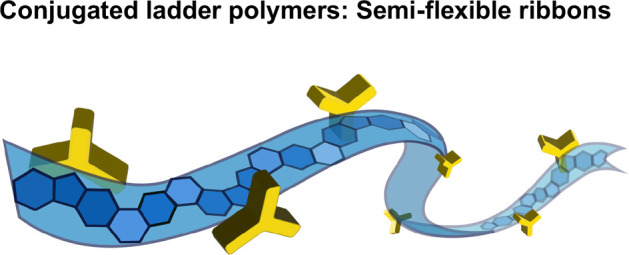

## 1 Introduction

Conjugated ladder polymers (CLPs) represent a unique class of macromolecules featuring multiple-stranded backbones with an uninterrupted sequence of fused, π-conjugated rings.^1–4^ This unique constitution creates the local rigidity and coplanarity of CLPs, resulting in low reorganization energy for efficient charge transport.^5^ In addition, the exceptional durability of many CLPs renders them promising electronic materials capable of operation under harsh conditions.^1,3,6^ Despite the growing interest in their electronic applications, the intrinsic polymer physics of CLPs, such as backbone conformation and rigidity, remains largely unexplored. In contrast, extensive studies on these properties of single-stranded conjugated polymers have provided valuable insights on advancing their electronic performances over the past few decades.^7–13^ In this context, a clear understanding of the conformation and chain rigidity of CLPs is critical to guiding the rational design, synthesis, and processing of these materials, as well as to realizing their long-desired practical applications.

The rigidity of conjugated polymers is often characterized by their persistence length (*L*_p_) (Figure 1a).^9^ *L*_p_ is the length over which the direction of a polymer chain’s tangent vector becomes decorrelated due to bending. For instance, poly­(3-hexylthiophene) (P3HT) has an *L*_p_ of ca. 3 nm at room temperature, representing a typical semiflexible polymer.^14,15^ The increase of *L*_p_ to 5 nm for some stepladder conjugated polymers implies the rigidifying effect to backbones caused by a ladder-type fused-ring constitution.^16^ A further increase of *L*_p_ to 10–20 nm was reported for many donor–acceptor step-ladder polymers, although this backbone stiffening might be a result of the quinoidal resonance structure and steric effect of the often large side chains.^8,11,17^ Still, such a trend implies that CLPs might have higher *L*_p_ compared to their non-ladder counterparts due to the double-stranded constitution. Indeed, CLPs are locally rigid at length scales shorter than 2 nm at the time scale of femtoseconds to nanoseconds, evidenced by distinguished vibronic progression observed in their optical spectra.^18^ Although a high *L*_p_ (45 nm) has been reported for double-stranded DNA,^19^ its stiffness is mainly a combined result of a charged backbone, hydrogen bonding interactions, and interactions between base pairs. Similarly, poly­(benz­imid­azo­benzo­phen­anthro­line) (BBL),^20^ one of the most representative CLPs, exhibited a measured *L*_p_ of 153 nm when dissolved in methanesulfonic acid. Such an ultrahigh *L*_p_ for BBL could originate from aggregation in solution or charge–charge repulsion of the protonated backbone. By contrast, Ballauff and co-workers reported the *L*_p_ of ladder-type poly­(*para*-phenylene) (LPPP) as 6.5 nm. It is unclear whether the significantly shorter *L*_p_ is a result of uncyclized defects on the backbone or the true reflection of the intrinsic semiflexibility of LPPP.^21^ Taken together, the *L*_p_ of CLPs in common organic solvents can be greatly influenced by a variety of factors, and the true value has not yet been determined in a convincing manner.

**Figure 1:**
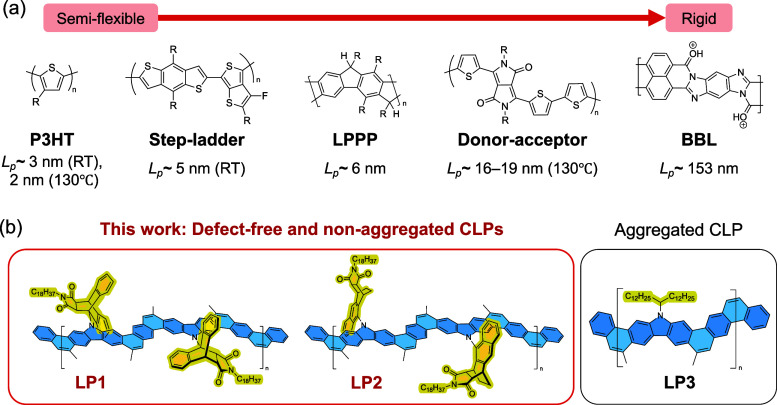
(a) Persistence length (*L*_p_) of common single-stranded conjugated polymers and representative CLPs.^11,15–17,20,21^ Temperature was not specified for the *L*_p_ of LPPP and BBL. (b) Defect-free, strained, and non-aggregated CLPs **LP1** and **LP2** studied in this work and previously reported **LP3** ^41^ that forms robust aggregates in solution.

The main backbone building blocks for CLPs are units resembling small-molecule polycyclic aromatic hydrocarbons and fused-ring carbon rich materials. Although these molecular species are often considered “rigid”, certain polycyclic aromatic molecules^22–24^ and macromolecules^25,26^ can a adopt non-coplanar conformation under certain conditions, showing a significant degree of flexibility while retaining their aromaticity.^27,28^ For example, twisted acenes^22^ can be synthesized through tethering^29–32^ or introducing steric hindrance.^33^ Various ladder-type carbon nanohoops,^34–36^ curved nanographenes,^37^ and π-conjugated materials such as fullerene, carbon nanotubes, as well as wrinkles and crumbles of graphene nanosheets^25,26^ have been reported and demonstrated significant distortion from the enthalpically favored coplanar conformation. The finding suggests that macromolecules composed of sp^2^-carbons could still adopt a semiflexible ribbon-like conformation. For instance, Narita and co-workers reported a non-coplanar graphene nanoribbon featuring embedded helicene moieties.^38^ Lupton and co-workers showed the semiflexibility of CLPs through optical spectroscopy on both ensemble and single-chain levels.^39^ Qin and co-workers reported a ribbon-like model to describe the conformation of CLPs computationally.^40^ Together, these precedent studies demonstrate that CLPs might not behave as rigid rods with large *L*_p_. Their semiflexibility could be achieved through various bending and twisting modes in the direction perpendicular to the double-stranded backbones. Nonetheless, a conclusive study on the intrinsic conformation of CLPs is still lacking. It remains unclear whether CLPs behave as semiflexible chains, like P3HT (*L*_p_ < 5 nm), or adopt a more rigid conformation similar to donor–acceptor stepladder polymers (*L*_p_ > 15 nm), and this discrepancy has yet to be fully resolved.

Assessing the intrinsic conformation of CLPs confronts several fundamental challenges, including synthesizing CLPs with no structural defects, preventing their ubiquitous aggregation,^41^ ensuring their solubility for true solution-phase characterization, and accurate simulations. In this work, we report our effort in investigating the rigidity of CLPs (Figure 1b), through a combined effort of the synthesis of rationally designed model polymers, small-angle neutron scattering (SANS), and machine learning-based molecular dynamics simulations. Our finding reveals the ribbon-like semiflexible conformation of CLPs as a result of out-of-ladder plane deformation despite their double-stranded ladder-type constitution.

## 2 Results and Discussion

To investigate the intrinsic chain conformation of CLPs at the single-chain level, model CLPs that are (i) non-aggregated and (ii) free of structural defects are required. Such defect-free CLPs can be synthesized via ring-closing metathesis (RCM),^42–44^ owing to the thermodynamic-driven and self-correcting nature of the RCM process. Nevertheless, such defect-free CLPs, such as **LP3**, form highly robust, nanoscale aggregates in solution, despite appearing visually soluble.^41^ Complicating matters further, the aggregation process is entropically favorable, such that simply increasing the temperature does not induce dissociation. As a result, direct investigation of the individual chain conformation of **LP3** is hindered. Moreover, the high propensity for dynamic aggregation among many conjugated polymers may lead to an overestimation of their reported *L*_p_, on account of inadequately accounting for aggregation effects, particularly for measurements performed without heating.

To address the ubiquitous issue of solution-phase aggregation of common CLPs, we employed the strategy of installing three-dimensional bulky anthracene-maleimide Diels–Alder adduct (AMA) as the side chain onto CLPs, inspired by works reported by Mai, Feng, Bogani, and co-workers.^45–47^ In this context, AMA-decorated defect-free CLPs **LP1** and **LP2** were designed and synthesized. These two compounds feature 9,10- and 1,4-AMA side chains, respectively. The synthesis started with the Diels–Alder reaction between anthracene-functionalized carbazole derivative **1** with an alkyl-functionalized maleimide in reflux *o*-xylene (Scheme 1). The reaction was unexpectedly sluggish in contrast to the fast and high yielding reaction on unhindered anthracene substrates. AlCl_3_ had to be employed to facilitate the reaction, surprisingly affording an unanticipated 1,4-AMA adduct **3** as the major product, while the anticipated 9,10-AMA adduct **2** was isolated only as a minor product. Although 9,10-addition of anthracene is generally more favorable, the regioselectivity could be altered toward 1,4-addition by properly adjusting its steric-electronic properties.^48–50^ This result suggests that the AMA moieties and the carbazole unit impose a high steric effect mutually to each other, both in **2** and **3**. From **2** or **3**, a series of Friedel–Crafts acylation and Wittig reaction was carried out to furnish the synthesis of monomers **4** and **5** (Scheme 1). It is noteworthy that the free olefin function of **3** was hydrogenated to avoid potential ring-opening metathesis during the later RCM ladderization step. The two derivatives stayed as an inseparable mixture until the last step, where compounds **4** and **5** were isolated and fully characterized.

**Scheme 1:**
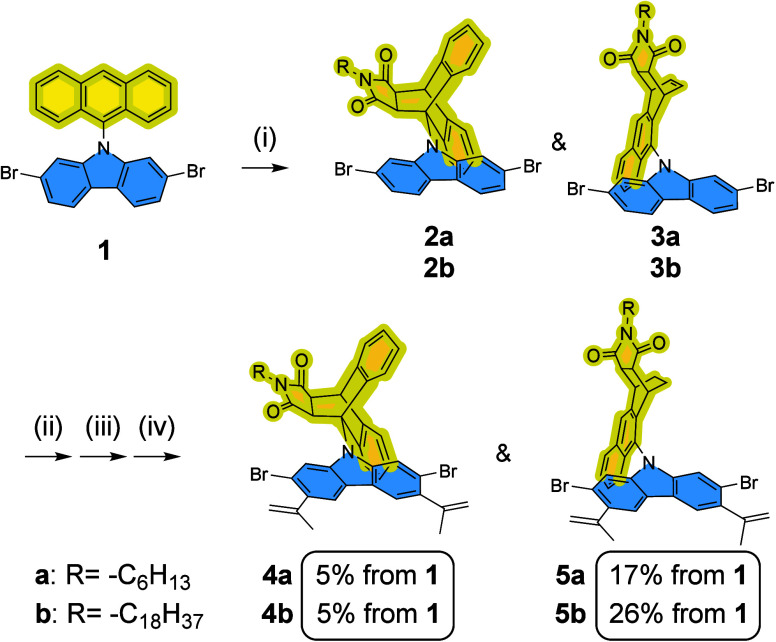
Synthesis of carbazole-derived monomers with 9,10-AMA (**4a** and **4b**) and 1,4-AMA side chains (**5a** and **5b**)­[Fn sch1-fn1]

a
**2** and **3** are inseparable, and the mixture was used for the following three steps to yield isolable monomers **4** and **5**. Reaction conditions: (i) alkyl maleimide, AlCl_3_, DCM, 0 °C to RT, 4 h; (ii) N_2_H_4_, H_2_O_2_, THF/EtOH/H_2_O, RT, 18 h; (iii) AcCl, AlCl_3_, 1,2-DCE, 45 °C, 1 h; and (iv) Ph_3_PCH_3_Br, KO^t^Bu, THF, RT, 1 h.

CLPs **LP1** and **LP2** and their corresponding small-molecule models **7** and **9** were synthesized through Suzuki coupling followed by RCM (Scheme 2). After Suzuki coupling with 2-vinylphenyl pinacol boronic ester, the resulting intermediates **6** and **8** demonstrate significant broadening and unusual splitting of the ^1^H NMR signals, which are indicative of hindered rotation of the end-capped styrene. For **6**, one of the isopropenyl groups and one of the aryl protons on carbazole exhibit additional splitting in its ^1^H NMR spectrum at room temperature, which coalesces when the temperature is elevated to 60 °C (Figure S51). In contrast, this effect is less pronounced for **8** and is even absent for carbazole with an alkyl side chain,^43^ showcasing the significant steric encumbrance exerted by 9,10-AMA. Subsequently, RCM of **6** and **8** affords the ladder-type products **7** and **9**, respectively, with high isolated yield. Compounds **7** and **9** were fully characterized through various NMR techniques, including ^1^H, ^13^C, ^1^H–^1^H COSY, ^1^H–^1^H NOESY, and ^1^H–^13^C HSQC spectra (Figures S31–S35).

**Scheme 2:**
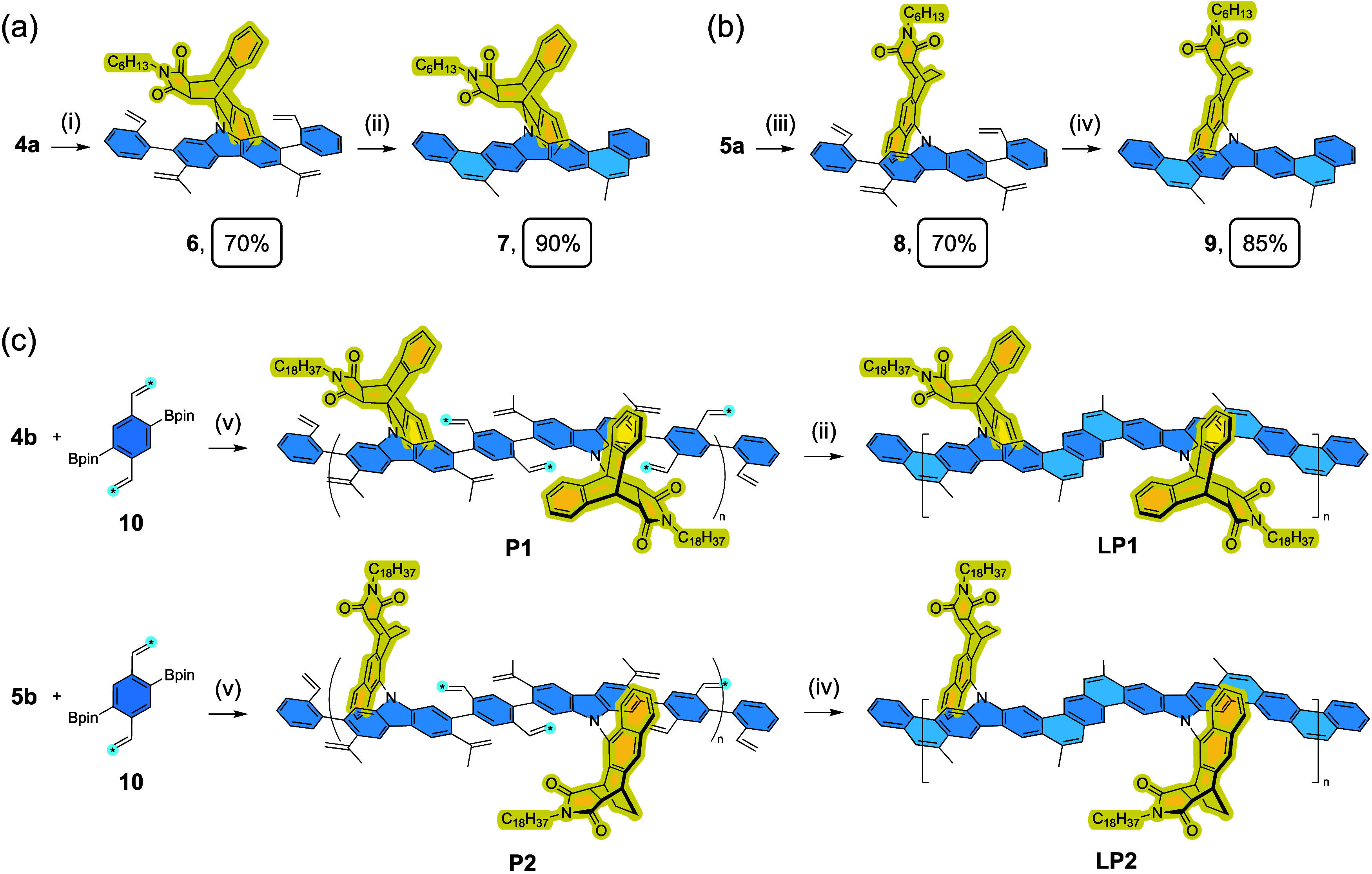
Synthesis of small-molecule models (a) **7** and (b) **9** through Suzuki coupling followed by RCM and (c) Synthesis of defect-free and non-aggregated CLPs **LP1** and **LP2** through Suzuki polymerization followed by RCM^a^

a The asterisks highlighted in blue in **10**, **P1**, and **P2** denote ^13^C-enriched isotopes. Reaction conditions: (i) 2-vinylphenyl pinacol boronic ester, Pd­(PPh_3_)_4_, K_2_CO_3_, Aliquat 336, BHT, PhMe, H_2_O, reflux, 18 h; (ii) Grubbs II, BHT, PhMe, reflux, 72 h; (iii) 2-vinylphenyl pinacol boronic ester, Pd­(PPh_3_)_4_, K_2_CO_3_, Aliquat 336, BHT, PhMe, H_2_O, reflux, 4 h; (iv) Grubbs II, BHT, DCM, 70 °C (pressurized), 48 h; and (v) Pd­(PPh_3_)_4_, K_2_CO_3_, Aliquat 336, BHT, PhMe, H_2_O, reflux, 24 h, then end-capped with 2-bromostyrene and 2-vinylphenyl boronic acid.

With the established synthesis of these model compounds, polymerization was carried out for **4b** and **5b** with comonomer **10**, followed by RCM to afford CLPs **LP1** and **LP2**. The vinyl groups of **10** are enriched with ^13^C isotopes so that the progress of RCM can be determined by ^13^C NMR spectroscopy. Due to the high rotational barrier of the divinyl phenylene units of **P1**, as can be inferred from **6**, full conversion of RCM needs to be accomplished in reflux toluene to provide sufficient thermodynamic driving force. Under this condition, Grubbs second generation catalyst was added slowly via a syringe pump to prevent undesired decomposition of the catalyst. In contrast, the RCM of **P2** proceeded smoothly in pressurized DCM at 75 °C without the need for the slow addition of Grubbs catalyst. The chemical constitution of **LP1** and **LP2** was fully characterized (Figure 2 and Figures S38–S41). Despite peak broadening as a common feature of polymers, the ^1^H NMR spectra of **LP1** and **LP2** exhibit good agreement with those of model compounds **7** and **9**. The relatively low isolated yields for both CLPs are a result of fractionation and removal of low-molar-mass oligomers as part of the purification process. For future work, multiangle light scattering analysis of the sample using a laser wavelength not absorbed by the polymer samples would be helpful for determining the absolute molar mass of the samples.

**Figure 2:**
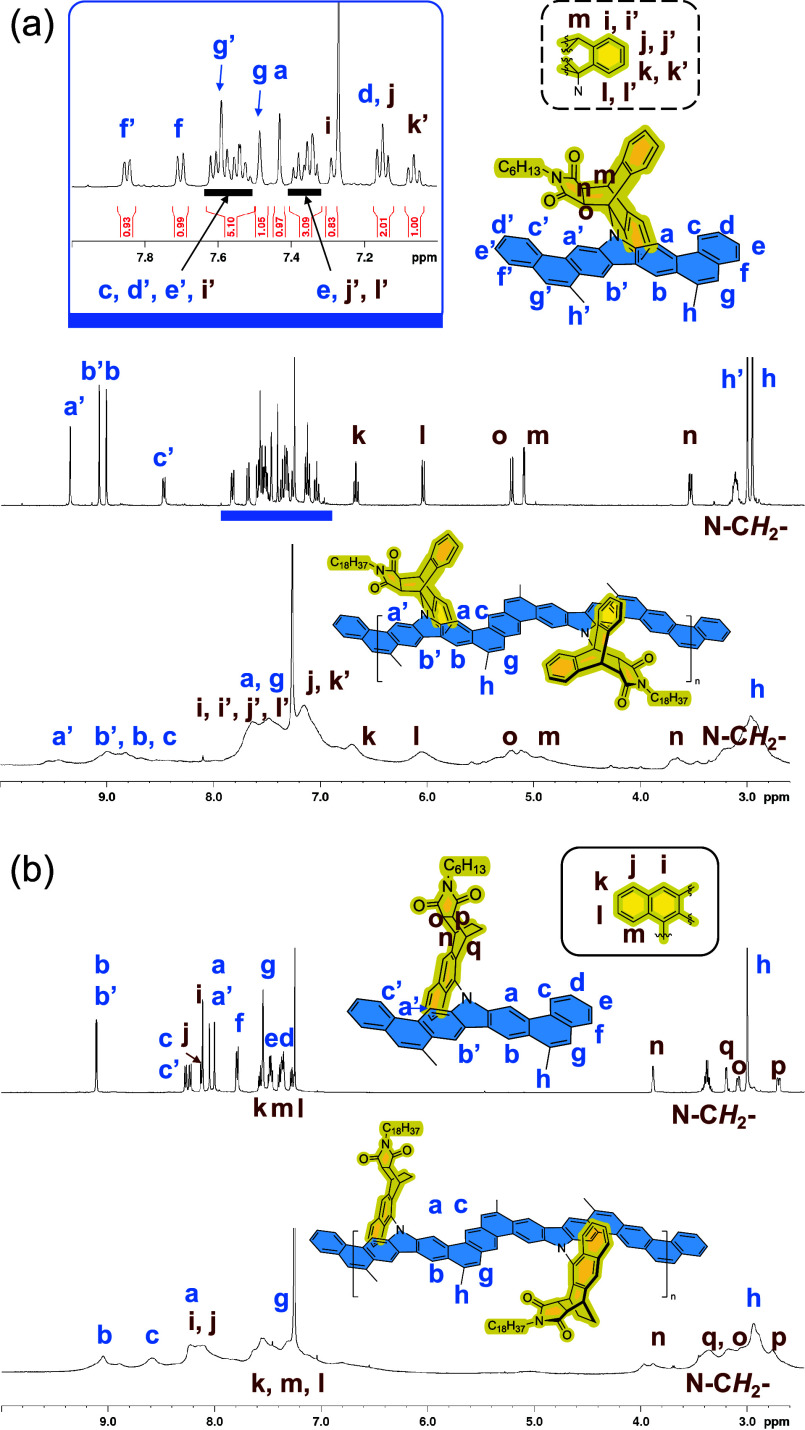
Partial ^1^H NMR of (a) **7** and **LP1** and (b) **9** and **LP2** in CDCl_3_.

The incorporation of the bulky AMA units onto the carbazole-derived CLPs results in significant steric encumbrance, which is essential for preventing the aggregation of CLPs that was observed on **LP3**. The steric congestion was corroborated by the unusual Diels–Alder reactivity of **1** as described above. The inactivity of the thermal Diels–Alder reaction of **1** and the predominant formation of atypical 1,4-AMA adduct **3** implied that the 9,10-adduct **2** is highly sterically congested. For **7**, such congestion results in a significant shielding effect on of the aromatic moiety of 9,10-AMA and carbazole observed on ^1^H NMR, leading to an upfield shift of proton *l* to 6.06 ppm and a remarkable difference of 1.94 ppm between the diastereotopic protons *a* and *a*′ (Figure 2a). Moreover, the C–N bonds connecting carbazole and the AMA moiety in both the 9,10- and 1,4-adducts are found to be restricted in free rotation, as evident by the formation of distinctive atropisomer products **11** and **12** that can be easily separated by chromatography upon monoacylation (Figure 3a). Specifically, H^1^ on the carbazole of **11**-α exhibits a shielded singlet, while H^8^ of **11**-β demonstrates a shielded doublet (^4^ *J*(H^8^–H^6^) = 1.3 Hz) (Figure S48). The nuclear Overhauser effect (NOE) provides further insight into the atropisomers of **12**-α and **12**-β. An NOE cross peak was observed between H^b^ on 1,4-AMA and the doublet H^8^ on carbazole for **12**-α, while for **12**-β, H^b^ demonstrates an NOE signal over the singlet H^1^ on carbazole (Figure S50). These atropisomers were found to be bench-stable for at least 2 years at room temperature, and no interconversion was observed at 100 °C as judged by variable-temperature NMR (Figure S49). The steric congestion further results in induced strain for the ladder-type backbones. The single-crystal X-ray structures of **7** and **9** not only confirm the desired structures unambiguously but also reveal distorted conformation along the out-of-plane direction (Figure 3b). From **13** to **9** and to **7**, increased non-coplanarity of ladder-type backbones was observed as the steric congestion enhanced. The strain of both **7** and **LP1** was further supported by their lower retro-Diels–Alder reaction temperature as compared to that of the unstrained AMA (Figures S53 and S54). UV–vis absorption and photoluminescence spectra of **LP1** and **LP2** reveal clear vibronic progression, suggesting rigidity in a short length scale of conjugation at the time scale of photoabsorption and emission. Furthermore, a blue shift was observed as more sterically congested side chains were incorporated, in agreement with the reduced effective conjugation as backbone strain and distortion increased (Figure 4a). The trend in blue shift was also observed for small-molecule models **9** and **7** (Figure S52), verifying that such an optical shift is not a direct result of polymer disaggregation. For **LP1**, the conjugated backbone accounts for 97.2% and 94.3% of the HOMO and LUMO compositions, respectively, as revealed by density functional theory (DFT) calculation, confirming that both orbitals remain delocalized across the π-conjugated core. In **LP2**, however, the HOMO remains predominantly on the conjugated ring system (99.7%), while the LUMO contribution from the backbone decreases dramatically to 13.2%, with the remaining density localized on the 1,4-AMA moieties (Supporting Information). The 1,4-AMA moieties of **LP2** perturb the LUMO through both electronic and steric effects: steric crowding around the C–N bond reduces conjugation of the backbone and side chain, while the electron-withdrawing nature of the side chain stabilizes its local LUMO level. Together, these effects result in an electronically decoupled side chain and a distinct backbone-to-substituent charge-transfer character.

**Figure 3:**
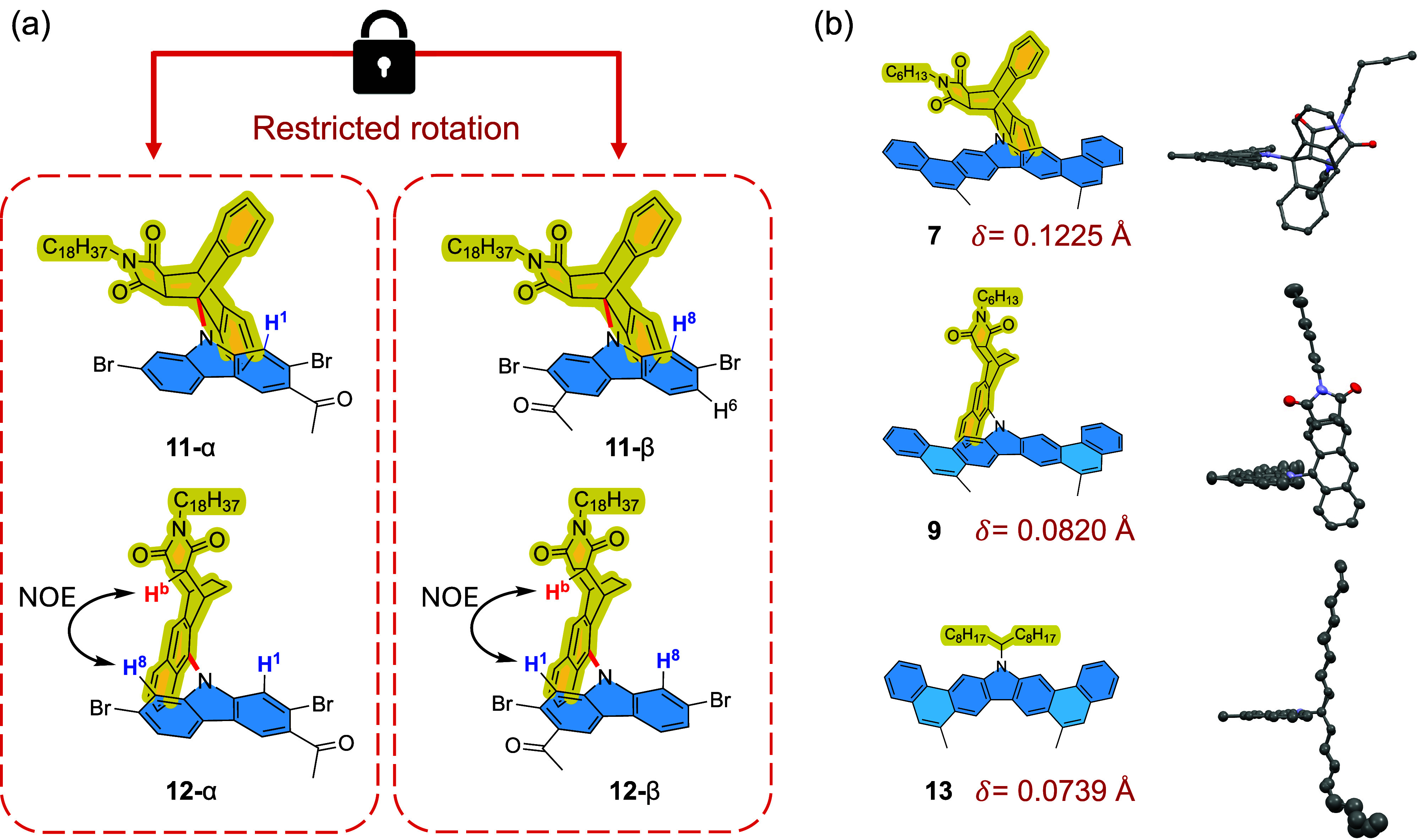
(a) Restricted rotation of C–N bond (highlighted in red) and the corresponding atropisomers formed during Friedel–Crafts monoacylation. **11**-α and **12**-α stay as an inseparable mixture, which is distinct from the other inseparable mixture consisting of their atropisomers **11**-β and **12**-β. (b) Single-crystal X-ray structures of **7**, **9**, and **13**.^43^ The thermal ellipsoids are scaled to the 50% probability level. Solvent molecules and hydrogen atoms are omitted for clarity. The non-coplanarity is determined by the root-mean-square displacement (δ) of backbone carbon atoms to their respective regression planes.

**Figure 4:**
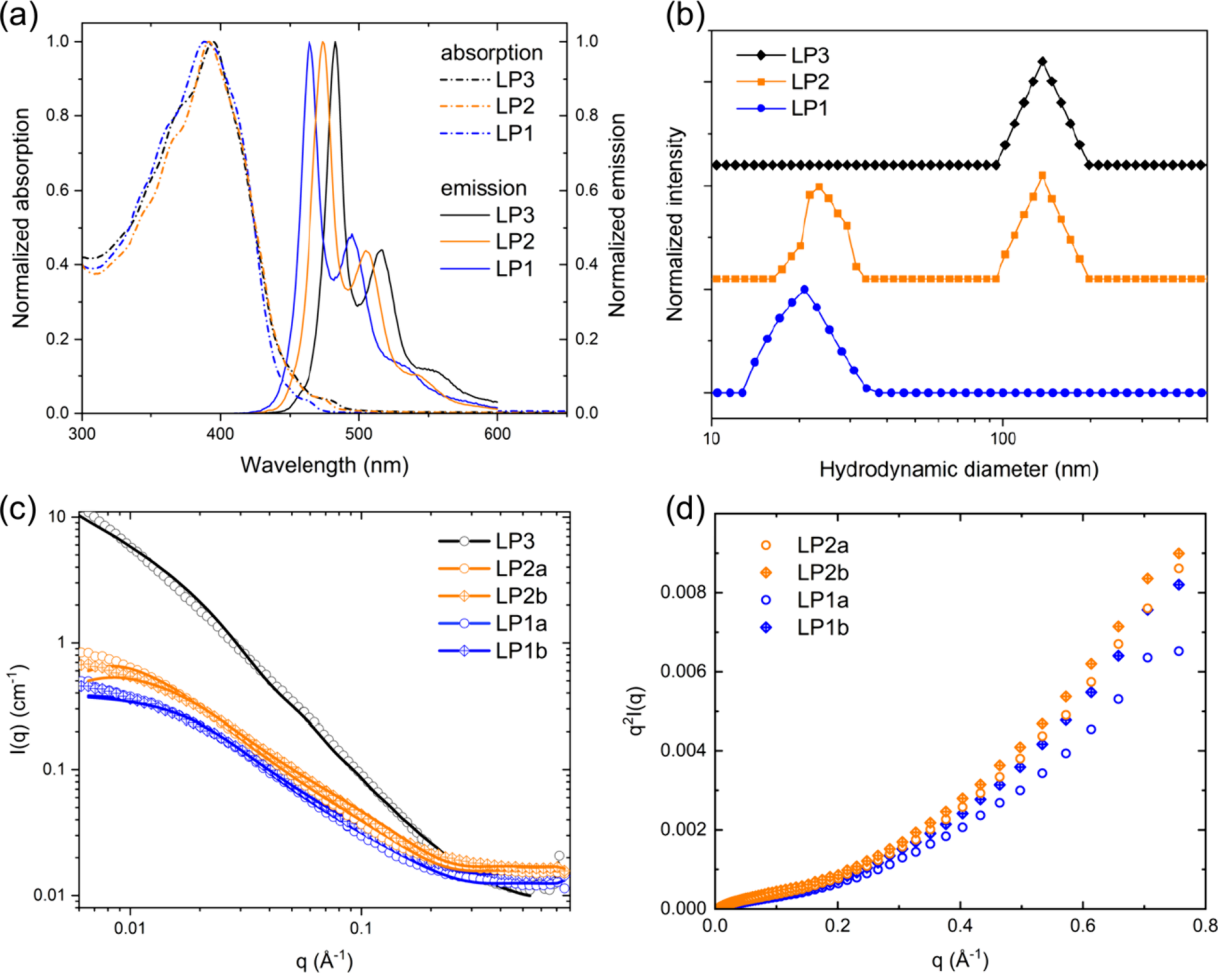
(a) UV–vis absorption and photoluminescence spectra of **LP1**, **LP2**, and **LP3**,^43^ all showing clear vibronic progression. A clear blue shift is observed from **LP3** to sterically congested **LP1**. (b) DLS profiles of **LP1** (10 mg/mL), **LP2** (10 mg/mL), and **LP3** (1 mg/mL)^41^ dissolved in chlorobenzene at room temperature. (c) SANS profiles and curve fitting of **LP1** (10 mg/mL) and **LP2** (10 mg/mL) dissolved in 1,2-dichlorobenzene-*d*_4_ at 130 °C and **LP3** (5 mg/mL)^41^ dissolved in chlorobenzene-*d*_5_ at 75 °C. A fractal number of nearly unity was found for both **LP1** and **LP2** under the experimental conditions, suggesting their single-chain features relative to the aggregate **LP3**. (d) Kratky plot of **LP1** and **LP2** showing a monotonic increase for *q* values above 0.2 Å^–1^ that signifies the contrast of local rigidity and long-range semiflexibility.

With the successful synthesis of **LP1** and **LP2** with sterically congested side chains, their solution-phase polymer physics were characterized by dynamic light scattering (DLS) (Figure 4b) and small-angle neutron scattering (SANS) (Figure 4c). Our previous report on branched alkyl chain-decorated **LP3** showed that the polymer forms dynamic aggregation at the scale of 100 nm in a broad range of temperatures according to DLS and SANS profiles.^41^ In sharp contrast, DLS of the chlorobenzene solution of **LP1** at room temperature shows the complete absence of aggregation and only the signal of non-aggregated polymer chains. For the chlorobenzene solution of **LP2** at room temperature, a bimodal distribution at ca. 20 and 100 nm was observed, which was attributed to the coexistence of aggregates and non-aggregated polymer chains (Figure S58). These results suggest that the 9,10-AMA side chain exerts a pronounced effect on preventing the aggregation of CLPs in solution. SANS profiles of both **LP1** and **LP2** in 1,2-dichlorobenzene-*d*_4_ at 130 °C reveal a fractal number close to unity (Table 1), indicating the presence of one-dimensional non-aggregated CLP chains as the dominant species.

**Table 1:** Fitting Parameters of the SANS Profiles for **LP1** and **LP2** Using the Flexible Cylinder Model^a^

Sample	*M*_n_ ^a^	*M*_w_ ^a^	*Đ*	Fractal number	Contour length (nm)	Kuhn length (nm)	*L*_p_ (nm)	Radius (nm)
**LP1**a	16.7	23.3	1.40	0.82	56.4 ± 0.01	5.6 ± 0.10	2.8 ± 0.05	1.0 ± 0.01
**LP1**b	10.0	15.8	1.58	0.79	45.7 ± 0.59	7.2 ± 0.21	3.6 ± 0.10	1.1 ± 0.01
**LP2**a	16.2	21.1	1.30	0.95	81.5 ± 0.65	7.7 ± 0.13	3.8 ± 0.06	1.2 ± 0.01
**LP2**b	10.1	16.7	1.65	0.88	46.0 ± 0.37	10.6 ± 0.19	5.3 ± 0.10	1.2 ± 0.01

a Both polymers were fractionated by recycling size exclusion chromatography to yield two fractions with higher *M*_n_ (denoted as “a”) and lower *M*_n_ (denoted as “b”) fractions.

b *M*_n_ and *M*_w_ (in kg/mol) were measured by analytical size exclusion chromatography.

**LP1** and **LP2** were fractionated by recycling size exclusion chromatography to afford two fractions for each, denoted as “a” and “b” for high-molar-mass and low-molar-mass fractions, respectively (Table 1, Figure S57). SANS measurements were conducted on these samples to probe their single-chain conformation. The results exhibited the semiflexibility on these samples as shown by an *L*_p_ of 2.8–3.6 nm for **LP1** and 3.8–5.3 nm for **LP2** (Table 1). Despite the weak dependence of the *L*_p_ on the molar mass, **LP1** is overall more flexible than **LP2**. The measured *L*_p_ values of all samples are on the same order as those of LPPP and P3HT while demonstrating significant difference with those of reported donor–acceptor conjugated polymers (Figure 1a). Based on our SANS analysis and the evidence from small-molecule models, **LP1** and **LP2** are classified as ribbon-like semiflexible polymers despite having a ladder-type constitution. From the Kratky plot, *I*(*q*)*q ^2^ exhibits a clear monotonic increase for *q* values above 0.2 Å^–1^, aligning well with our fitted persistence length of ca. 3 nm (Figure 4d). This result indicates that both CLPs form fully dissolved chains that behave like rigid rods at length scales below 3 nm, suggesting their local rigidity. Beyond this length scale, the CLP chains start to exhibit semiflexibility, presumably due to out-of-ladder-plane bending, behaving as semiflexible ribbons.

To better understand the semiflexible nature of **LP1** and **LP2**, molecular dynamics simulations were conducted across various chain lengths of oligomeric models of these CLPs and on P3HT oligomers as a reference of non-ladder conjugated polymers. The simulations were conducted based on the experimental conditions of SANS with three different underlying potentials: (1) OpenFF,^51^ (2) GAFF,^52^ and (3) the recently developed AIMNET2 ^53^ model, which is a machine learning-based potential that has been demonstrated with strong transferability. Though AIMNET is more computationally intensive than the OpenFF and GAFF potentials, the model is substantially faster than density functional theory (DFT) approaches and has been trained to match long-range corrected density functional theory results on a wide variety of organic molecules, including conjugated cycles. The observed values for *L*_p_ are directly related to the underlying force constants of ring distortion modes and thus are directly related to the choices made in formulating the empirical potential. Consistent with the observed *L*_p_ of 3–5 nm, the simulated chains (ranging from 5- to 11-mers) all exhibit bent conformations on the scale of a few nanometers, as clearly seen in the snapshots from the OpenFF simulation trajectories. It is worth emphasizing that **LP1** and **LP2** predominantly bend on the directions perpendicular to the backbone plane (Figure 5a,b,d,e), whereas P3HT displays both twisting and bending motions (Figure 5c,f). Notably, the overall semiflexibility and directionality of polymer chains can be clearly observed regardless of ladder-type constitution.

**Figure 5:**
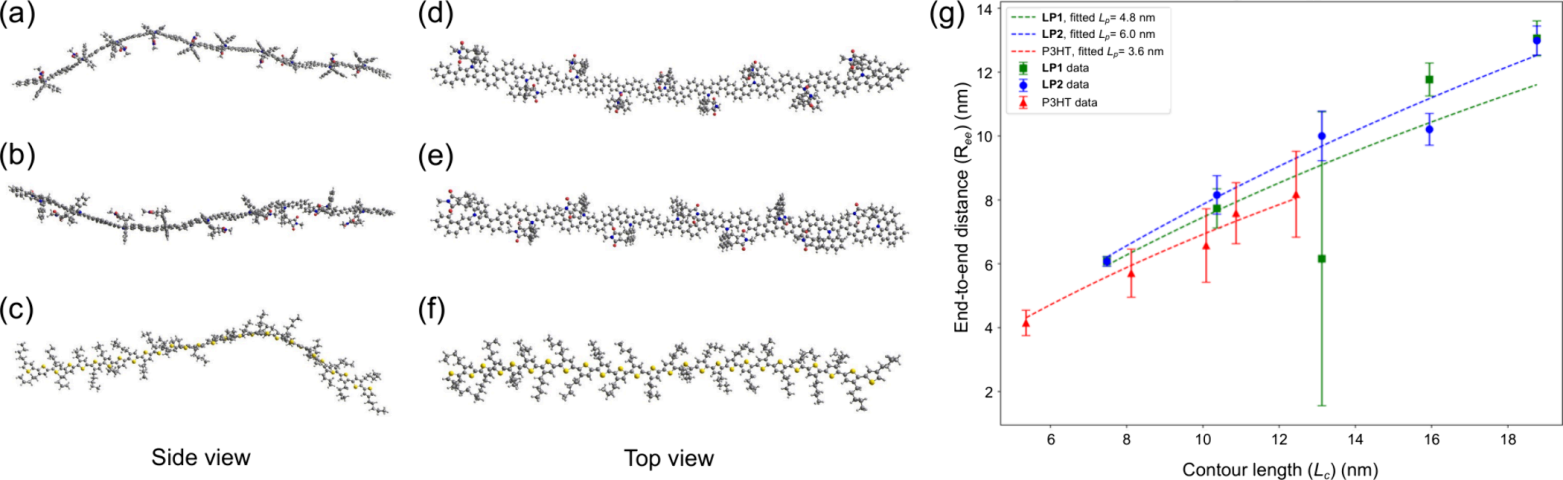
Snapshots from the side and top view of 9-mer of (a, d) **LP1**, (b, e) **LP2**, and (c, f) P3HT from OpenFF simulation trajectories. **LP1** and **LP2** exhibit a semiflexible ribbon-like conformation with out-of-plane bending. (g) *R*_ee_ and *L*_c_ with the worm-like chain fit using the AIMNET2 force field.

The local rigidity/flexibility of **LP1**, **LP2**, and P3HT was quantified by analyzing three consecutive inter-ring angle deviations: in-plane bending (θ_ipb_), out-of-plane bending (θ_oop_), and twist (θ_twist_) (Supporting Information). The averaged angular deviations from equilibrium geometries across all polymer lengths and types were evaluated using trajectories from the three force fields (Figure S61). Even though the average deviations from equilibrium angles are nearly zero, standard deviations offer insights into the bending modes of these polymers. Notably, θ_ipb_ and θ_oop_ deviations are only modest for all three polymers, while θ_twist_ deviation demonstrates substantially greater values for P3HT than for **LP1** and **LP2** for all lengths of oligomers under all force fields. These results indicate that torsional flexibility, as captured by θ_twist_, constitutes the primary difference of the local conformational variation of these polymers. The increased torsional deviation observed in P3HT underscores its locally twisted backbone, contrasting sharply with the local rigidity of **LP1** and **LP2** imparted by their ladder-type constitution. It should be noted that the local inter-ring twisting should be distinguished from the large-scale conformational semiflexibility and *L*_p_ of polymer chains.

To further understand the chain flexibility at a large length scale, the calculated end-to-end distance (*R*_ee_) was plotted against the contour length (*L*_c_) and fitted using the worm-like chain model (Figure 5g and Supporting Information). A linear correlation between *R*_ee_ and *L*_c_ and a consistent *R*_ee_/*L*_c_ ratio for the three polymers were established for all methods, implying their comparable conformational semiflexibility regardless of ladder-type constitution. A similar linear correlation between *R*_ee_ and *L*_c_ for P3HT was also reported in the literature.^54^ However, OpenFF and GAFF results (Figure S62a,b) found that the *R*_ee_/*L*_c_ ratio is smaller for ladder polymers, indicating that **LP1** and **LP2** may be trapped in conformations with shorter *R*_ee_ partly due to the construction of the dihedral potentials of the force fields, which could potentially limit polymer flexibility. The fit obtained from AIMNET2 simulation data (Figure 5g) predicts higher polymer flexibility, providing an approximate *L*_p_ of 3.6 nm for P3HT that accurately echoes the experimental value (ca. 2.0 nm at 130 °C). The same force field also estimates *L*_p_ values of 4.8 and 6.0 nm for **LP1** and **LP2**, respectively, which are also in relatively good agreement with their experimental values (ca. 3.6 and 5.3 nm, respectively, at 130 °C). Overall, the AIMNET2-based simulations (when compared to the GAFF and OpenFF potentials) show a greater polymer flexibility, yielding *L*_p_ estimates closer to those obtained from SANS experiments. This suggests that the model captures non-additive electronic structure effects that are not found from standard empirical potential parameters. Although the AIMNET simulations are limited to a shorter 2 ns trajectory and ideally longer time scale results could be obtained, we find in these short-run trajectories qualitative comparisons that effectively illustrate flexibility differences among the three polymers and illustrate the relevant degrees of freedom that influence flexibility.

Through a comprehensive investigation using SANS and machine learning-based molecular dynamics simulations, non-aggregated defect-free CLPs are best described as ribbon-like polymers in solutions. From molecular dynamics simulations, significant backbone distortion predominantly from out-of-plane bending was observed for **LP1** and **LP2**, resulting in their conformational semiflexibility. The ladder-type constitution indeed restricts the in-plane bending and twisting. Such local rigidity is evident by the clear vibronic progression observed in the optical spectra of **LP1** and **LP2**, while P3HT exhibits featureless absorption and emission profiles. Although high *L*_p_ values (>15 nm) were reported for many donor–acceptor stepladder polymers, some of these data might be overestimated due to the presence of polymer aggregation at room temperature. Besides, the quinoidal resonance of donor–acceptor conjugated polymers also contributes to backbone stiffening through electron delocalization, while this effect is marginal for highly aromatic **LP1** and **LP2**. It was found that doping a solution of poly­(3-butylthiophene) to its quinoidal oxidation state results in a more than 10 times increment of *L*_p_,^55,56^ although the possibility of polymer aggregation upon doping cannot be eliminated. One should perform temperature dependent scattering and spectroscopy to confirm the chain aggregation is removed.^57^ As mentioned earlier, out-of-plane bending is ubiquitously observed among conjugated molecules solely composed of sp^2^-carbons. It was computationally found that even a boat-form benzene retains 80% of its maximum ring current, suggesting the low enthalpy penalty of distorted aromatics.^27^ In the case of CLPs, the backbone semiflexibility can greatly impart their entropy gain, while local aromaticity is preserved with minimized enthalpy loss. Such thermodynamics is believed to account for the semiflexibility of **LP1** and **LP2**, which is further reinforced by the steric congestion from side chains.^10,11^ Nonetheless, it is challenging to experimentally verify the single-chain semiflexibility of side chain-free CLPs due to their insolubility. Therefore, introducing bulky AMA side chains becomes necessary; however, these side chains may also distort the backbone through steric hindrance. Although it is difficult to deconvolute the intrinsic semiflexibility of the polycyclic aromatic backbone from the effects of sterically congested side chains, our observations indicate that the observed semiflexibility arises from the synergistic contribution of both factors. Since the distortion of CLPs is closely related to entropy gain, we expect a lower persistence length *L*_p_ at higher temperatures because elevated temperatures provide sufficient energy to overcome the thermodynamic barrier to distortion.

## 3 Conclusions

In summary, we report the synthesis of two defect-free and non-aggregated CLPs, **LP1** and **LP2**, enabled by thermodynamic-driven RCM. To prevent the strong aggregation of these CLPs in solutions, sterically congested AMA side chains were incorporated onto the polymer backbones. The pronounced steric effect from the AMA side chains resulted in several unique features, including the unusual regioselectivity of the Diels–Alder reaction, the formation of atropisomers, and the out-of-plane distortion of the ladder-type backbone observed from the X-ray structures of model molecules. Compared with the CLP analogue with branched alkyl side chains, the single-polymer-chain dispersibility of **LP1** and **LP2** in solution was successfully achieved and confirmed by both DLS and SANS. Furthermore, the SANS profiles of **LP1** and **LP2** revealed their ribbon-like semiflexible backbones with characteristic *L*_p_ in the range of 3–5 nm, which is on the same order as those of other non-ladder conjugated polymers such as P3HT. The experimental SANS data underscore the conformational semiflexibility of **LP1** and **LP2**, even though their backbones consist of double-stranded fused aromatic structures. Through molecular dynamics simulations, it was evident that both **LP1** and **LP2** possess trajectories similar to those of P3HT in terms of polymer bending, and all three polymers have similar a *R*_ee_/*L*_c_ ratio that indicates comparable long-range conformational semiflexibility. The semiflexibility of **LP1** and **LP2** is reasonably attributed to (1) the preclusion of polymer aggregation, (2) the lack of backbone stiffening caused by quinoidal resonance, and more importantly (3) the out-of-plane distortion of the backbone reinforced by their sterically congested AMA side chains. Overall, this work provides a comprehensive investigation from polymer synthesis to scattering experiments and computational analysis that highlights the ribbon-like semiflexibility of CLPs.

## Supplementary Material



## Data Availability

All trajectory files and analysis scripts are available on GitHub at https://github.com/vijayasundar3927/Conjugated_Ladder_Polymers_Data.

## Notes

Additional experimental data, including synthetic experimental details, NMR spectra, single-crystal X-ray diffraction data, photophysical properties, dynamic light scattering data, size exclusion chromatograms, small-angle neutron scattering details, and computational analysis (PDF)
