# Supplementary material for: Synthesis and Persistence Length Study of Defect-Free and Non-Aggregated Conjugated Ladder Polymers
Source: JACS Au. 2025 Dec 4;5(12):6210–9. doi: 10.1021/jacsau.5c01162 (PMC12728607; doi:10.1021/jacsau.5c01162)
Supplement: Supplementary file 1 [file au5c01162_si_001.pdf]

Supporting Information (SI)

Synthesis and Persistence Length Study of Defect-Free and Non-Aggregated Conjugated Ladder Polymers

James Shao-Jiun Yang[1], Vijaya Sundar Jeyaraj[1], Guorong Ma[2], Daniel Doria[1], Dr. Xiaodan Gu[2]*, Dr. Daniel Tabor[1]*, Dr. Lei Fang[3]*

[1] Department of Chemistry, Texas A&M University, College Station, TX 77843, USA

[2] School of Polymer Science and Engineering, Center for Optoelectronic Materials and Devices, The University of Southern Mississippi, Hattiesburg, MS 39406, USA

[3] Center for Functional Organic Materials, Yongjiang Laboratory, Zhejiang, China

E-mail: xiaodan.gu@usm.edu, daniel_tabor@tamu.edu, lei-fang@ylab.ac.cn

Table of Content

1. **General Information 3**
2. **Syntheses 5**
3. **NMR spectra 20**
4. **Single-Crystal X-ray Diffraction 43**
5. **Determination of Restricted Rotation 47**
6. **Determination of Hindered Rotation 52**
7. **Photophyisical Properties 53**
8. **Determination of Retro-Diels-Alder Reaction 54**
9. **Size Exclusion Chromatograms (SEC) 56**
10. **Dynamic Light Scattering (DLS) 57**
11. **Small-Angle Neutron Scattering (SANS) 59**
12. **Orbital analysis of LP1 and LP2 .... 60**
13. **Molecular Dynamics Simulation 63**

    ***Computational Details** 66*

    ***Analysis Methods** 69*

    ***Trajectory snapshots of polymers of different lengths** 72*
14. **References 90**

1. General Information

Starting materials and reagents were purchased from commercial suppliers and used as received without further purification unless otherwise mentioned. Anhydrous THF was prepared by distillation over sodium. Anhydrous 1,2-dichloroethane was prepared by distillation over CaH2.Other anhydrous solvents were dried using IT pure solvent system (PureSolv-MD-5) and used without further treatment. 2,5-dibromoterephthalaldehyde was synthesized by reported procedures[1]. 1-octadecyl-1*H*-pyrrole-2,5-dione and 1-hexyl-1*H*-pyrrole-2,5-dione were synthesized by reported procedures[2] using octadecylamine and hexylamine. Methyl-^{13}C-triphenylphosphonium iodide (99% ^{13}C isotope enriched) was synthesized according to the reported procedures[3] starting from ^{13}C-methyl iodide (99% ^{13}C isotope enriched). Analytical thin-layer chromatography (TLC) was performed on glass-supported silica gel 60-F254 (Sorbtech). Column chromatography was carried out by Biotage® Isolera™ Prime instrument or Yamazen Automated Flash Chromatography System with Universal Column Premium. UV-Vis absorption spectra were recorded by Shimadzu UV-2600, while photoluminescence spectra were measured on Horiba Fluoromax-4. ^{1}H, ^{13}C, ^{1}H-^{1}H COSY, ^{1}H-^{1}H NOESY, ^{1}H-^{13}C HSQC, and ^{1}H-^{13}C HMBC nuclear magnetic resonance (NMR) spectra were recorded on Bruker 400 MHz and 500 MHz spectrometer at specified temperature and processed by Bruker TopSpin 4.2.0. Chemical shifts are reported in ppm relative to the signals corresponding to the residual non-deuterated solvents (for ^{1}H NMR: $CDCl_3$ δ = 7.26 ppm, toluene-*d8* δ = 2.09 ppm (PhC$\underline{H}_3$); for ^{13}C NMR: $CDCl_3$ δ = 77.16 ppm). Atmospheric-pressure chemical ionization high-resolution mass spectrometry (APCI-HRMS) experiments were performed using a Thermo Scientific Q-Exactive Focus operated in both positive and negative ion mode. Size exclusion chromatography (SEC) was performed on TOSOH

EcoSEC (HLC-8320GPC) in THF solution at 40 ℃, with the molar masses calculated by a calibration curve based on polystyrene standards, equipped with TSKgel SuperHM-M and TSKgel SuperH-RC. Preparative SEC was performed in chloroform solution at room temperature using JAI recycling preparative HPLC (LC-92XXII NEXT SERIES). X-ray single crystal diffraction measurements were made on a BRUKER APEX 2 X-ray (three-circle) diffractometer. The X-ray radiation employed was generated from a Mo sealed X-ray tube (K = 0.70173Å with a potential of 40 kV and a current of 40 mA) fitted with a graphite monochromator in the parallel mode (175 mm collimator with 0.5 mm pinholes). Differential scanning calorimetry (DSC) was measured on TA Q20 with a heating rate of 10 ºC/min from 40 to 350 ºC. Dynamic light scattering (DLS) was performed using a Brookhaven BI-200SM research goniometer with a BI-APD avalanche photodiode detector and a 35 mW 633 nm laser source with a right-angle geometry. Small-angle neutron scattering (SANS) experiments were performed using an extended Q-range small-angle neutron scattering diffractometer (EQ-SANS BL-6) with the High Flux Isotope Reactor (HFIR) located at Oak Ridge National Laboratory (ORNL). Two configurations (4m sample to detector distance with a wavelength band of λ_{min} = 12 Å and 2.5 m sample to detector distance with λ_{min} = 2.5 Å) were used to cover a wide range of scattering wavevector q. SANS measurements were performed on samples dissolved in o-DCB- d4 solution. Scattering data were reduced and corrected by subtracting the background from solvents and cells. The data were put on an absolute scale (cm^{-1}) by using a standard porous silica sample.

2. Syntheses

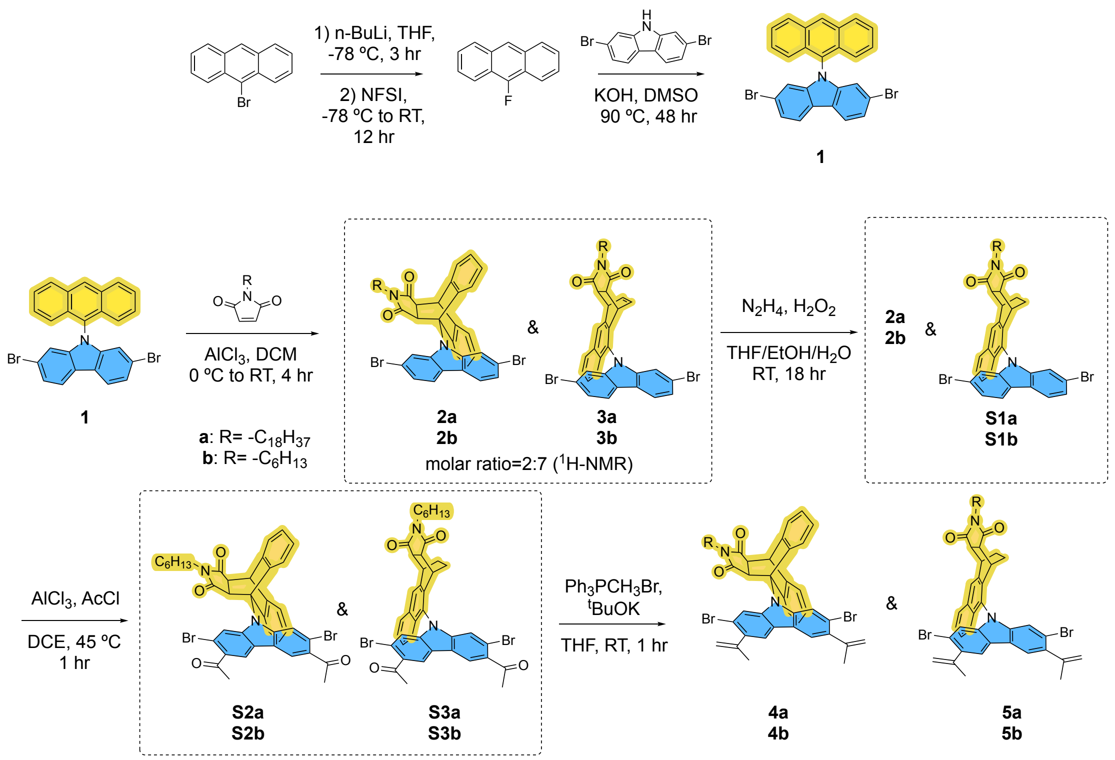

Scheme S1. Overall synthetic route for monomers **4** and **5**.

Step 1: Fluorination (modified from the reported procedures[4])

9-Bromoanthracene (5.0 g, 19.4 mmol) was dissolved in THF (65 ml) and the solution was cooled to -78 ºC prior to the addition of 1.6 M n-BuLi in hexane (15.2 ml, 24.3 mmol) dropwise. After stirring at -78 ºC for 3 hr, NFSI (8.0 g, 24.3 mmol) was added and the solution was stirred for 2 hr, before warming up to room temperature and stirred for another 12 hr. The mixture was quenched by 2 M $HCl_{(aq)}$ in an ice bath and extracted by EtOAc. Solvents of the organic layer were removed under reduced pressure. The crude was dissolved in DCM, dried over $MgSO_4$, and filtered through a short plug of SiO_2 (DCM as eluent). Solvents of the filtrate were removed under reduced pressure to afford 9-fluoroanthracene as a yellow solid, which was used for the next step immediately without further purification.

Step 2: S_NAr

2,7-dibromocarbazole (18.9 g, 58.2 mmol) and powder KOH (3.3 g, 58.2 mmol) were suspended in anhydrous DMSO (60 ml). The mixture was stirred at 90 ºC for 30 min prior to the addition of 9-fluoroanthracene portion wise. After stirring at 90 ºC for 48 hr, the solution was pour into MeOH and the precipitate was collected by filtration. The precipitate was further washed by a mixture of DCM/MeOH (1/1, v/v) before recrystallized from DMF to afford **1** as yellow crystals. (5.8 g, 60% yield over 2 steps). ^{1}H NMR (500 MHz, $CDCl_3$, 25 ºC) δ= 8.74 (s, 1H), 8.19 (d, J= 8.6 Hz, 2H), 8.08 (d, J= 8.3 Hz, 2H), 7.54 (m, 2H), 7.44 (dd, J= 8.3 Hz, 1.6 Hz, 2H), 7.35 (m, 2H), 7.17 (d, J= 8.8 Hz, 2H), 6.84 (d, J= 1.6 Hz, 2H). ^{13}C NMR (125 MHz, $CDCl_3$, 25 ºC) δ= 143.6, 132.2, 129.9, 129.2, 129.1, 127.8, 127.1, 126.1, 123.9, 122.9, 121.8, 121.7, 120.4, 113.6. APCI-HRMS: m/z $[M]^-$ Calcd for $C_{26}H_{S2b}r_2N$ 498.9571; Found 498.9580.

Synthesis of mixture 2a and 3a

1 (2.0 g, 4.0 mmol) and $AlCl_3$ (1.1 g, 8.2 mmol) were suspended in anhydrous DCM (30 ml) at 0 ºC before a solution of 1-octadecyl-1*H*-pyrrole-2,5-dione (1.4 g, 4.0 mmol) in CH_2Cl_2 (30 ml) was added. The reaction mixture was stirred at room temperature for 4 hr before quenched by 2 M $HCl_{(aq)}$ in an ice bath. The organic layer was further washed by 2 M $HCl_{(aq)}$ and saturated $NH_4Cl_{(aq)}$ and dried over $MgSO_4$. Volatile solvents were removed under reduced pressure. The crude product was purified through silica column chromatography (hexane/DCM: 2/1 to 1/1) to afford an inseparable mixture of **2a** and **3a** as a white solid (2.4 g, 72% yield). See **Figure S3** for ^{1}H NMR and **Figure S4** for ^{13}C NMR. APCI-HRMS: m/z $[M+H]^+$ Calcd for $C_{48}H_{55}Br_2N_2O_2$ 849.2630; Found 849.2605.

Synthesis of mixture 2b and 3b

Starting from **1** (1.0 g, 2.0 mmol), **2b** and **3b** were synthesized by the same method for **2a** and **3a** except 1-hexyl-1*H*-pyrrole-2,5-dione was used as the reagent. The crude product was purified through silica column chromatography (hexane/DCM: 1/2) to afford the title mixture as a white solid (814 mg, 60% yield). See **Figure S5** for ^{1}H NMR and **Figure S6** for ^{13}C NMR. APCI-HRMS: m/z $[M+H]^+$ Calcd for $C_{36}H_{31}Br_2N_2O_2$ 681.0747; Found 681.0728.

Synthesis of mixture 2a and S1a

The mixture of **2a** and **3a** (2.0 g, 2.4 mmol) was dissolved in THF (40 ml) before EtOH (20 ml) and 30% $H_2O_{2\,(aq)}$ (2.4 ml, 24 mmol) were added. The solution was cooled to 0 ºC and $N_2H_4{\cdot}H_2O$ (1.2 ml, 24 mmol) was added dropwise, and it was stirred at room temperature for 12 hr. Another portion of 30% $H_2O_{2\,(aq)}$ (1.2 ml, 12 mmol) and $N_2H_4{\cdot}H_2O$ (0.6 ml, 12 mmol) were added at 0 ºC, and the solution was stirred at room temperature for additional 2 hr. Volatile solvents are removed under reduced pressure. EtOAc was added, and the mixture was washed by H_2O and brine. The layer was dried over $MgSO_4$, and volatile solvents were removed under reduced pressure to afford a mixture of **2a** and **S1a** as a white solid (1.8 g, 90% yield). See **Figure S7** for ^{1}H NMR and **Figure S8** for ^{13}C NMR. **2a**: APCI-HRMS: m/z $[M+H]^+$ Calcd for $C_{48}H_{55}Br_2N_2O_2$ 849.2630; Found 849.2606. **S1a:** APCI-HRMS: m/z $[M+H]^+$ Calcd for $C_{48}H_{57}Br_2N_2O_2$ 851.2787; Found 851.2776.

Synthesis of mixture 2b and S1b

Starting from mixture **2b and 3b** (814 mg, 1.2 mmol), **2b** and **S1b** were synthesized as a white solid (650 mg, 80% yield) by the same method for **2a** and **S1a**. See **Figure S9** for ^{1}H NMR and **Figure S10** for ^{13}C NMR. **2b**: APCI-HRMS: m/z $[M+H]^+$ Calcd for $C_{36}H_{31}Br_2N_2O_2$ 681.0747; Found 681.0730. **S1b**: APCI-HRMS: m/z $[M+H]^+$ Calcd for $C_{36}H_{33}Br_2N_2O_2$ 683.0903; Found 683.0922.

Synthesis of mixture S2a and S3a

$AlCl_3$ (2.2 g, 16.6 mmol) was suspended in anhydrous 1,2-dichloroethane (20 ml) at room temperature, to which acetyl chloride (1.2 ml, 16.6 mmol) was added dropwise. After stirring for 5 min, a mixture of **2a** and **S1a** (1.8 g, 2.1 mmol) in 1,2-dichloroethane (10 ml) was added dropwise. The reaction mixture was further stirred at 45 ºC for 1 hr before quenched by 2 M $HCl_{(aq)}$ in an ice bath. The organic layer was further washed by 2 M $HCl_{(aq)}$, H_2O, and saturated $NH_4Cl_{(aq)}$ and dried over $MgSO_4$. After removing solvents under reduced pressure, the residue was purified through silica column chromatography (hexane/EtOAc: 12/1) to afford a mixture of **S2a** and **S3a** as a light yellow solid (1.8 g, 91% yield). See **Figure S11** for ^{1}H NMR and **Figure S12** for ^{13}C NMR. **S2a**: APCI-HRMS: m/z $[M+H]^+$ Calcd for $C_{52}H_{59}Br_2N_2O_4$ 933.2836; Found 933.2821. **S3a**: APCI-HRMS: m/z $[M+H]^+$ Calcd for $C_{52}H_{61}Br_2N_2O_4$ 935.2998; Found 935.2972.

Synthesis of mixture S2b and S3b

Starting from mixture **2b and S1b** (640 mg, 0.9 mmol), **S2b** and **S3b** were synthesized and by the same method for **S2a** and **S3a**. The crude product was purified through silica column chromatography (hexane/EtOAc: 2/1) to afford the title mixture as a light yellow solid (700 mg, 95% yield). See **Figure S13** for ^{1}H NMR and **Figure S14** for ^{13}C

NMR. **S2b**: APCI-HRMS: m/z $[M+H]^+$ Calcd for $C_{40}H_{35}Br_2N_2O_4$ 765.0958; Found 765.0937. **S3b**: APCI-HRMS: m/z $[M+H]^+$ Calcd for $C_{40}H_{37}Br_2N_2O_4$ 767.1115; Found 767.1088.

Synthesis of 4a and **5a**

Methyltriphenylphosphonium bromide (2.8 g, 7.7 mmol) and ^{t}BuOK (860 mg, 7.7 mmol), and several crystals of BHT were suspended in anhydrous THF (50 ml) at 0 ºC before a solution of **S2a** and **S3a** (1.8 g, 1.9 mmol) in THF (20 ml) was added dropwise. The reaction mixture was stirred at room temperature for 1 hr before quenched by 2 M $HCl_{(aq)}$ in an ice bath. The organic layer was further washed by brine and dried over $MgSO_4$. After removing the solvent under reduced pressure, the residue was purified through a short plug of silica (hexane/EtOAc: 3/1) and then through silica column chromatography (hexane/EtOAc: 15/1) to afford **5a** as a white solid (960 mg, 26 % yield over four steps from **1**) and semi-pure **4a**, which was further purified by recycling GPC to afford a white semi-solid (188 mg, 5% yield over four steps from **1**). **4a**: ^{1}H NMR (500 MHz, $CDCl_3$, 25 ºC) δ 8.10 (s, 1H), 7.99 (s, 1H), 7.94 (s, 1H), 7.51 (d, *J*= 7.4 Hz, 1H), 7.44 (d, *J*= 7.6 Hz, 1H), 7.27 (t, *J*= 7.6 Hz, 1H), 7.22 (t, *J*= 7.4 Hz, 1H), 7.13 (t, *J*= 7.6 Hz, 1H), 7.06 (d, *J*= 7.6 Hz, 1H), 6.91 (t, *J*= 7.4 Hz, 1H), 6.31 (s, 1H), 5.94 (d, *J*= 7.6 Hz, 1H), 5.32 (m, 1H), 5.26 (m, 1H), 5.09 (m, 1H), 5.02 (m, 1H), 4.96 (d, *J*= 2.8 Hz, 1H), 4.68 (d, *J*= 8.3 Hz, 1H), 3.36 (dd, *J*= 8.3 Hz, 2.6 Hz, 1H), 3.12–3.00 (m, 2H), 2.23 (s, 3H), 2.15 (s, 3H), 1.33–1.04 (m, 30H), 1.00–0.90 (m, 2H), 0.88 (t, *J*= 7.0 Hz, 3H), 0.75–0.63 (m, 2H). ^{13}C NMR (125 MHz, $CDCl_3$, 25 ºC) δ 175.2, 173.4, 146.5, 146.4, 141.8, 141.3, 139.6, 138.7, 137.1, 137.0, 136.2, 135.8, 128.2, 128.2, 127.0, 126.7, 126.5, 125.5, 124.2, 124.0, 123.9, 123.2, 121.0, 120.2, 120.0, 119.4, 118.9, 118.0, 116.4, 116.4, 69.8, 50.1, 48.8, 45.6, 39.2, 32.1, 29.8 (br), 29.7,

29.5, 29.5, 29.2, 27.2, 26.8, 24.3, 24.3, 22.8, 14.2. APCI-MS: m/z $[M+H]^+$ Calcd for $C_{54}H_{63}Br_2N_2O_2$ 929.3256; Found 929.3230. **5a**: ^1H NMR (500 MHz, $CDCl_3$, 25 ºC) δ 8.01 (3H, m), 7.97 (1H, s), 7.55 (1H, t, J=7.75 Hz), 7.34 (1H, t, J=7.75 Hz), 7.05 (1H, d, J=8.45 Hz), 7.00 (1H, s), 6.95 (1H, s), 5.32 (2H, m), 5.08 (2H, d, J=8.70 Hz), 3.83 (1H, m), 3.51 (2H, m), 3.12 (1H, m), 3.05 (1H, m), 2.73 (1H, m), 2.22 (3H, s), 2.21 (3H, s), 1.83 (1H, m), 1.71–1.57 (4H, m), 1.42 (1H, m), 1.24 (30H, m), 0.88 (3H, t, J=7.05 Hz). ^{13}C NMR (125 MHz, $CDCl_3$, 25 ºC) δ 178.2, 178.0, 146.6, 142.3, 142.2, 140.5, 139.5, 137.4, 137.3, 133.8, 130.4, 128.4, 127.5, 127.0, 126.8, 124.5, 123.3, 122.2, 121.5, 121.4, 120.0, 119.8, 116.5, 113.6, 113.5, 45.3, 44.7, 39.3, 35.8, 32.1, 30.9, 29.8, 29.7, 29.7, 29.6, 29.5, 29.3, 27.8, 27.1, 24.4, 22.8, 21.6, 14.2. APCI-HRMS: m/z $[M+H]^+$ Calcd for $C_{54}H_{65}Br_2N_2O_2$ 931.3407; Found 931.3382.

Note: starting from mixture **2a** and **3a** (2.0 g, 2.4 mmol), dihydroxylation was carried out for **3a** with $KMnO_4$ (575 mg, 3.6 mmol), NaOH (132 mg, 3.3 mmol), H_2O (6.2 ml), tert-amyl alcohol (6.2 ml), and THF (12.5 ml) at 0ºC for 2 hr. The significantly different R_f values of dehydroxylated **3a** and **2a** simplifies the purification processes. The reaction was quenched by saturated $Na_2S_2O_{3(aq)}$ and extracted by EtOAc. The organic layer was further washed by brine and dried over $MgSO_4$. After removing the solvent under reduced pressure, the residue was purified through a short plug of silica (hexane/DCM: 1/1) to afford pure **2a**, which was further converted to crude **4a** by procedures described above. Crude **4a** was purified through silica column chromatography (hexane/EtOAc: 15/1) without the need of recycling GPC. This method afford **4a** in 12% yield from **1** over four steps.

Synthesis of 4b and **5b**

Starting from mixture **S2b and S3b** (640 mg, 0.9 mmol), **4b** and **5b** were synthesized by the same method for **4a** and **5a**. The crude product was purified through silica column chromatography (hexane/EtOAc: 10/1) to afford **5b** as a white solid (271 mg, 17% yield over four steps from **1**) and semi-pure **4b**, which was further purified by recycling GPC to afford a white solid (80 mg, 5% yield over four steps from **1**). **4b**: ^{1}H NMR (500 MHz, $CDCl_3$, 25 ºC) δ= 8.10 (s, 1H), 7.99 (s, 1H), 7.94 (s, 1H), 7.51 (d, *J*= 7.4 Hz, 1H), 7.44 (d, *J*= 7.6 Hz, 1H), 7.27 (t, *J*= 7.6 Hz, 1H), 7.22 (t, *J*= 7.4 Hz, 1H), 7.13 (t, *J*= 7.6 Hz, 1H), 7.06 (d, *J*= 7.6 Hz, 1H), 6.91 (t, *J*= 7.4 Hz, 1H), 6.31 (s, 1H), 5.94 (d, *J*= 7.6 Hz, 1H), 5.32 (m, 1H), 5.26 (m, 1H), 5.09 (m, 1H), 5.02 (m, 1H), 4.96 (d, *J*= 2.8 Hz, 1H), 4.68 (d, *J*= 8.3 Hz, 1H), 3.36 (dd, *J*= 8.3 Hz, 2.6 Hz, 1H), 3.12–3.00 (m, 2H), 2.23 (s, 3H), 2.15 (s, 3H), 1.17 (m, 2H), 1.07 (m, 2H), 0.95 (m, 2H), 0.82 (t, *J*= 7.3 Hz, 3H), 0.70 (m, 2H). ^{13}C NMR (125 MHz, $CDCl_3$, 25 ºC) δ 175.2, 173.4, 146.5, 146.4, 141.8, 141.3, 139.6, 138.7, 137.1, 137.0, 136.2, 135.8, 128.2, 128.2, 127.0, 126.7, 126.5, 125.5, 124.2, 124.0, 123.9, 123.2, 121.0, 120.2, 120.0, 119.4, 118.9, 118.0, 116.4, 116.4, 69.8, 50.1, 48.8, 45.6, 39.2, 31.3, 27.1, 26.5, 24.3, 24.3, 22.4, 14.1. APCI-HRMS: m/z $[M+H]^+$ Calcd for $C_{42}H_{39}Br_2N_2O_2$ 761.1378; Found 761.1350. **5b**: ^{1}H NMR (500 MHz, $CDCl_3$, 25 ºC) δ 8.01 (3H, m), 7.97 (1H, s), 7.55 (1H, t, J=7.75 Hz), 7.34 (1H, t, J=7.75 Hz), 7.05 (1H, d, J=8.45 Hz), 7.00 (1H, s), 6.95 (1H, s), 5.32 (2H, m), 5.08 (2H, d, J=8.70 Hz), 3.83 (1H, m), 3.51 (2H, m), 3.12 (1H, m), 3.05 (1H, m), 2.73 (1H, m), 2.22 (3H, s), 2.21 (3H, s), 1.83 (1H, m), 1.71–1.57 (4H, m), 1.42 (1H, m), 1.24 (6H, m), 0.88 (3H, t, J=7.05 Hz). ^{13}C NMR (125 MHz, $CDCl_3$, 25 ºC) δ 178.2, 178.0, 146.6, 146.6, 142.3, 142.2, 140.5, 139.5, 137.4, 137.3, 133.8, 130.4, 128.4, 127.5, 127.0, 126.8, 124.5, 123.3, 122.2, 121.5, 121.4, 120.0, 119.8, 116.5, 113.6, 113.5, 45.3, 44.7, 39.2, 35.8, 31.4, 30.9, 27.7, 26.7, 24.4, 22.6, 21.6, 14.1. APCI-HRMS: m/z $[M+H]^+$ Calcd for $C_{42}H_{41}Br_2N_2O_2$ 763.1529; Found 763.1538.

Synthesis of 6

4b (34 mg, 44 μmol), 4,4,5,5-tetramethyl-2-(2-vinylphenyl)-1,3-dioxolane (26 mg, 110 μmol), freshly prepared $Pd(PPh_3)_4$ (2.5 mg, 2.2 μmol), K_2CO_3 (36 mg, 260 μmol), aliquat 336 (4.5 mg, 11 μmol), several crystals of BHT, toluene (1.25 ml), and water (0.60 ml) were added into a 25 ml Schlenk flask. The solution was degassed by freeze-pump-thaw for three cycles before stirring at 100 ºC for 18 hr. After cooling to room temperature, EtOAc was added. The organic layer was washed by water and brine, and it was dried over $MgSO_4$ and passed through a short plug of SiO_2 (hexane/EtOAc: 2/1). After removing solvents under reduced pressure, the residue was purified through silica column chromatography (hexane/EtOAc: 7/1) to afford **6** as a white solid (25 mg, 70%). ^{1}H NMR (500 MHz, $CDCl_3$, 25 ºC) δ= 8.21 (s, 0.5H), 8.19 (s, 0.4H), 8.13 (s, 1H), 7.70–7.58 (m, 2H), 7.47–7.36 (m, 2H), 7.36–7.27 (m, 3H), 7.24–7.19 (m, 1H), 7.19–7.09 (m, 4H), 7.09–6.94 (m, 3H), 6.94–6.85 (m, 1H), 6.85–6.75 (br, 0.3H), 6.58–6.49 (m, 0.4H), 6.48–6.33 (br, 0.3H), 6.27–6.14 (br, 1H), 6.06–5.89 (br, 1.5H), 5.61 (t, *J*= 17.3 Hz, 1 H), 5.48–5.17 (br, 1H), 5.13–4.92 (m, 4H), 4.92–4.80 (m, 2H), 4.75–4.65 (m, 1.5H), 3.25–3.19 (m, 1H), 3.05–2.90 (m, 2H), 1.80 (s, 1.2H), 1.77 (s, 1.7H), 1.68–1.64 (m, 1H), 1.22–1.13 (m, 2H), 1.13–1.02 (m, 2H), 1.00–0.88 (m, 2H), 0.83 (t, *J*= 7.3 Hz, 3 H) 0.75–0.60 (m, 2H). ^{13}C NMR (125 MHz, $CDCl_3$, 25 ºC) δ 175.4, 173.3, 172.9, 146.6, 146.3, 146.0, 142.5, 142.0, 141.3, 141.3, 140.5, 139.7, 139.1, 137.0, 136.4, 136.3, 136.2, 136.2, 136.1, 136.1, 135.9, 135.7, 135.6, 135.3, 135.2, 131.3, 130.8, 127.9, 127.4, 127.3, 127.0, 126.9, 126.7, 126.5, 125.2, 124.8, 124.5, 124.3, 124.1, 123.9, 123.5, 119.7, 119.4, 119.2, 117.3, 117.0, 116.1, 115.9, 115.8, 114.0, 113.9, 69.5, 50.0, 49.9, 48.8, 48.7, 45.6, 38.9, 31.3, 29.8, 27.1, 27.1, 26.5, 26.5, 24.0, 22.5.

Resonance signals are broaden due to hindered rotation. APCI-MS: m/z $[M+H]^+$ Calcd for $C_{58}H_{53}N_2O_2$ 809.4102; Found 809.4078.

Synthesis of 7

6 (16 mg, 20 μmol) and a few crystals of BHT were dissolved in toluene (1.0 ml) in a 25 ml Schlenk flask, and Grubbs 2nd generation catalyst (0.84 mg, 1 μmol, 5 mol%) was dissolved in toluene (0.5 ml) in a separated flask. Both solutions were degassed by freeze-pump-thaw for three cycles. The flask containing **6** was stirred under reflux temperature, while the catalyst solution was injected by syringe pump over 6 hr. During the course of the reaction, four additional batches of catalyst solutions (5 mol% for each) were added by the same way in every 12 hr. After 12 hr from the final addition of catalyst solution, toluene was removed under reduced pressure, and the crude mixture was filtered through a plug of silica (DCM as eluent). The filtrate was dried under reduced pressure, and the resulting solid was further washed by MeOH to afford **7** as a yellow solid (14.5 mg, 90% yield). ^{1}H NMR (400 MHz, $CDCl_3$, 25 °C) δ= 9.35 (s, 1H), 9.08 (s, 1H), 9.02 (s, 1H), 8.48 (dd, *J*= 7.9 Hz, 1.5 Hz, 1H), 7.84 (dd, 7.2 Hz, 2.0 Hz, 1H), 7.70 (d, *J*= 7.8 Hz, 1H), 7.62–7.49 (m, 5H), 7.48 (s, 1H), 7.42 (s, 1H), 7.40–7.31 (m, 3H), 7.29–7.26 (m, 1H), 7.14 (t, *J*= 7.5 Hz, 2H), 7.05 (td, *J*= 7.7 Hz, 1.0 Hz, 1H), 6.69 (td, *J*= 7.7 Hz, 1.0 Hz, 1H), 6.06 (d, *J*= 7.9 Hz, 1H), 5.24 (d, *J*= 8.4 Hz, 1H), 5.12 (d, *J*= 2.8 Hz, 1H), 3.56 (dd, *J*= 8.3 Hz, 2.8 Hz, 1H), 3.21–3.08 (m, 2H), 3.03 (s, 3H), 2.99 (s, 3H), 1.22–0.92 (m, 6H), 0.86–0.68 (m, 5H). ^{13}C NMR (125 MHz, $CDCl_3$, 25 °C) δ= 175.5, 173.6, 143.2, 142.4, 140.0, 139.1, 136.9, 135.9, 132.9, 132.9, 132.6, 132.2, 130.4, 130.1, 129.9, 128.8, 128.2, 128.1, 128.0, 127.8, 127.3, 126.8, 126.7, 126.6, 126.5, 126.3, 126.0, 125.6, 125.5, 125.5, 125.3, 125.0, 124.8, 124.7, 124.0, 122.5, 122.2, 115.9, 115.5, 110.5, 107.3, 70.1, 50.5, 49.3, 45.8, 39.2, 31.3, 27.1,

26.5, 22.4, 20.7, 20.7, 14.1. APCI-HRMS: m/z $[M+H]^+$ Calcd for $C_{54}H_{45}N_2O_2$ 753.3481; Found 753.3470. Single crystals suitable for X-ray diffraction were grown from slow evaporation of its $CHCl_3$/MeCN solution.

Synthesis of 8

5b (80 mg, 0.1 mmol), 4,4,5,5-tetramethyl-2-(2-vinylphenyl)-1,3-dioxolane (70 mg, 0.3 mmol), freshly prepared $Pd(PPh_3)_4$ (11.5 mg, 10 μmol), K_2CO_3 (83 mg, 0.6 mmol), aliquat 336 (10 mg, 25 μmol), several crystals of BHT, toluene (2.8 ml), and water (0.60 ml) were added into a 25 ml Schlenk flask. The solution was degassed by freeze-pump-thaw for three cycles before stirring at 100 ºC for 4 hr. After cooling to room temperature, EtOAc was added. The organic layer was washed by water and brine, and it was dried over $MgSO_4$ and passed through a short plug of SiO_2 (hexane/EtOAc: 2/1). After removing solvents under reduced pressure, the residue was purified through silica column chromatography (hexane/EtOAc: 7/1) to afford **8** as a white solid (60 mg, 70%). ^{1}H NMR (500 MHz, $CDCl_3$, 25 ºC) δ= 8.20 (m, 2H), 7.88 (d, J= 8.4 Hz, 1H), 7.81 (s, 1H), 7.57–7.42 (m, 3H), 7.32–7.27 (m, 1H), 7.25–7.10 (m, 7H), 6.66–6.33 (m, 4H), 5.60–5.36 (m, 2H), 5.08–4.81 (m, 6H), 3.71 (m, 1H), 3.53 (m, 2H), 3.34–3.12 (m, 1H), 2.97–2.87 (m, 1H), 2.84–2.64 (m, 1H), 1.78 (s, 6H), 1.76–1.56 (m, 3H), 1.52–1.40 (m, 1H), 1.30 (m, 8H), 0.88 (m, 3H). ^{13}C NMR (125 MHz, $CDCl_3$, 25 ºC) δ 178.4, 178.2, 177.9, 146.5, 146.5, 141.8, 141.8, 141.6, 141.5, 141.4, 140.3, 139.8, 139.5, 139.3, 139.0, 137.2, 137.1, 136.5, 136.4, 136.3, 136.1, 136.0, 135.8, 135.8, 135.6, 133.7, 131.0, 130.8, 130.7, 128.2, 127.8, 127.7, 127.3, 127.0, 126.8, 126.6, 124.8, 123.9, 123.7, 123.6, 122.5, 122.5, 122.4, 120.7, 120.7, 116.1, 116.0, 114.3, 114.2, 114.1, 114.0, 112.1, 112.0, 111.8, 111.8, 111.6, 45.3, 44.7, 44.6, 39.1, 35.7, 31.4, 30.9, 30.8,

27.7, 26.7, 24.2, 22.6, 21.5, 14.1. Resonance signals are broaden due to hindered rotation. APCI-MS: m/z $[M+H]^+$ Calcd for $C_{58}H_{55}N_2O_2$ 811.4264; Found 811.4241.

Synthesis of 9

8 (47 mg, 58 μmol) and Grubbs 2nd generation catalyst (4.9 mg, 5.8 μmol, 10 mol%) were dissolved in DCM (6.0 ml) in a 35 ml pressure tube. The solution was purged by N_2 for 15 min before stirred at 70 ºC. After reacting for 18 hr, another batch of Grubbs 2nd generation catalyst (2.5 mg, 2.9 μmol, 5 mol%) was added. (Note: catalyst was added at room temperature and the solution was purged by N_2 for 15 min before heated). After 12 hr from the final addition of catalyst, toluene was removed under reduced pressure, and the crude mixture was filtered through a plug of silica (DCM as eluent). The filtrate was dried under reduced pressure, and the resulting solid was further washed by MeOH to afford **9** as a yellow solid (40 mg, 85% yield). ^{1}H NMR (500 MHz, $CDCl_3$, 25 ºC) δ= 9.11 (s, 1H), 9.10 (s, 1H), 8.28 (d, *J*= 8.3 Hz, 1H), 8.24 (d, *J*= 8.3 Hz, 1H), 8.15–8.10 (m, 2H), 8.06 (s, 1H), 8.01 (s, 1H), 7.80 (d, *J*= 8.0 Hz, 2H), 7.60–7.54 (m, 3H), 7.51–7.45 (m, 2H), 7.42–7.34 (m, 3H), 7.31–7.26 (m, 1H), 3.94–3.90 (m, 1H), 3.47–3.36 (m, 2H), 3.25–3.21 (m, 1H), 3.14–3.10 (m, 1H), 3.04 (s, 6H), 2.76–2.72 (m, 1H), 1.88–1.81 (m, 1H), 1.78–1.70 (m, 1H), 1.50–1.43 (m, 2H), 1.24–1.16 (m ,8H), 0.79 (t, *J*= 6.5 Hz, 3H). ^{13}C NMR (125 MHz, $CDCl_3$, 25 ºC) δ= 178.3, 177.5, 143.7, 143.6, 141.0, 139.9, 134.2, 133.4, 133.3, 132.4, 132.4, 130.9, 130.8, 129.7, 128.5, 128.1, 128.0, 127.4, 127.2, 127.1, 127.0, 126.7, 125.4, 125.3, 124.6, 124.4, 124.3, 123.8, 122.6, 122.6, 117.2, 117.1, 101.5, 101.5, 45.4, 44.7, 39.1, 35.9, 31.4, 31.2, 29.8, 27.6, 26.6, 22.5, 21.8, 21.7, 21.0, 14.0. APCI-HRMS: m/z $[M+H]^+$ Calcd for $C_{54}H_{47}N_2O_2$ 755.3638; Found 755.3618. Single crystals suitable for X-ray diffraction were grown from slow evaporation of its $CHCl_3$/MeCN solution.

Synthesis of 10

(^{13}C-Methyl)triphenylphosphonium iodide (1.2 g, 3.0 mmol), KO^{t}Bu (335 mg, 3.0 mmol), and several crystals of BHT were dissolved in anhydrous THF (24 ml) at 0 ºC before 2,5-dibromoterephthalaldehyde (420 mg, 1.4 mmol) was added in portion. The mixture was then stirred at room temperature for 12 hr before quenched by 2M $HCl_{(aq)}$ and washed by H_2O and brine. The organic layer was dried over $MgSO_4$ and filter through a plug of silica (hexanes as eluent). Volatile solvents were removed under reduced pressure to afford the crude, which as further purified through silica column chromatography (hexane) to afford ^{13}C-1,4-dibromo-2,5-divinylbenzene as a white solid (400 mg, 95% yield).

^{13}C-1,4-dibromo-2,5-divinylbenzene (400 mg, 1.4 mmol), B_2pin_2 (1.0 g, 3.9 mmol), Pd_2dba_3 (64 mg, 70 μmol), SPhos (70 mg, 170 μmol), KOAc (815 mg, 8.3 mmol), several crystals of BHT, and isopropylacetate (4.5 ml) were added into a 25 ml Schlenk flask. The solution was degassed by freeze-pump-thaw for three cycles before stirring at 55 ºC for 12 hr. After cooling to room temperature, the mixture was washed by water and brine. Volatile solvents of the organics were removed under reduced pressure to afford the crude solid, which was washed by cold MeOH and sonicated for three times. The solid was further dissolved a mixture of hexanes/DCM (2/1, v/v), dried over $MgSO_4$, and passed through a short plug of SiO_2 (hexanes/DCM: 2/1). Solvents of the filtrate were removed under reduced pressure to afford **11** as a white solid (265 mg, 50% yield). Analytical data is in good agreement with the literature[5].

Synthesis of P1

4a (127.1 mg, 0.14 mmol), **10** (52.4 mg, 0.14 mmol), freshly prepared $Pd(PPh_3)_4$ (15.7 mg, 14 μmol, 10 mol%), K_2CO_3 (115 mg, 0.82 mmol), aliquat 336 (13.7 mg, 34 μmol), a few crystals of BHT, toluene (3.9 ml), and water (0.8 ml) were added into a 25 ml Schlenk flask. The solution was degassed by freeze-pump-thaw for three cycles before allowing to stir at 100 ºC for 24 hr. End-capping was carried out at 85 ºC for 12 hr with 2-bromostyrene (99 mg, 0.54 mmol) and another 12 hr with 2-vinylphenylboronic acid (160 mg, 1.08 mmol). After cooling down to room temperature, the polymer was precipitated from methanol (50 ml), collected by centrifugation, and redissolved in DCM (3 ml) for re-precipitation for three cycles. The collected precipitate was redissolved in DCM and filtered, and MeOH and BHT were added to the filtrate to afford a suspension. Volatiles solvents were removed under reduced pressure to afford **P1** (125 mg, 99% yield, M_n= 8.2 kg/mol, $Đ$= 2.02). See **Figure S36** for ^{1}H NMR spectrum and **Figure S56a** for SEC chromatogram.

Synthesis of P2

5a (153.7 mg, 0.16 mmol), **10** (63.0 mg, 0.16 mmol), freshly prepared $Pd(PPh_3)_4$ (19 mg, 16 μmol, 10 mol%), K_2CO_3 (137 mg, 0.99 mmol), aliquat 336 (17 mg, 42 μmol), BHT, toluene (4.7 ml), and water (1.0 ml) were added into a 25 ml Schlenk flask. The solution was degassed by freeze-pump-thaw for three cycles before allowing to react at 100 ºC for 24 hr. End-capping was carried out at 85 ºC for 12 hr with 2-bromostyrene (120 mg, 0.65 mmol) and another 12 hr with 2-vinylphenylboronic acid (195 mg, 1.32 mmol). After cooling down to room temperature, the polymer was precipitated from methanol (50 ml), collected by centrifugation, and redissolved in DCM (3 ml) for re-precipitation for three cycles. The collected precipitate was redissolved in DCM and filtered, and MeOH and BHT were added to the filtrate to afford a suspension. Volatiles

solvents were removed under reduced pressure to afford **P2** (150 mg, 98% yield, M_n= 10.7 kg/mol, *Đ*= 2.42). See **Figure S37** for ^{1}H NMR spectrum and **Figure S56b** for SEC chromatogram.

Synthesis of LP1

P1 (120 mg, 0.13 mmol (repeating unit)) and a few crystals of BHT were dissolved in toluene (13 ml) in a 50 ml Schlenk flask, and Grubbs 2nd generation catalyst (5.6 mg, 6.5 μmol, 5 mol%) was dissolved in toluene (1.0 ml) in a separated flask. Both solutions were degassed by freeze-pump-thaw for three cycles. The flask containing **P1** was then stirred under reflux temperature, while the catalyst solution was injected by syringe pump over 6 hr. During the course of the reaction, four additional batches of catalyst solutions (5 mol% for each) were added by the same way in every 12 hr. After 12 hr from the final addition of catalyst solution, the polymer was precipitated from methanol (50 ml) and collected via centrifugation (115 mg, 99% yield). Analytical samples for SANS were prepared by fractionation using recycling SEC ($CHCl_3$ as eluent). The molar mass distribution is summarized in **Table S*1***.

Table S1. Molar masses and dispersity of **LP1** fractions

Fraction	M_n (kg/mol)	M_w (kg/mol)	*Đ*	quantity	yield
**LP1a**	16.7	23.3	1.4	24 mg	20%
**LP1b**	10.0	15.8	1.6	30 mg	25%
**LP1c**	3.0	3.6	1.2	20 mg	18%

Synthesis of LP2

P2 (140 mg, 0.16 mmol (repeating unit)), a few crystals of BHT, and Grubbs 2nd generation catalyst (6.8 mg, 8.0 μmol, 5 mol%) were dissolved in 1,2-DCE (12 ml) in

a 50 ml Schlenk flask. The solution was degassed by freeze-pump-thaw for three cycles and stirred at reflux temperature. After reacting for 12 hr, four more batches of Grubbs 2nd generation catalyst (6.8 mg, 8.0 μmol, 5 mol% for each) were added in every 12 hr. After 12 hr from the final addition of catalyst, the reaction mixture was cooled to room temperature. The polymer was then precipitated from methanol (50 ml) and collected via centrifugation (140 mg, 99% yield). Analytical samples for SANS were prepared by fractionation using recycling SEC ($CHCl_3$ as eluent). The molar mass distribution is summarized in **Table S*2***.

Table S2. Molar masses and dispersity of **LP2** fractions

Fraction	M_n (kg/mol)	M_w (kg/mol)	*Đ*	quantity	yield
**LP2a**	16.2	21.1	1.3	30 mg	20 %
**LP2b**	10.1	16.2	1.6	50 mg	36 %

3. NMR spectra

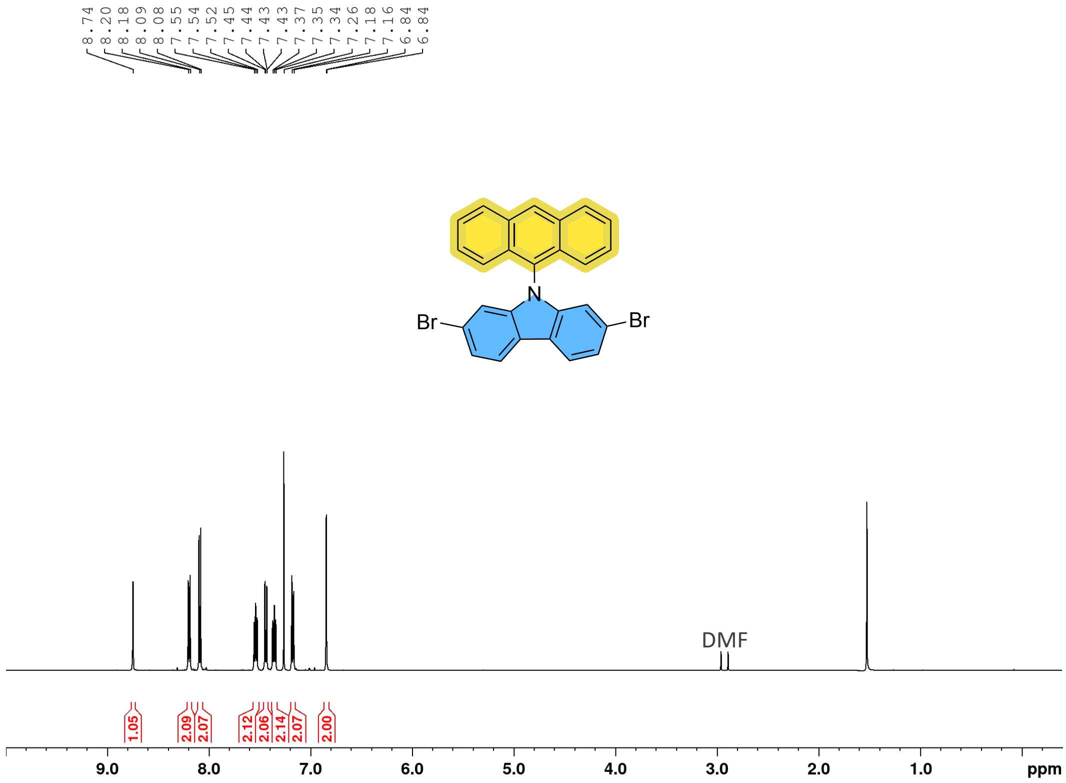

Figure S1. ^{1}H NMR (500 MHz, $CDCl_3$, 25 ºC) spectrum of **1**.

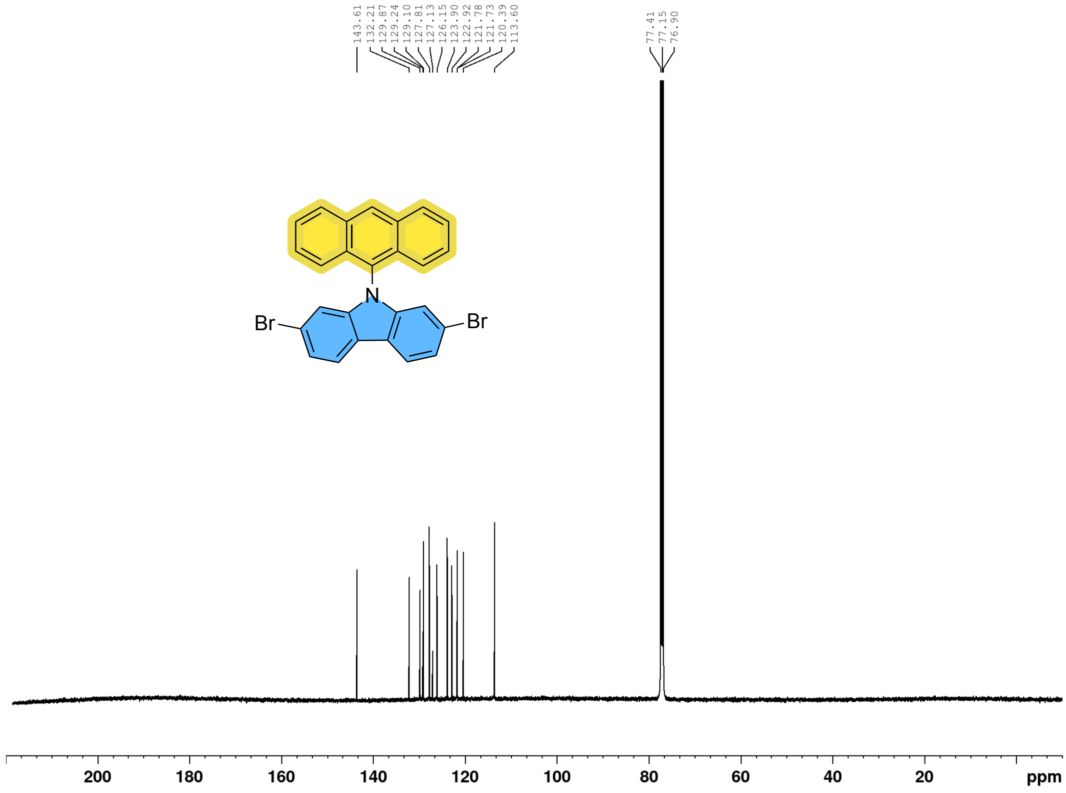

Figure S2. ^{13}C NMR (125 MHz, $CDCl_3$, 25 ºC) spectrum of **1**.

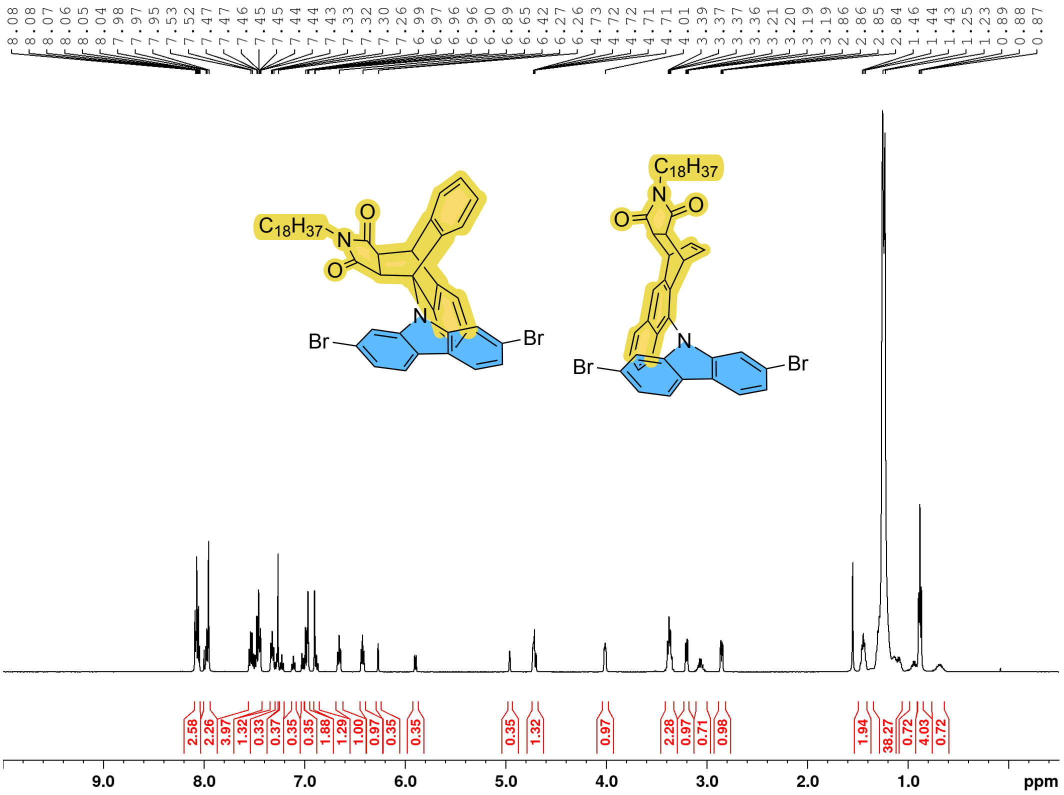

Figure S3. ^{1}H NMR (500 MHz, $CDCl_3$, 25 ºC) spectrum of mixture **2a** and **3a**.

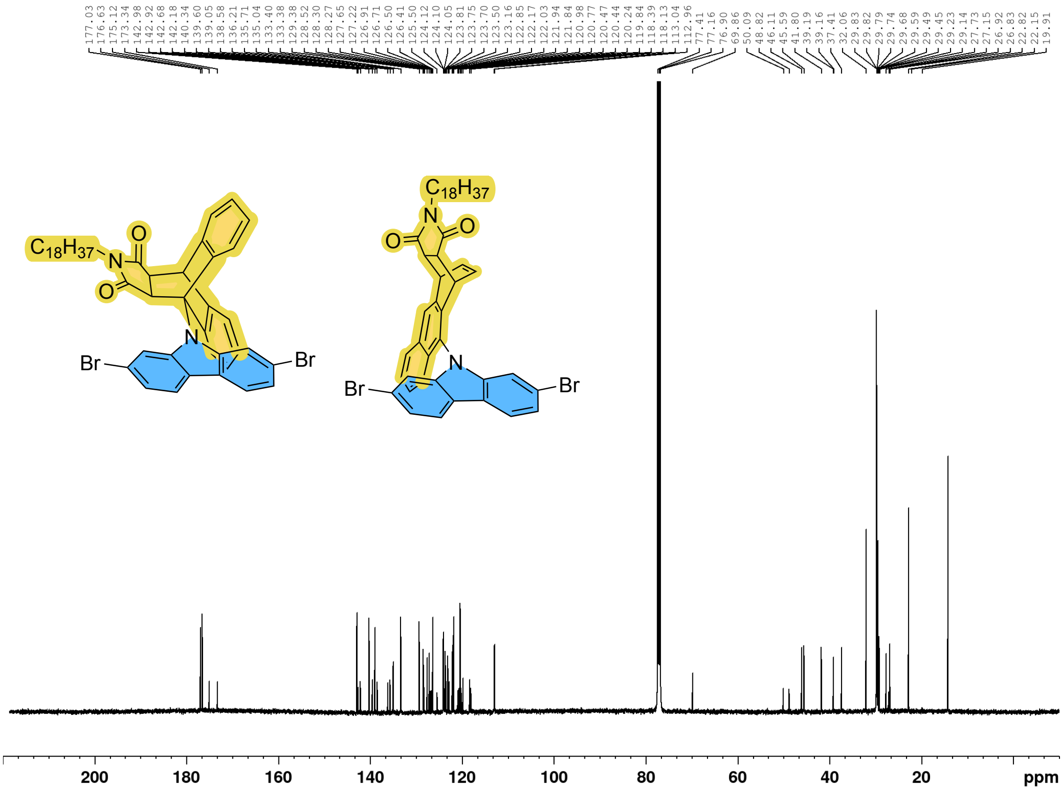

Figure S4. ^{13}C NMR (125 MHz, $CDCl_3$, 25 ºC) spectrum of mixture **2a** and **3a**.

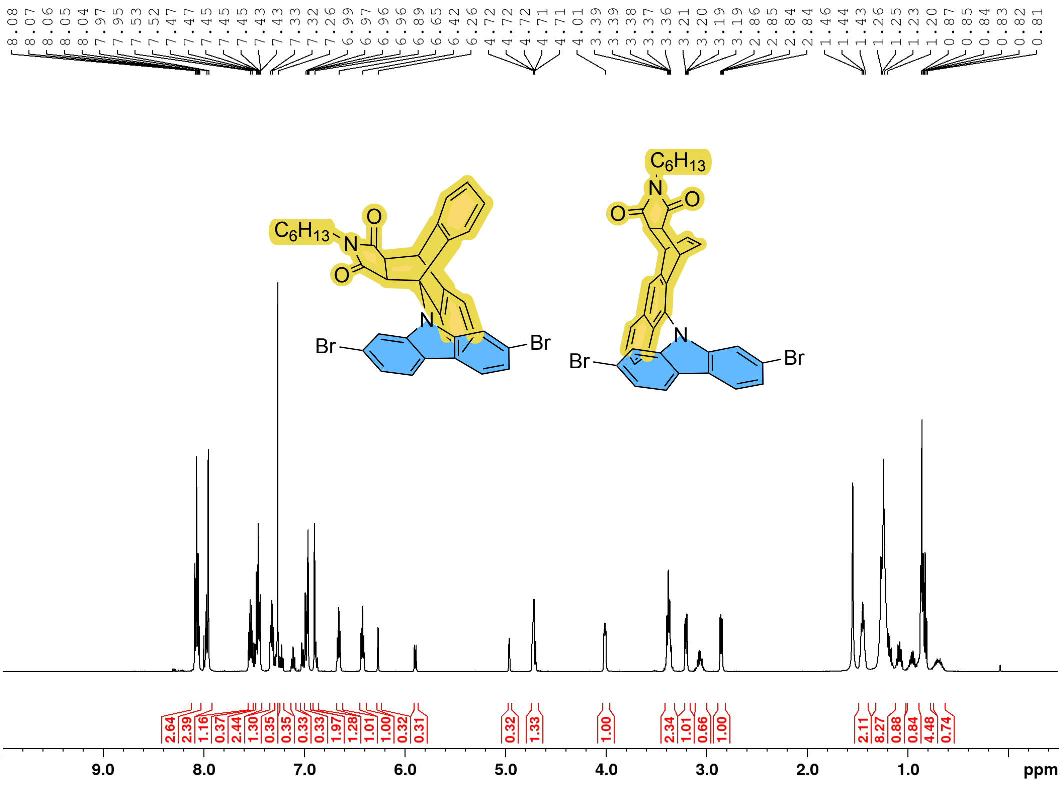

Figure S5. ^{1}H NMR (500 MHz, $CDCl_3$, 25 ºC) spectrum of mixture **2b** and **3b**.

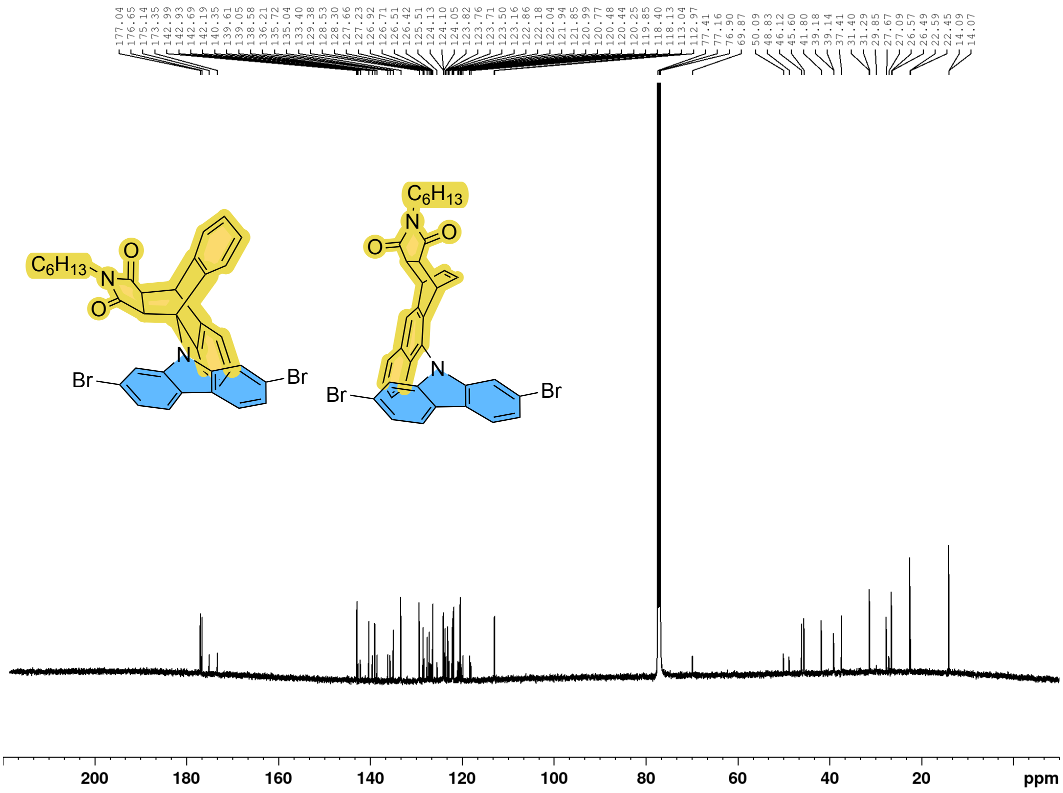

Figure S6. ^{13}C NMR (125 MHz, $CDCl_3$, 25 ºC) spectrum of mixture **2b** and **3b**.

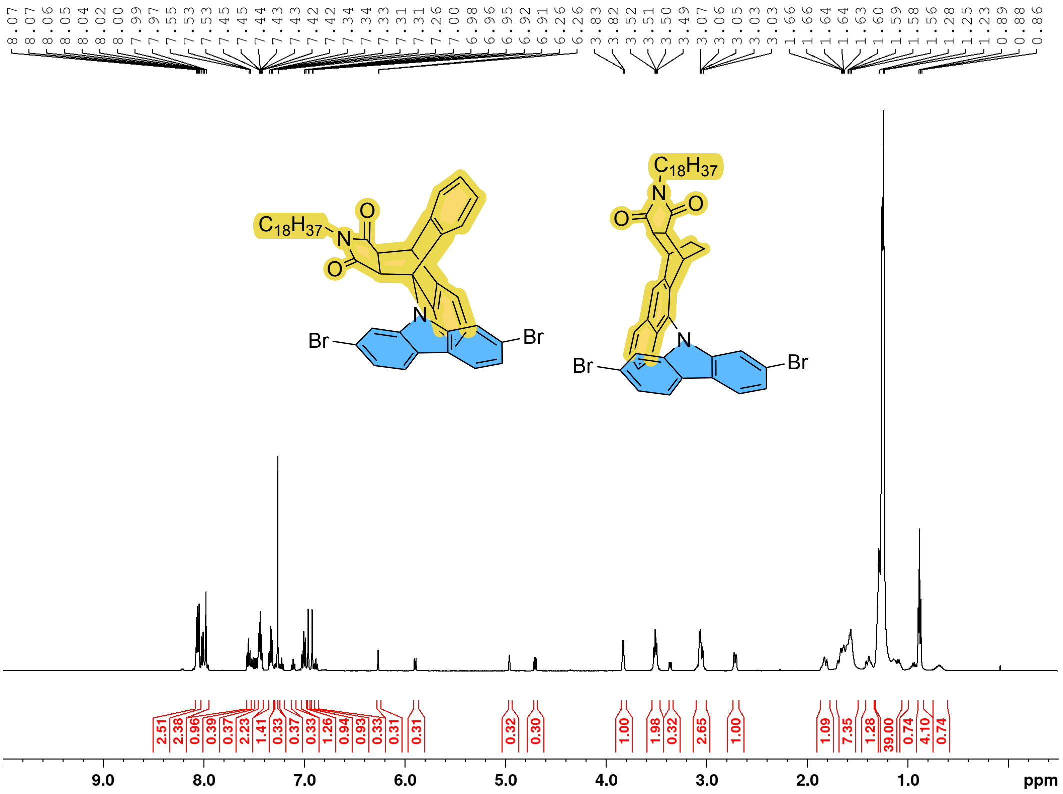

Figure S7. ^{1}H NMR (500 MHz, $CDCl_3$, 25 °C) spectrum of mixture **2a** and **S1a**.

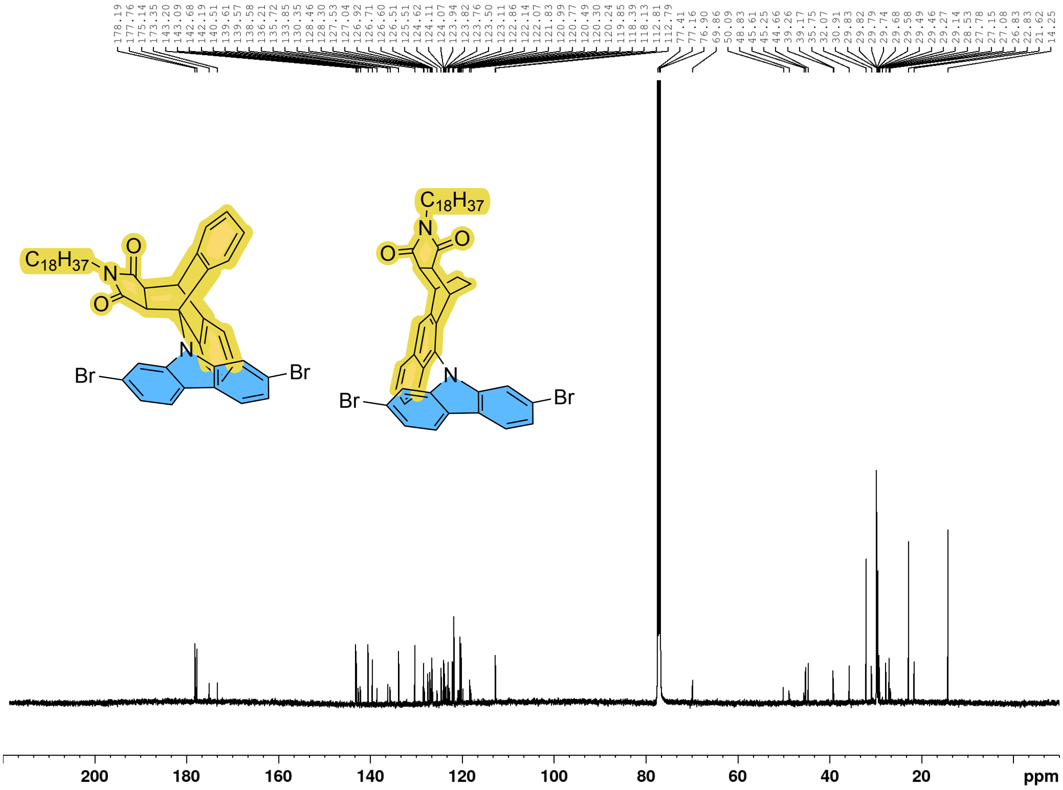

Figure S8. ^{13}C NMR (125 MHz, $CDCl_3$, 25 °C) spectrum of mixture **2a** and **S1a**.

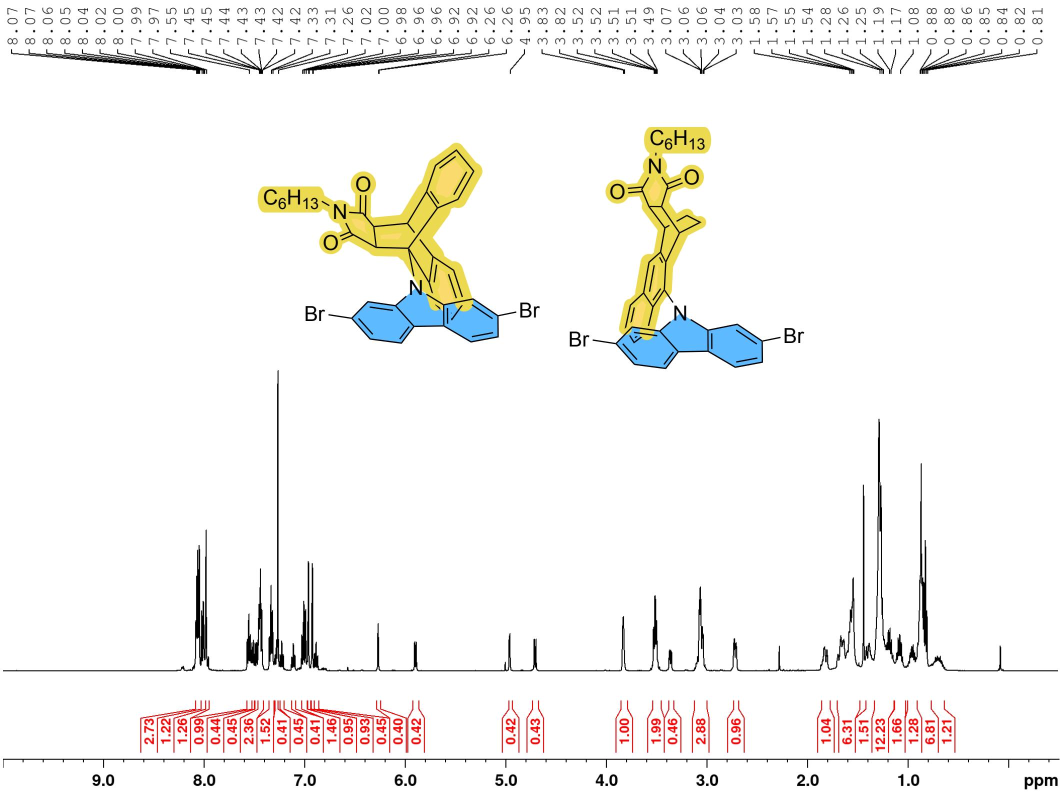

Figure S9. ^{1}H NMR (500 MHz, $CDCl_3$, 25 ºC) spectrum of mixture **2b** and **S1b**.

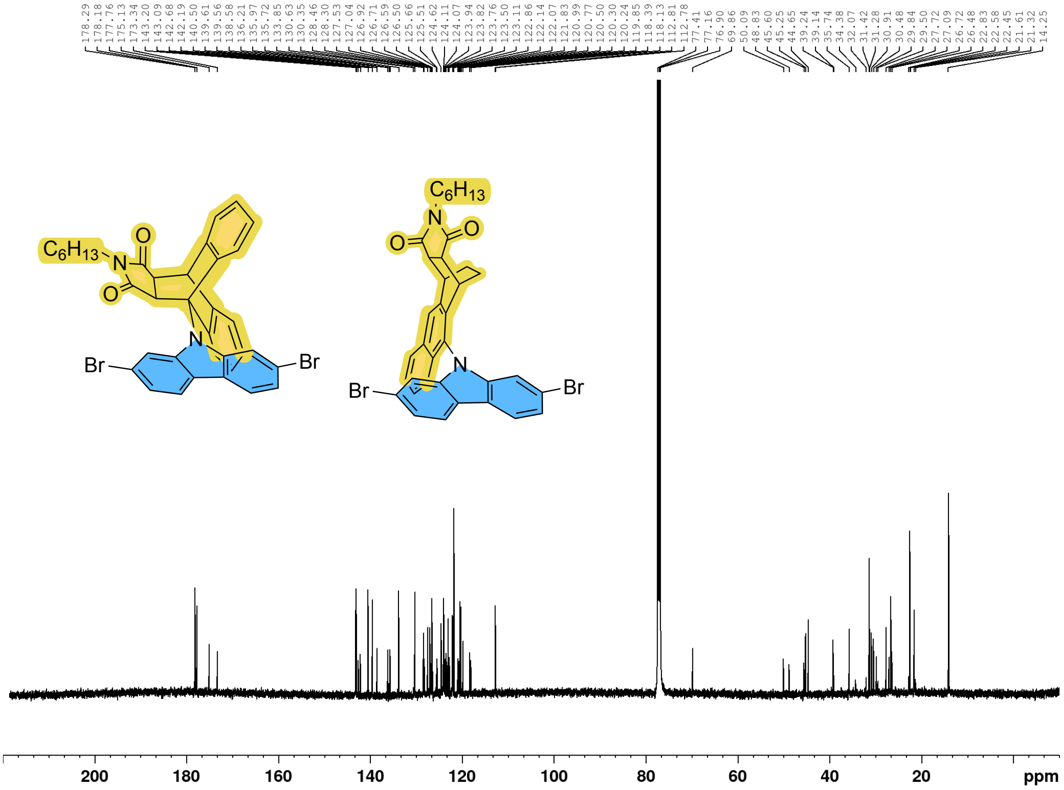

Figure S10. ^{13}C NMR (125 MHz, $CDCl_3$, 25 ºC) spectrum of mixture **2b** and **S1b**.

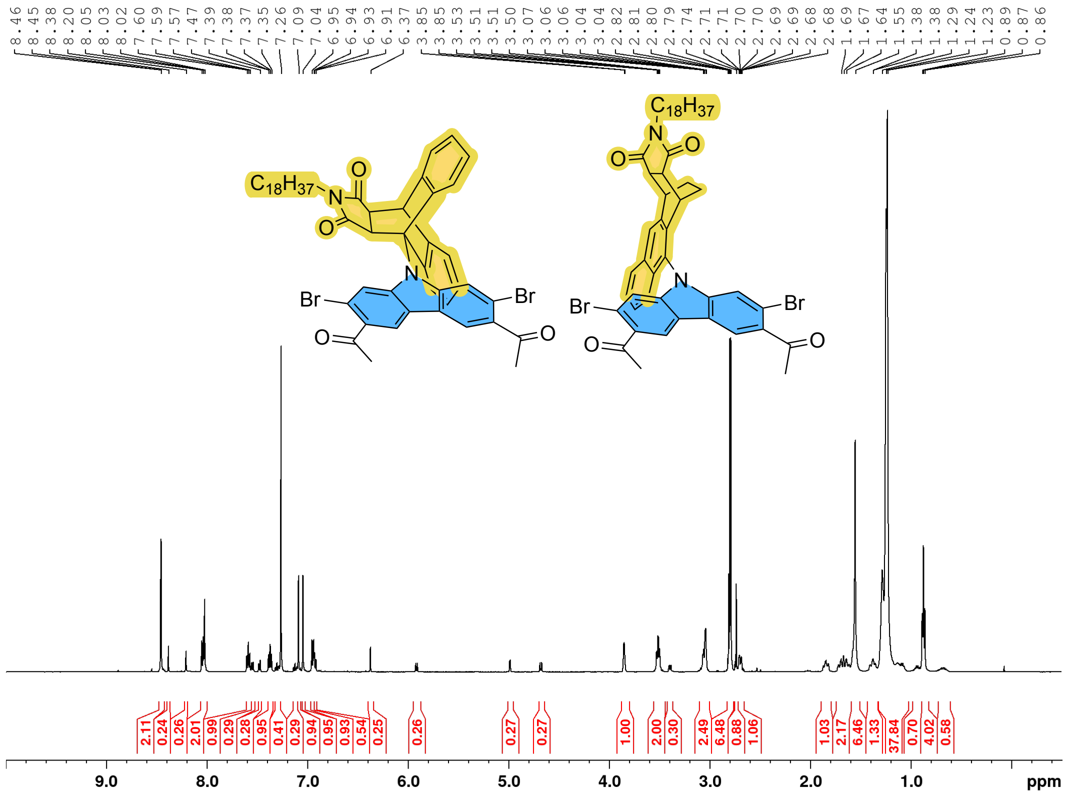

Figure S11. ^{1}H NMR (500 MHz, $CDCl_3$, 25 ºC) spectrum of mixture **S2a** and **S3a**.

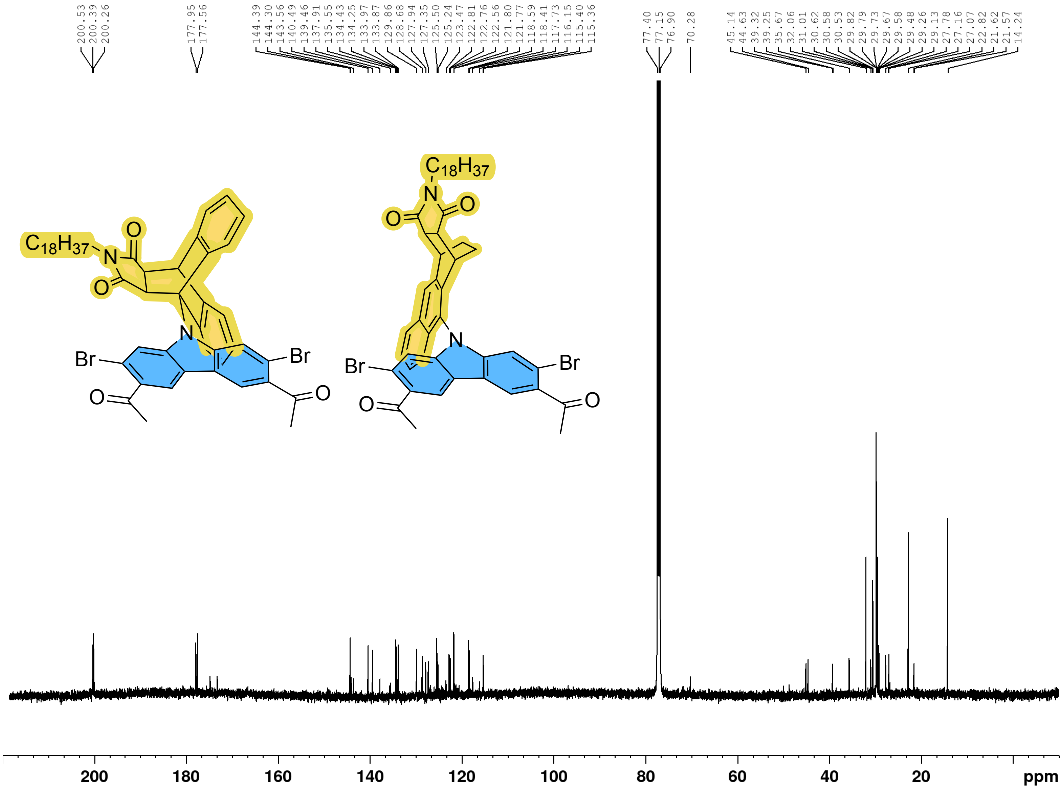

Figure S12. ^{13}C NMR (125 MHz, $CDCl_3$, 25 ºC) spectrum of mixture **S2a** and **S3a**.

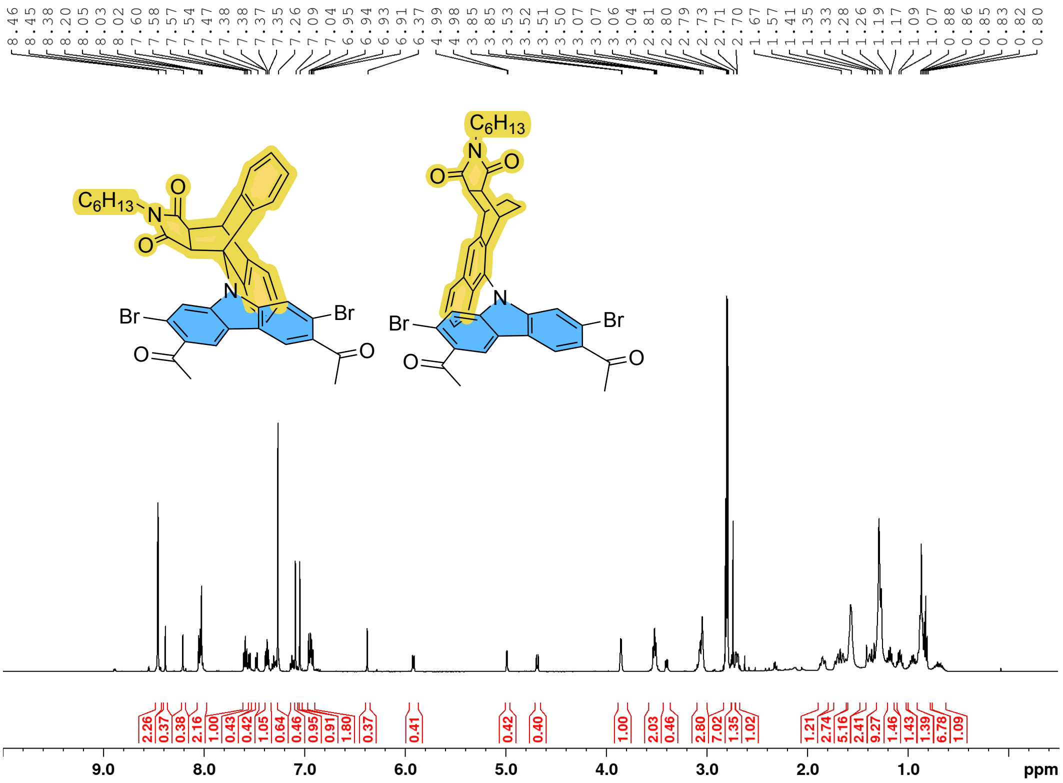

Figure S13. ^{1}H NMR (500 MHz, $CDCl_3$, 25 ºC) spectrum of mixture **S2b** and **S3b**.

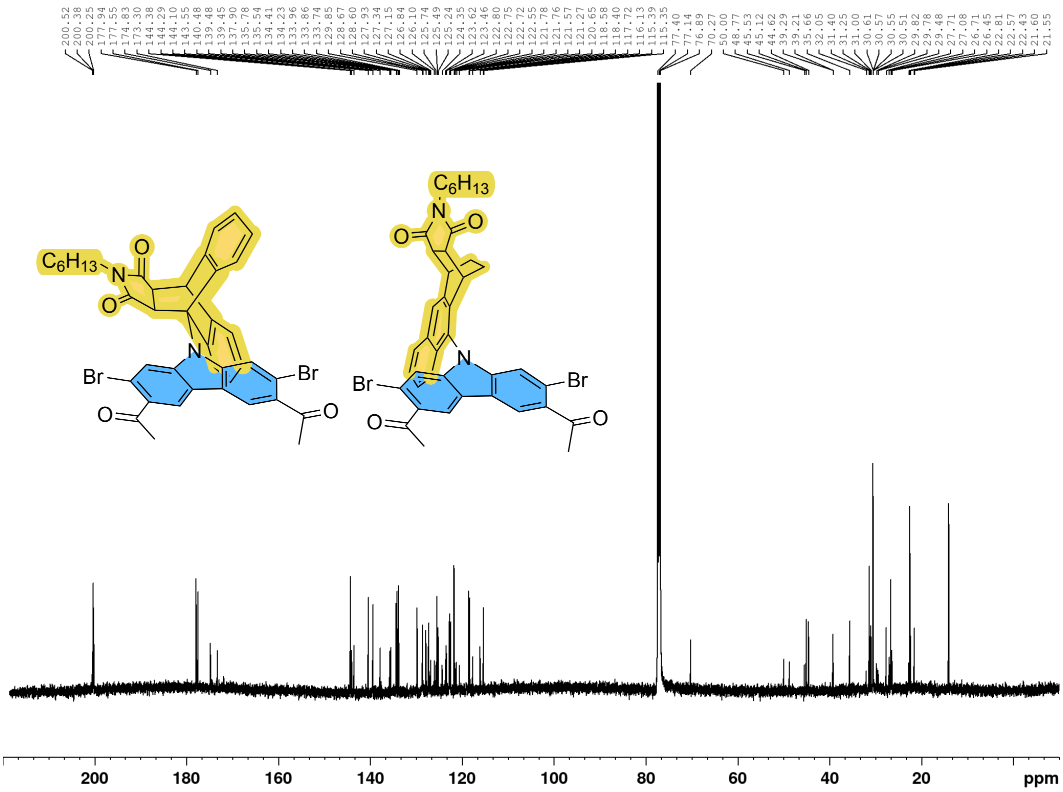

Figure S14. ^{13}C NMR (125 MHz, $CDCl_3$, 25 ºC) spectrum of mixture **S2b** and **S3b**.

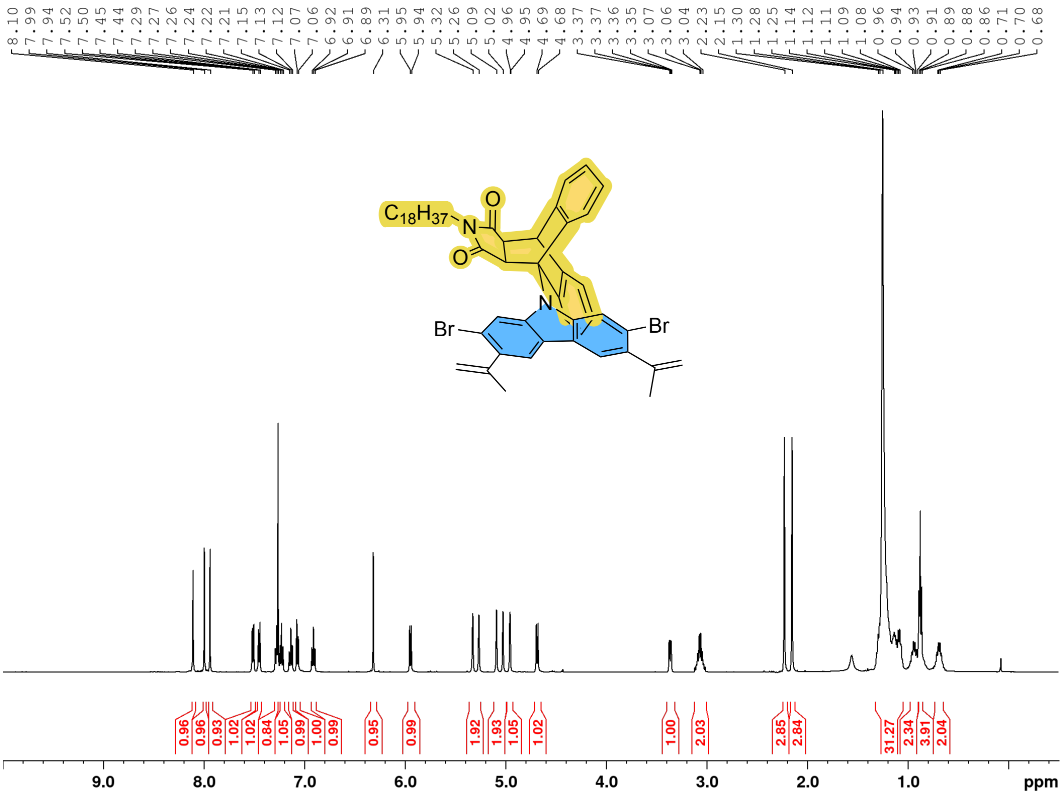

Figure S15. ^{1}H NMR (500 MHz, $CDCl_3$, 25 ºC) spectrum of **4a**.

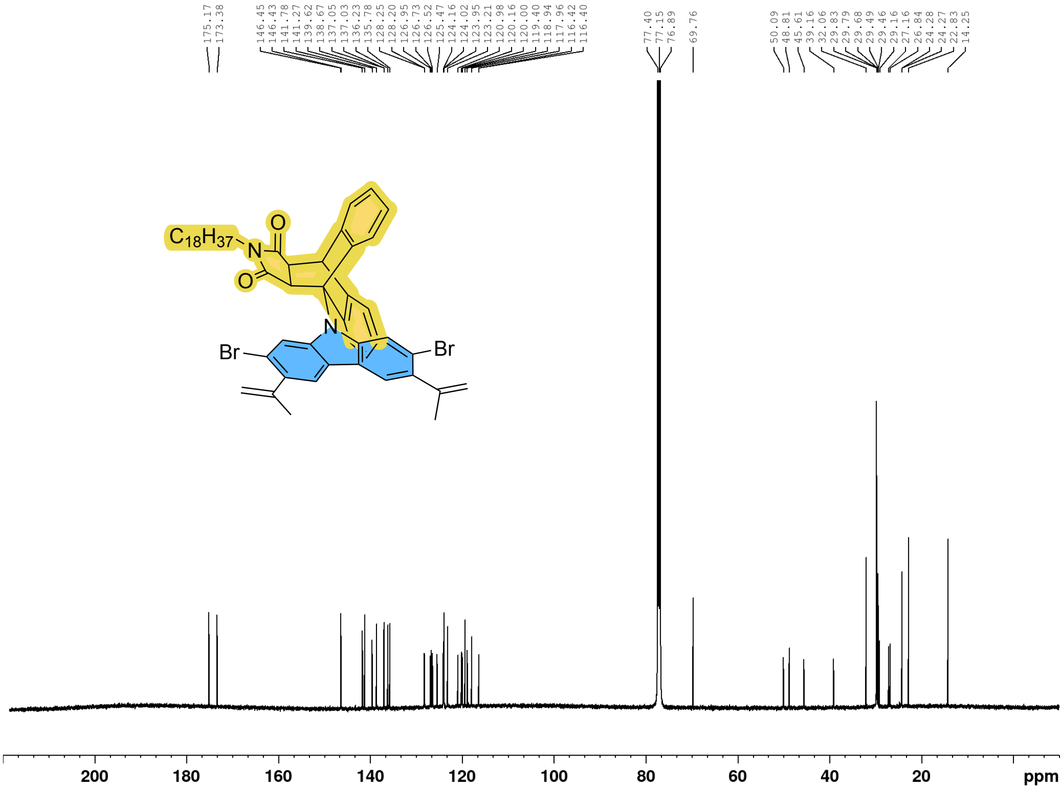

Figure S16. ^{13}C NMR (125 MHz, $CDCl_3$, 25 ºC) spectrum of **4a**.

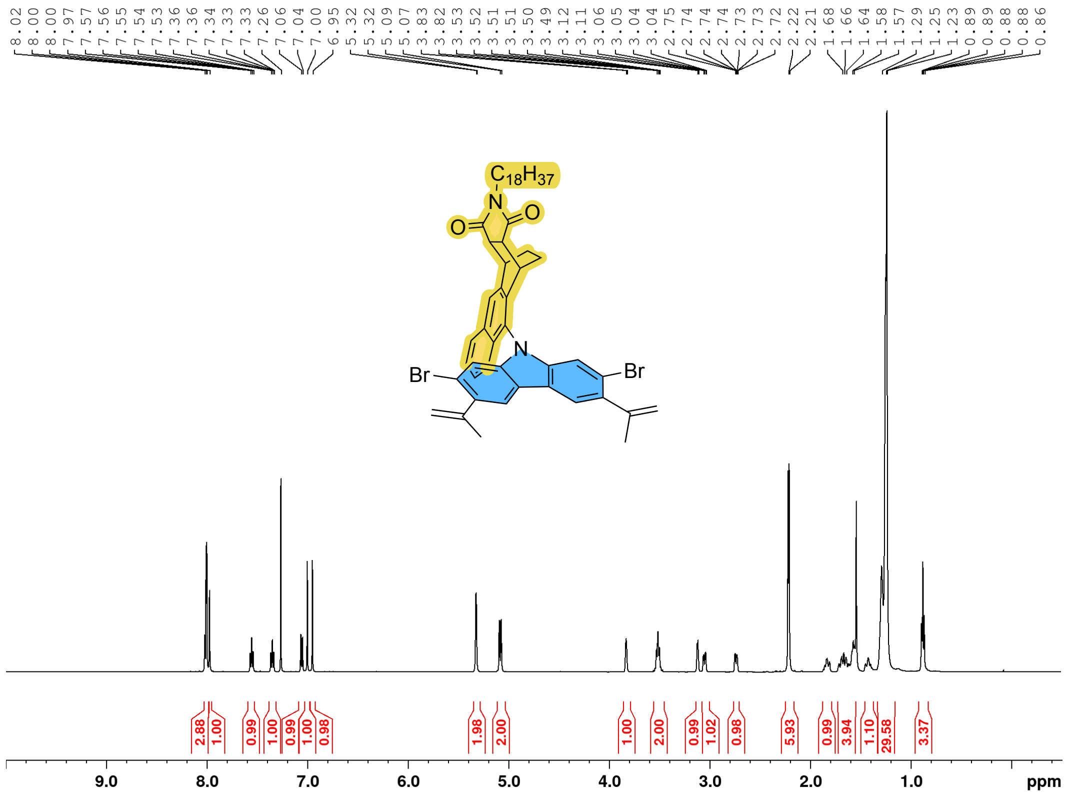

Figure S17. ^{1}H NMR (500 MHz, CDCl$_3$, 25 ºC) spectrum of **5a**.

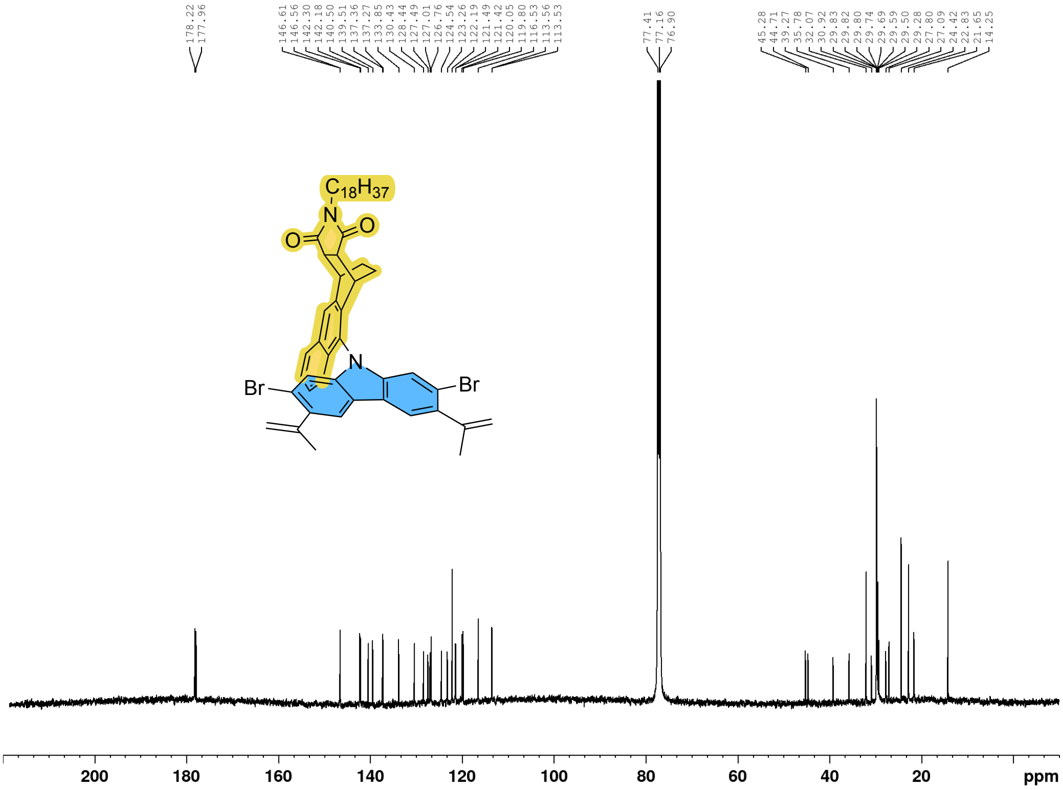

Figure S18. ^{13}C NMR (125 MHz, CDCl$_3$, 25 ºC) spectrum of **5a**.

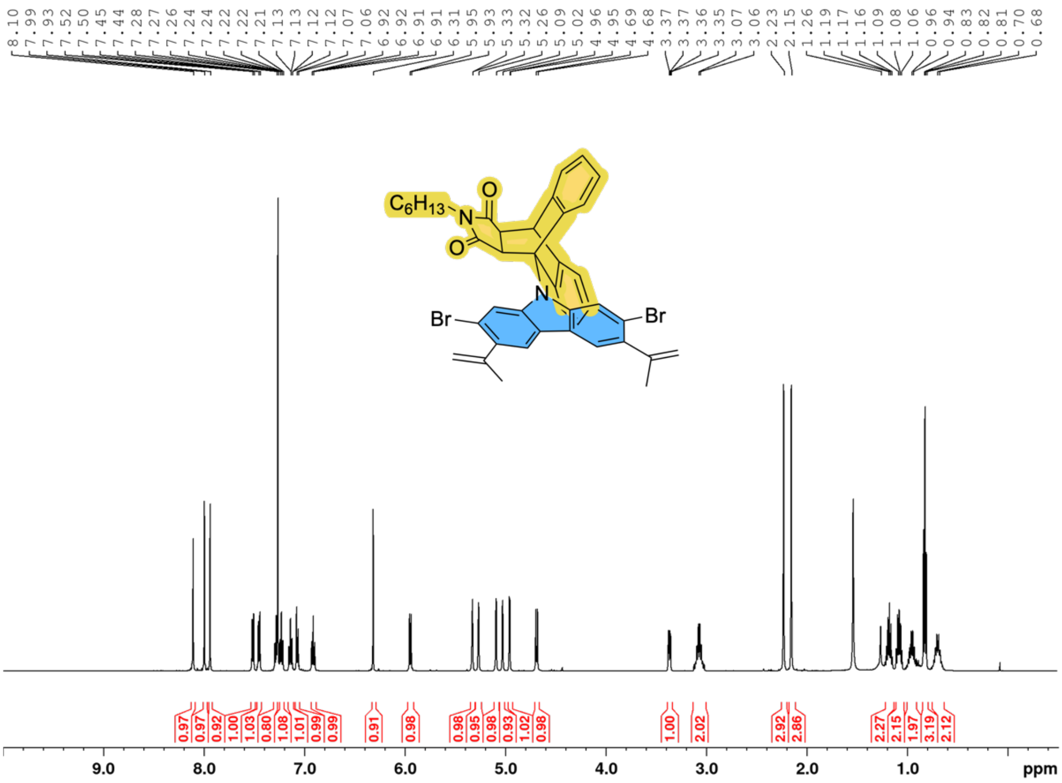

Figure S19. ^{1}H NMR (500 MHz, $CDCl_3$, 25 ºC) spectrum of **4b**.

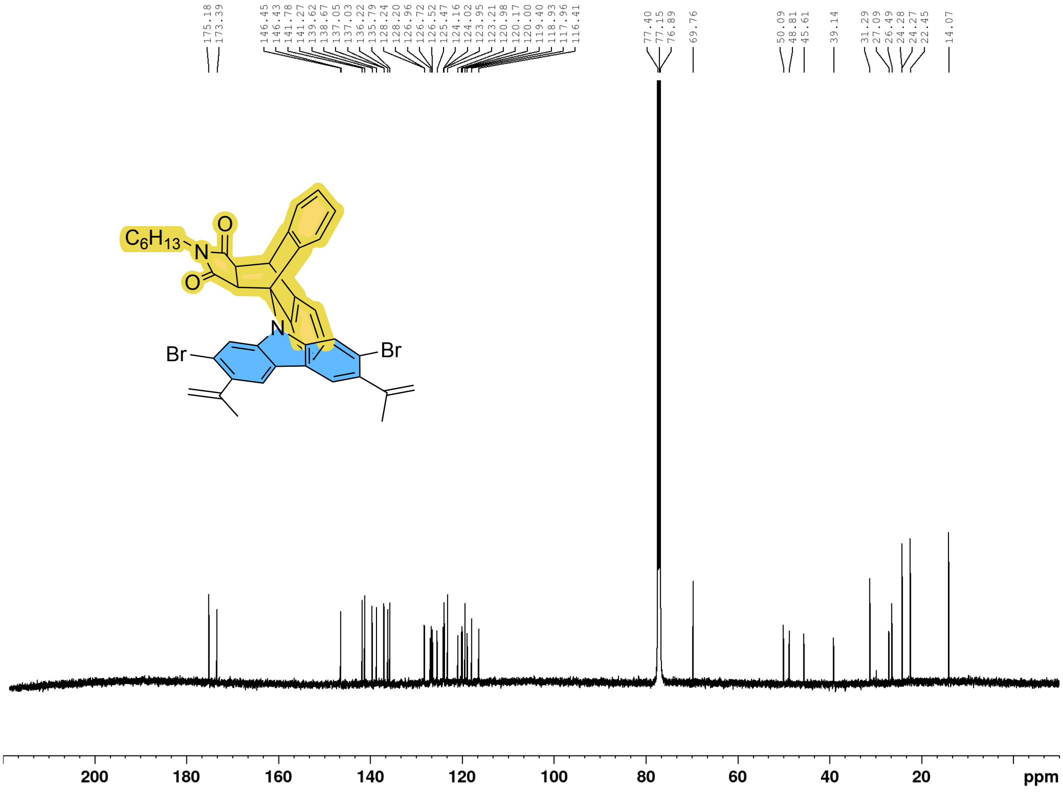

Figure S20. ^{13}C NMR (125 MHz, $CDCl_3$, 25 ºC) spectrum of **4b**.

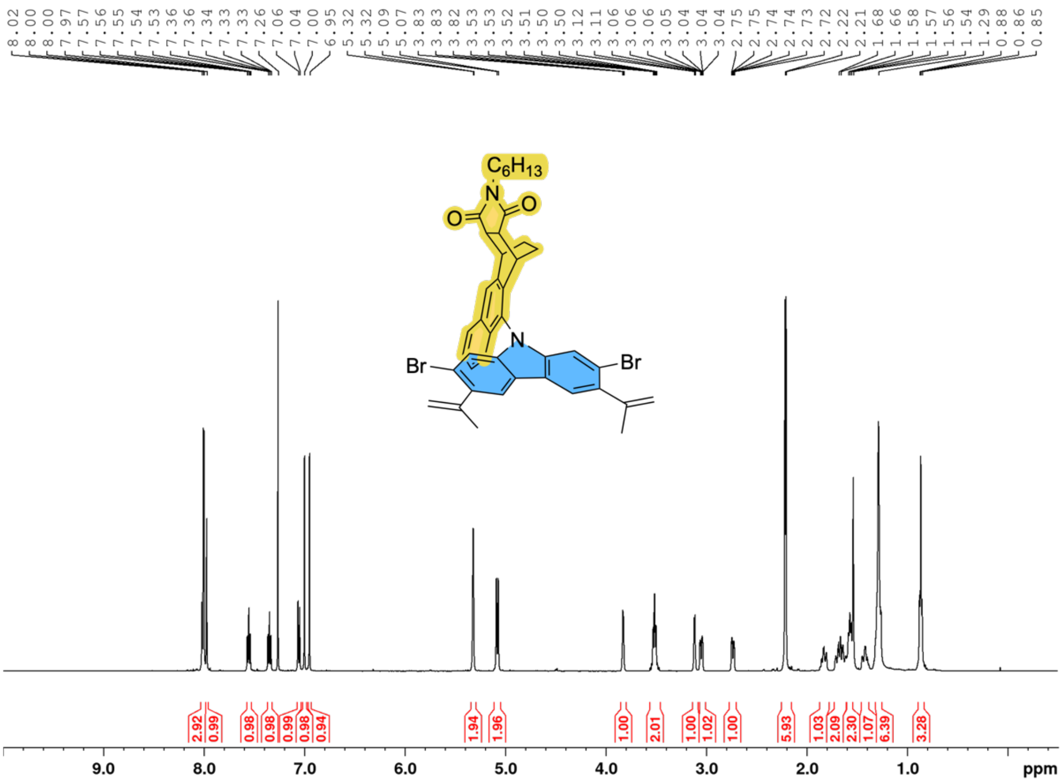

Figure S21. ^{1}H NMR (500 MHz, $CDCl_3$, 25 ºC) spectrum of **5b**.

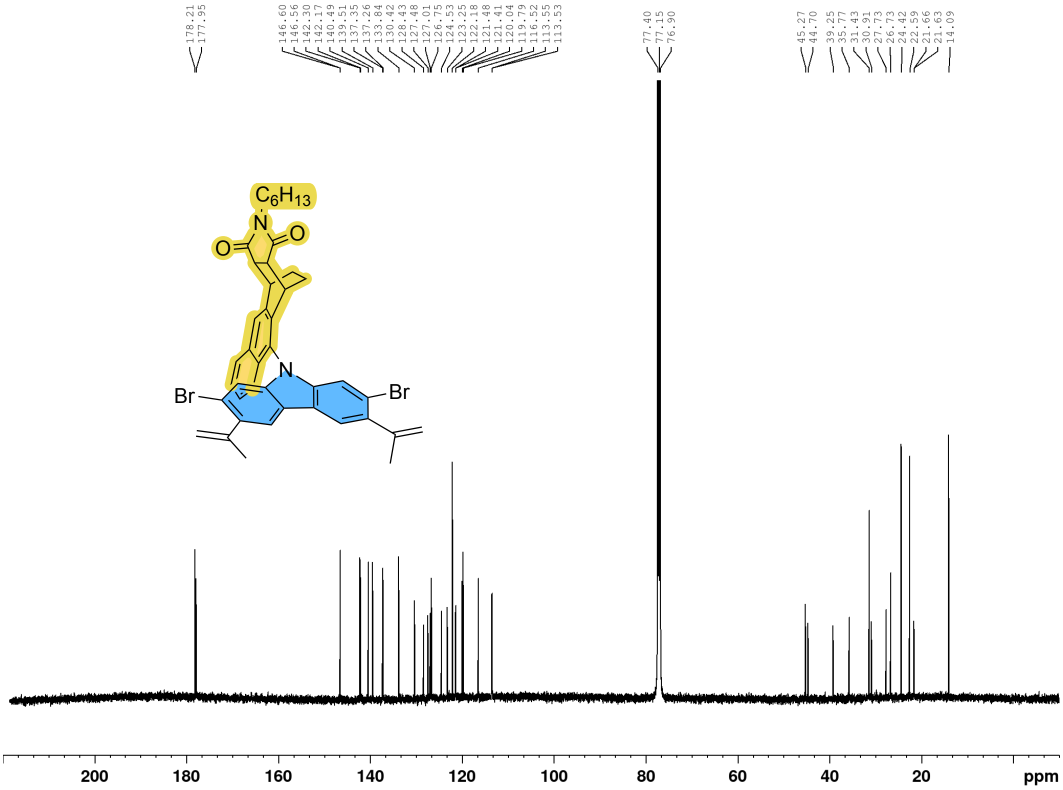

Figure S22. ^{13}C NMR (125 MHz, $CDCl_3$, 25 ºC) spectrum of **5b**.

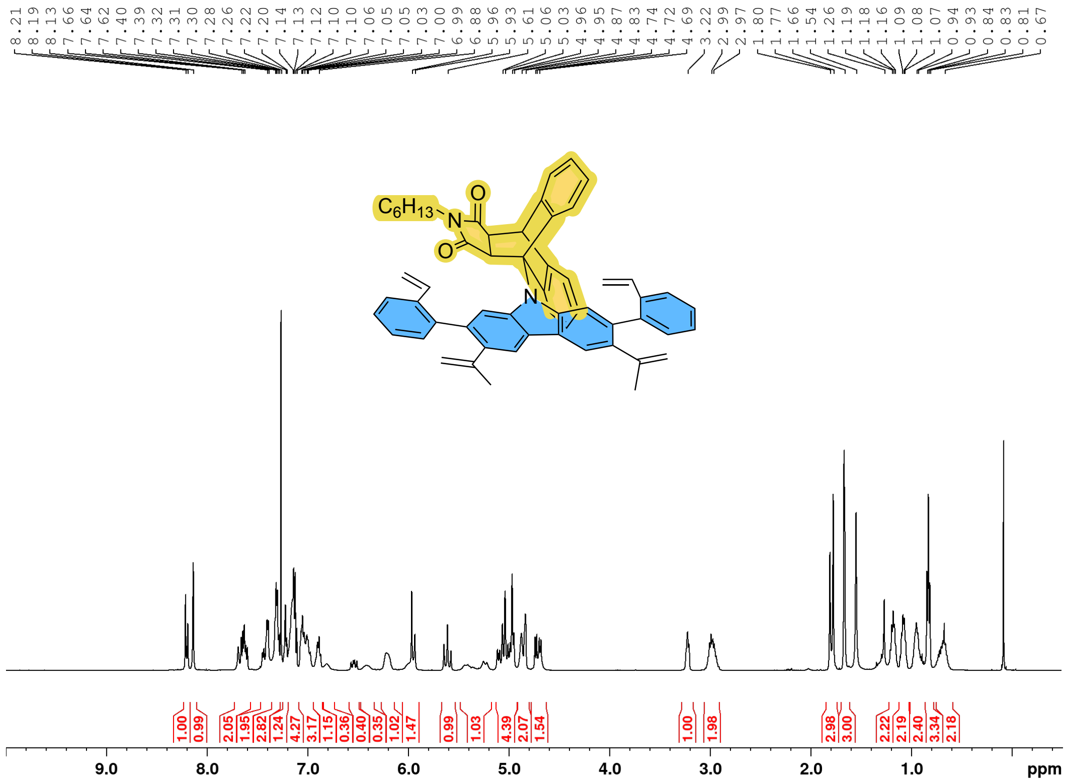

Figure S23. ^{1}H NMR (500 MHz, CDCl$_3$, 25 ºC) spectrum of **6**.

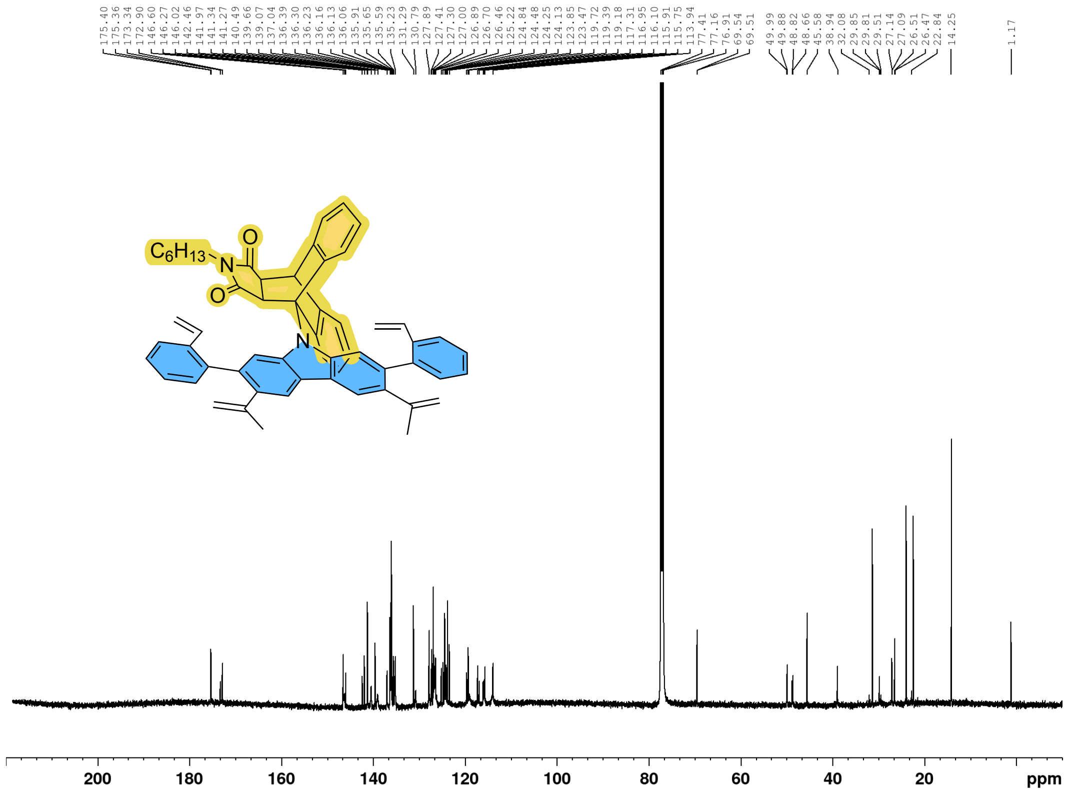

Figure S24. ^{13}C NMR (125 MHz, CDCl$_3$, 25 ºC) spectrum of **6**.

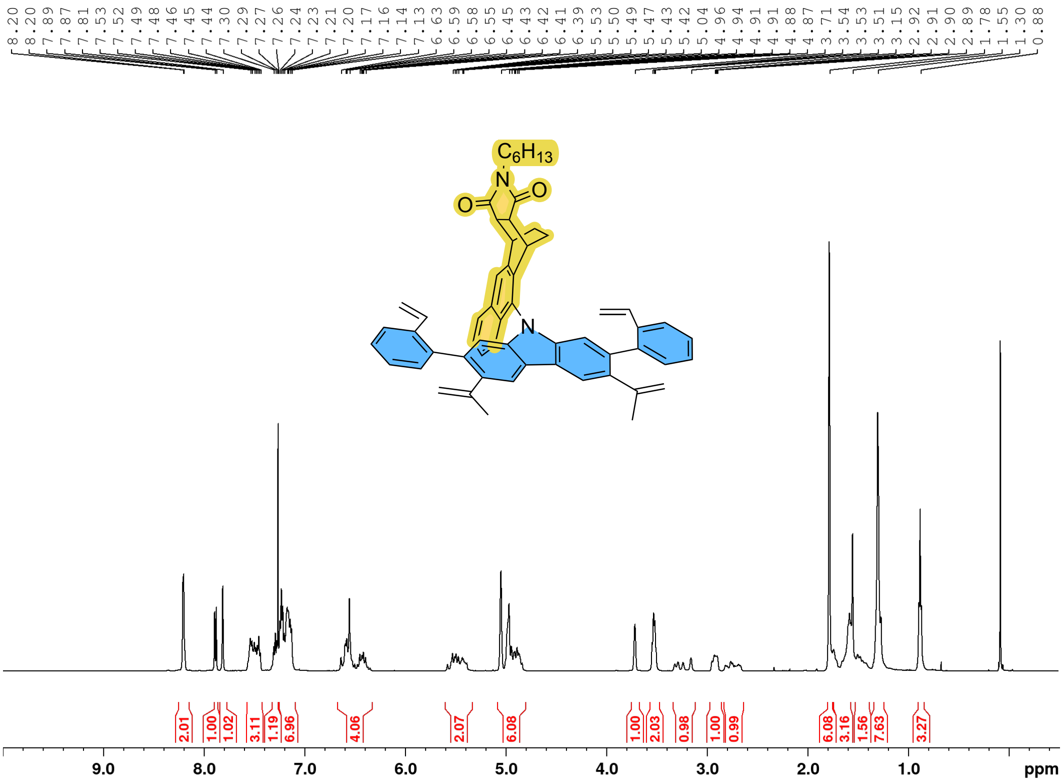

Figure S25. ^{1}H NMR (500 MHz, $CDCl_3$, 25 ºC) spectrum of **8**.

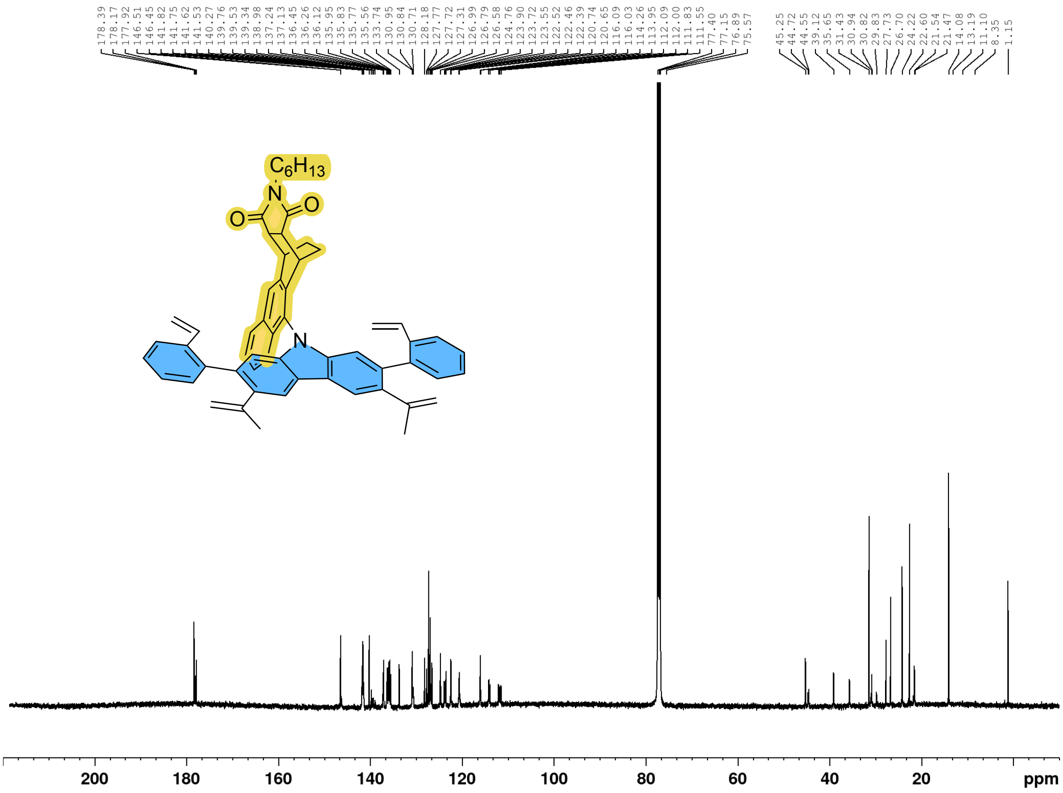

Figure S26. ^{13}C NMR (125 MHz, $CDCl_3$, 25 ºC) spectrum of **8**.

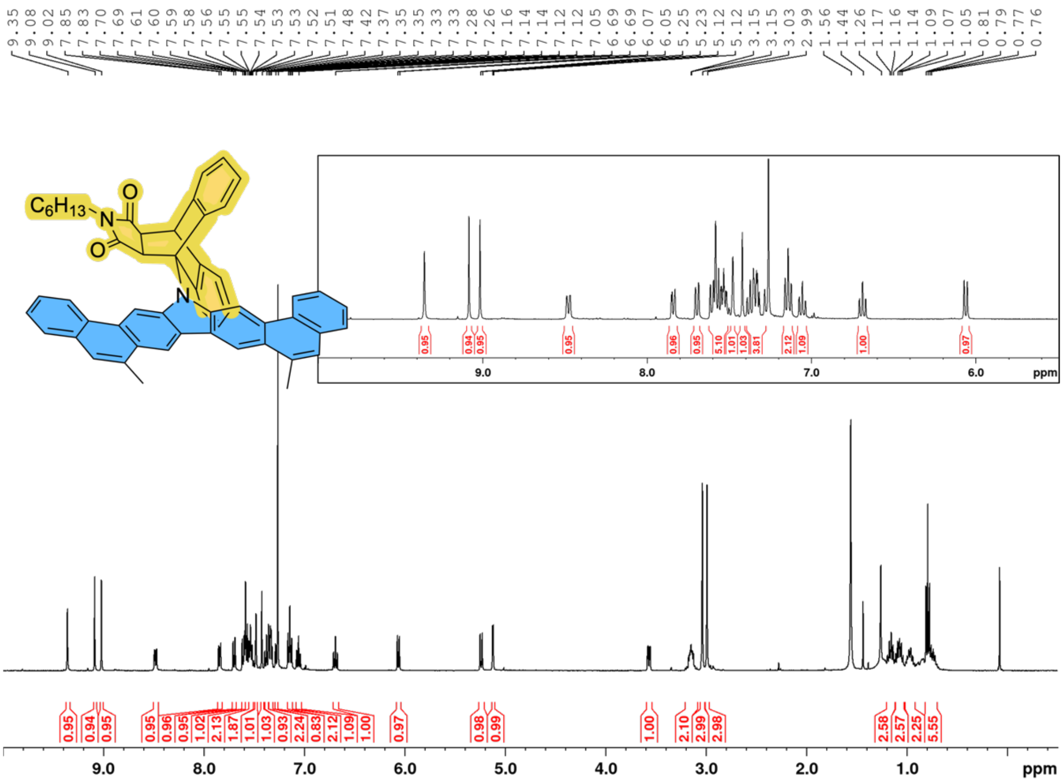

Figure S27. ^{1}H NMR (500 MHz, $CDCl_3$, 25 ºC) spectrum of **7**.

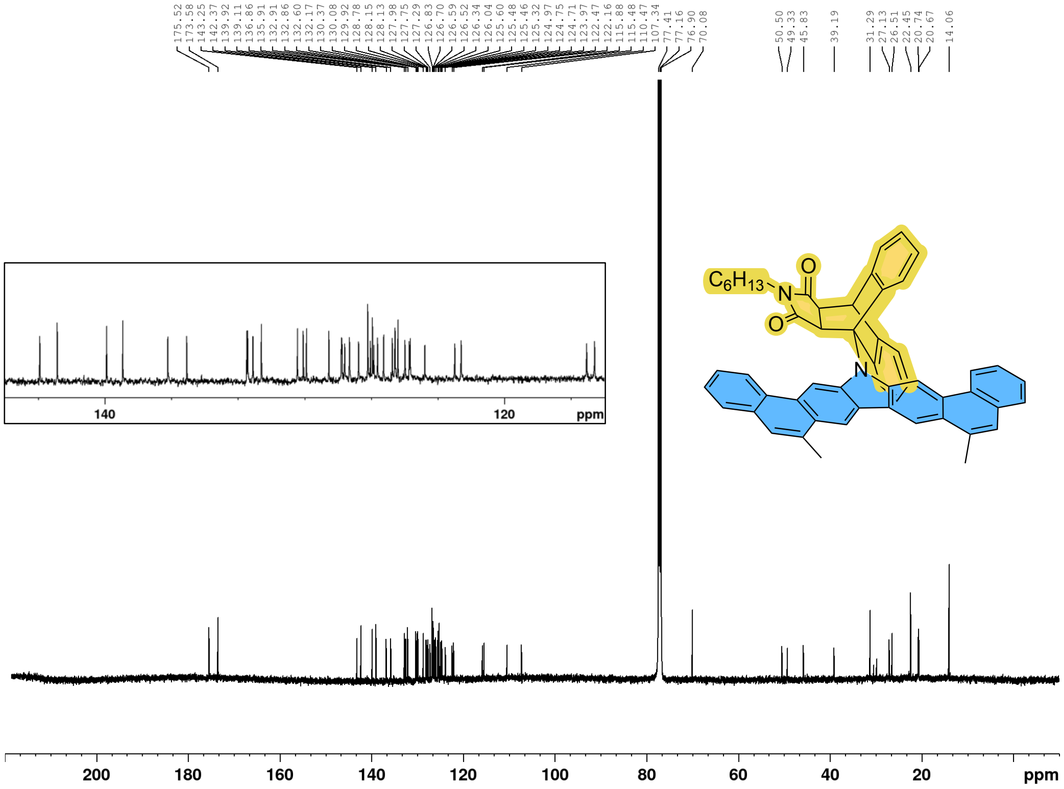

Figure S28. ^{13}C NMR (125 MHz, $CDCl_3$, 25 ºC) spectrum of **7**.

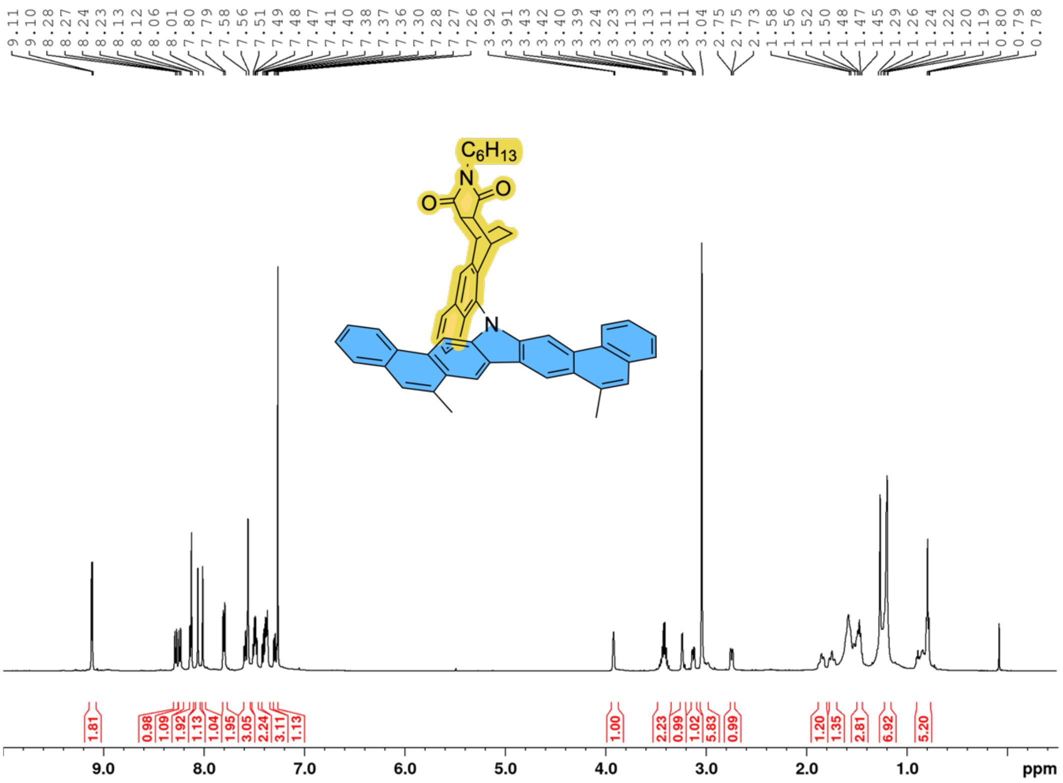

Figure S29. ^{1}H NMR (500 MHz, $CDCl_3$, 25 ºC) spectrum of **9**.

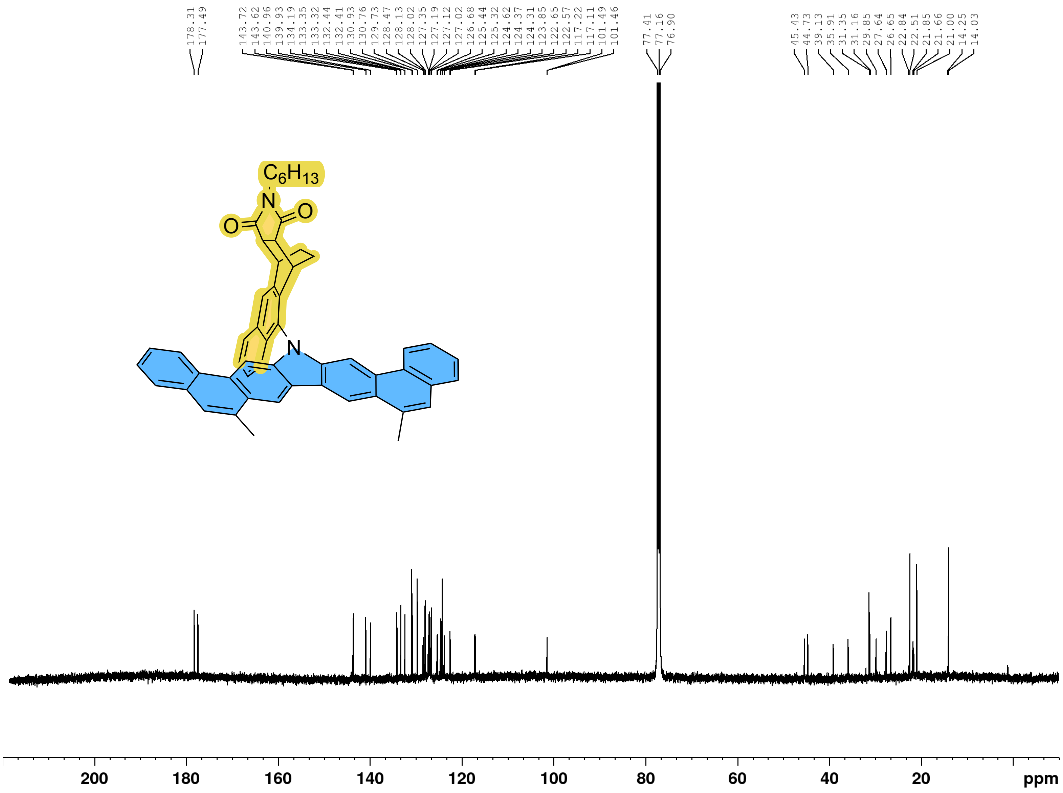

Figure S30. ^{13}C NMR (125 MHz, $CDCl_3$, 25 ºC) spectrum of **9**.

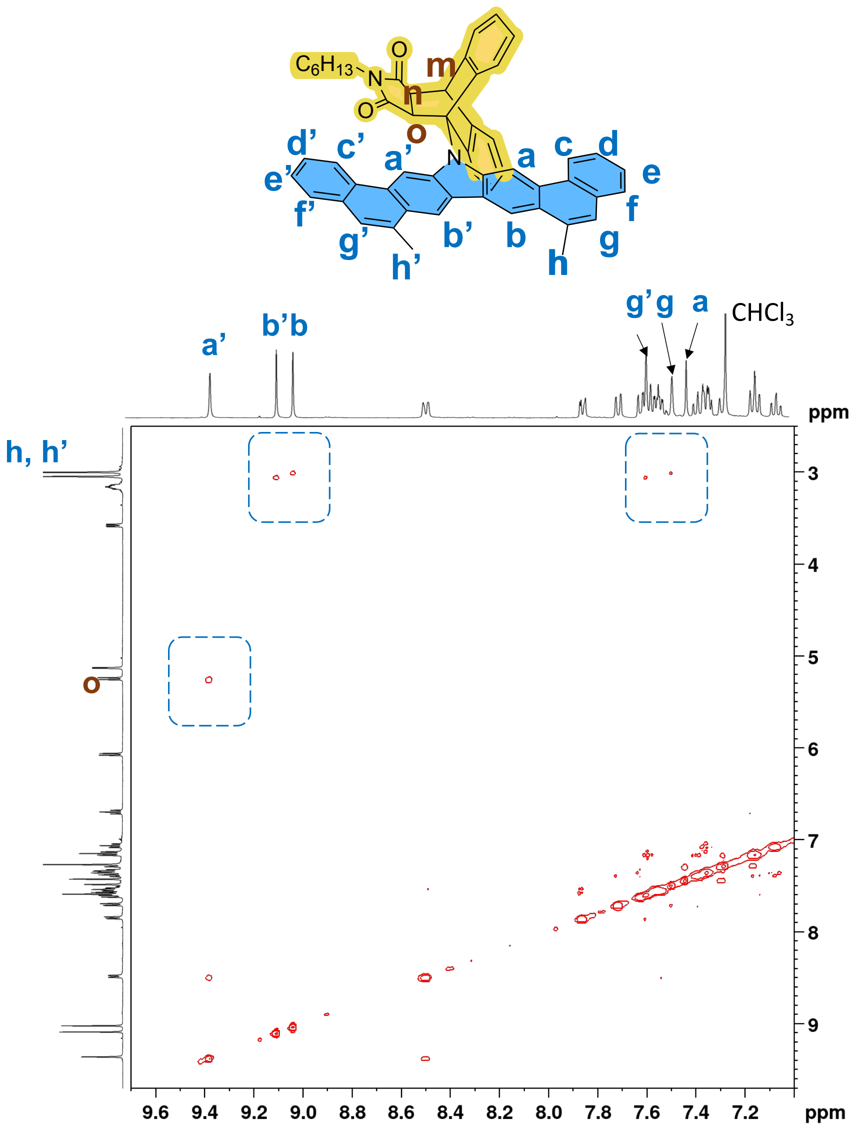

Figure S31. Partial ^1H-^1H NOESY (500 MHz, $CDCl_3$, 25 °C) spectrum of **7**. Nuclear Overhauser effect of backbone proton (a') and AMA proton (o) was observed. Their spatial proximity is also supported by the structure of single crystal.

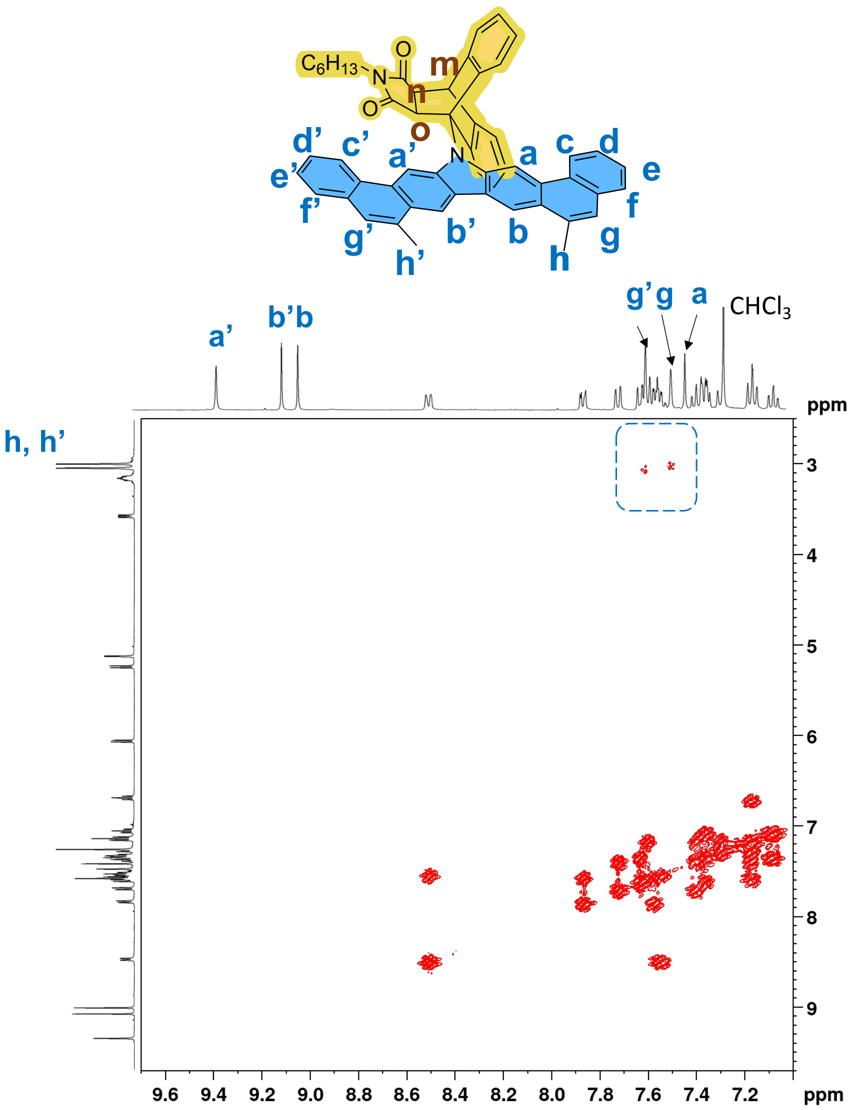

Figure S32. Partial ^{1}H-^{1}H COSY (500 MHz, $CDCl_3$, 25 ºC) spectrum of **7**.

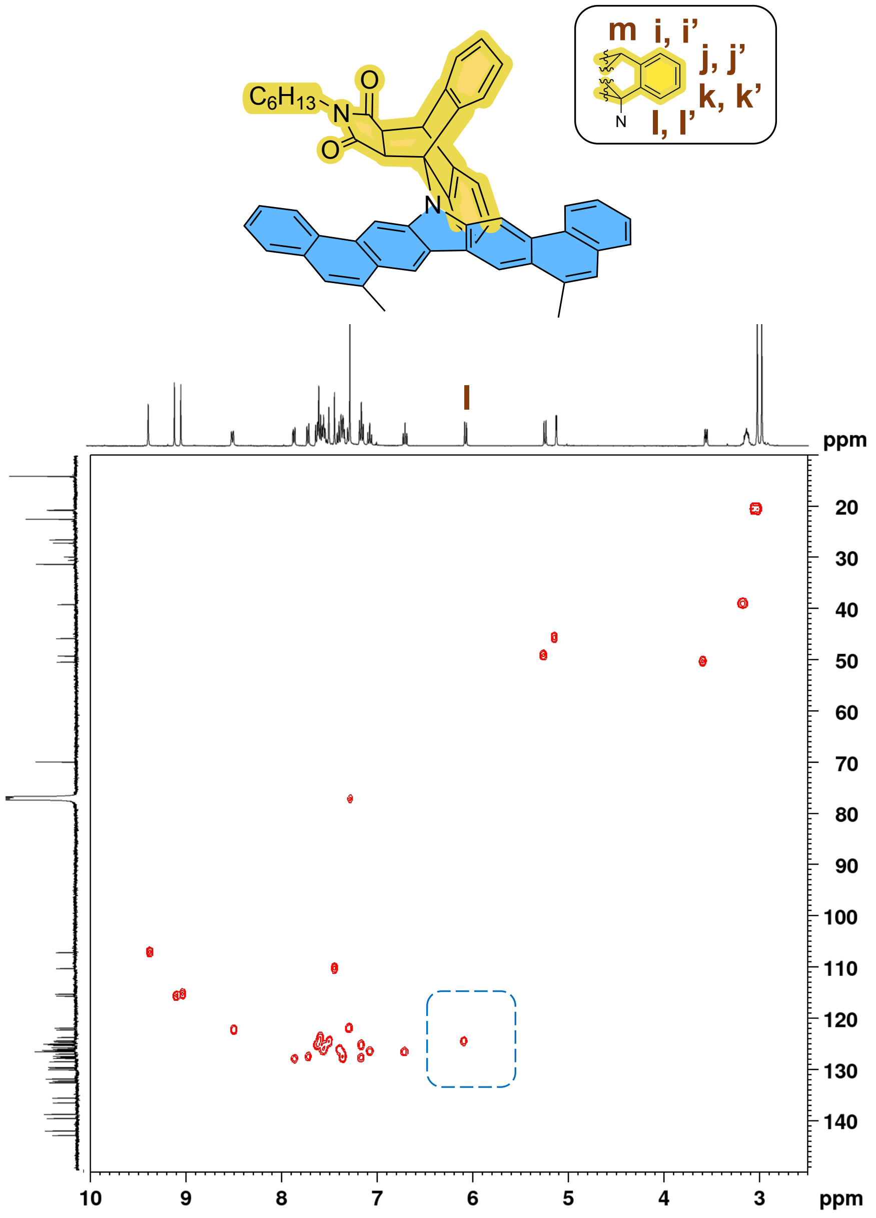

Figure S33. Partial ^{1}H-^{13}C HSQC (500 MHz, $CDCl_3$, 25 ºC) spectrum of **7**. Proton (l) on AMA is highly shielded by carbazole backbone such that it has an unusual upfield-shifted chemical shift of 6.06 ppm.

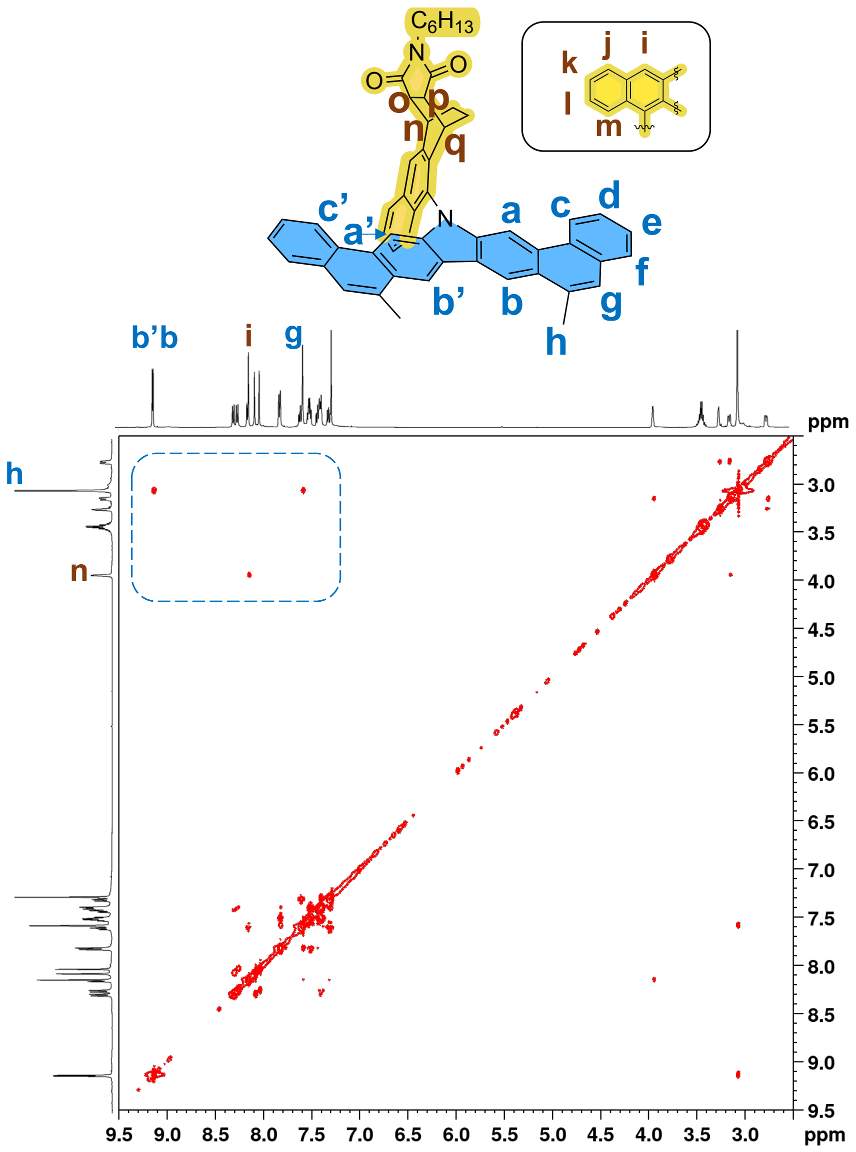

Figure S34. Partial ^{1}H-^{1}H NOESY (500 MHz, $CDCl_3$, 25 ºC) spectrum of **9**.

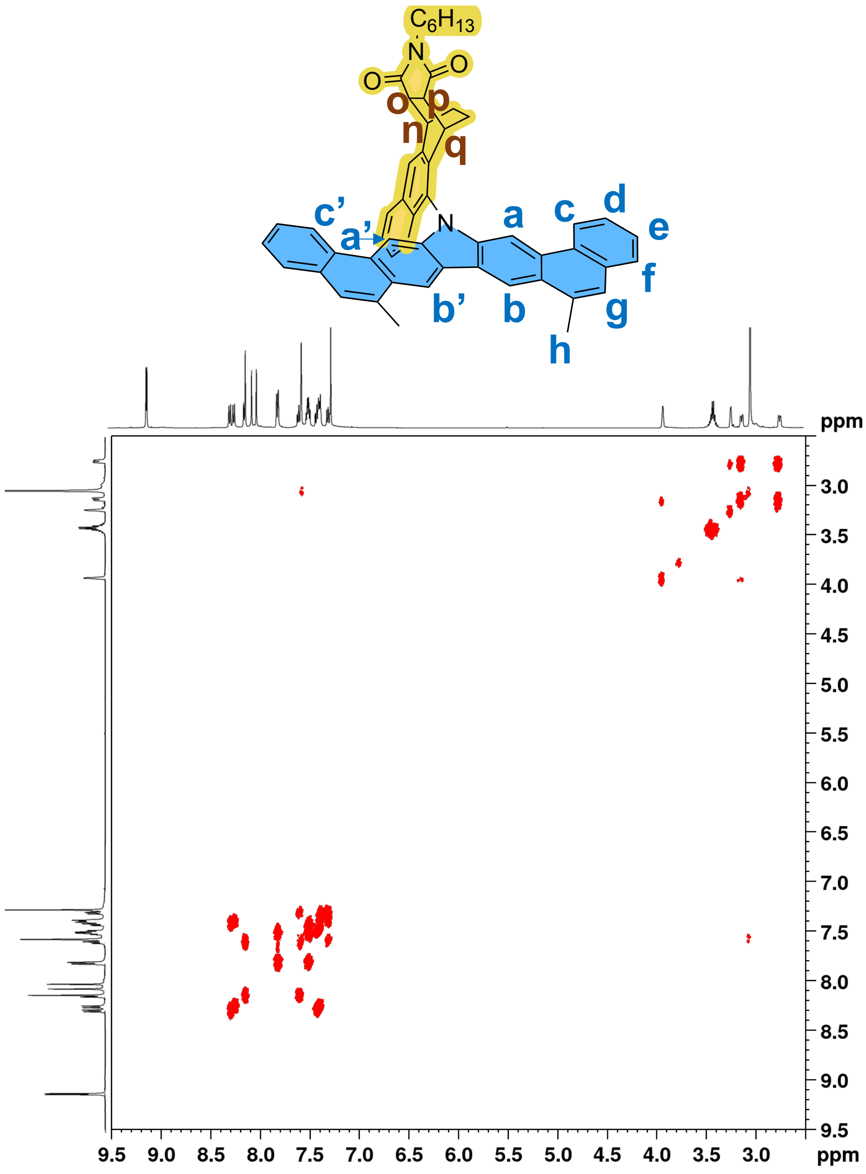

Figure S35. Partial ¹H-¹H COSY (500 MHz, $CDCl_3$, 25 ºC) spectrum of **9**.

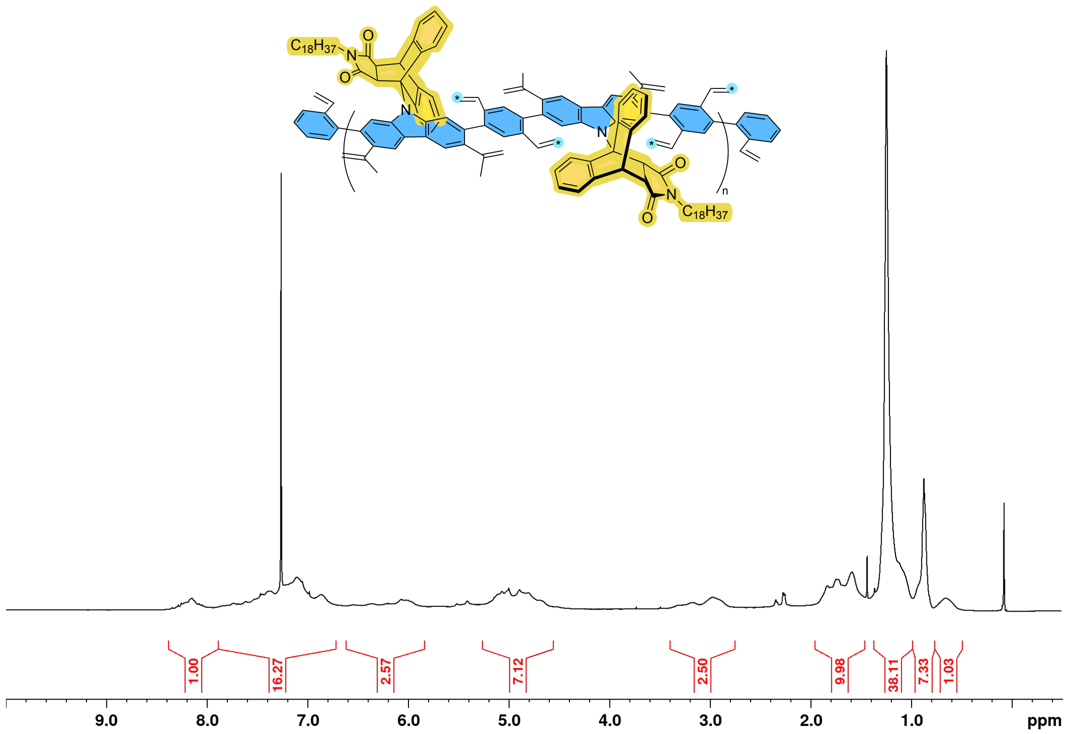

Figure S36. ^{1}H NMR (500 MHz, $CDCl_3$, 25 ºC) spectrum of **P1**.

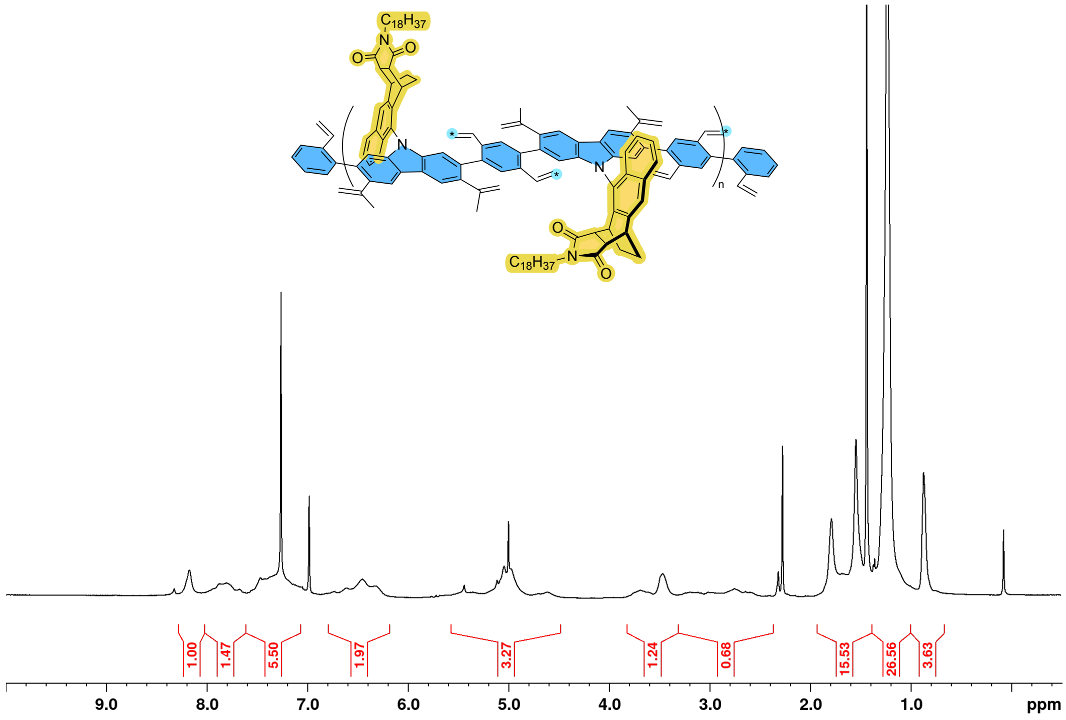

Figure S37. ^{1}H NMR (500 MHz, $CDCl_3$, 25 ºC) spectrum of **P2**.

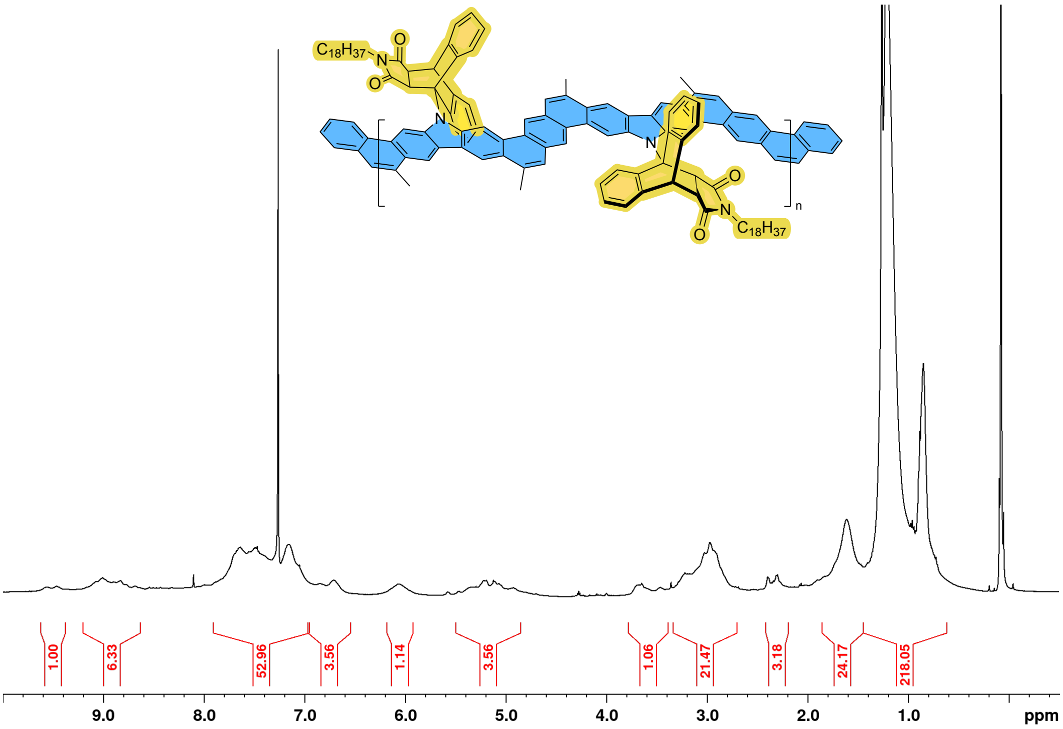

Figure S38. ^{1}H NMR (500 MHz, $CDCl_3$, 25 ºC) spectrum of **LP1**.

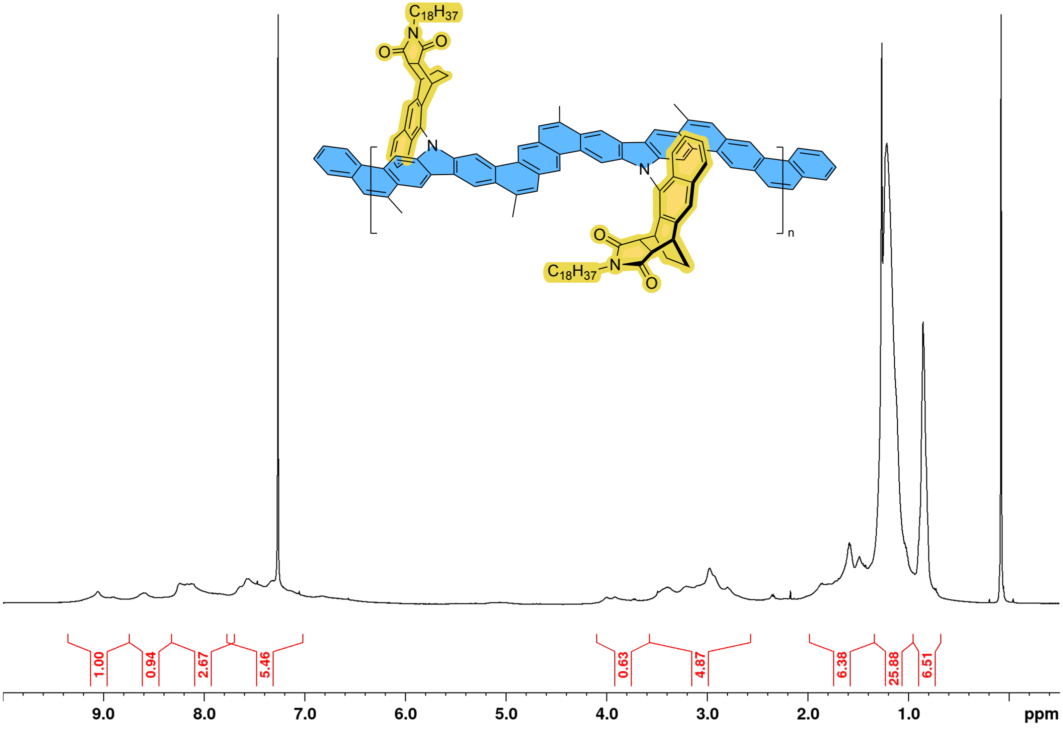

Figure S39. ^{1}H NMR (500 MHz, $CDCl_3$, 25 ºC) spectrum of **LP2**.

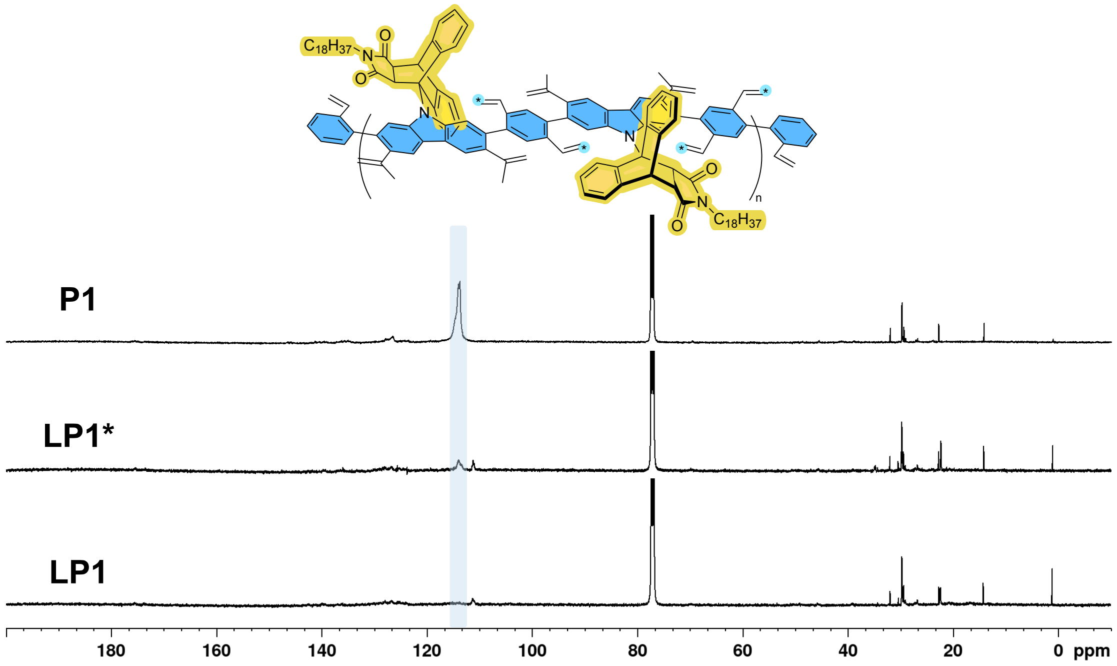

Figure S40. ^{13}C NMR (125 MHz, $CDCl_3$, 25 ºC) spectrum of **P1**, **LP1*** (not fully cyclized), and defect-free **LP1**. Resonance signals of ^{13}C-enriched vinyl groups are highlighted in blue.

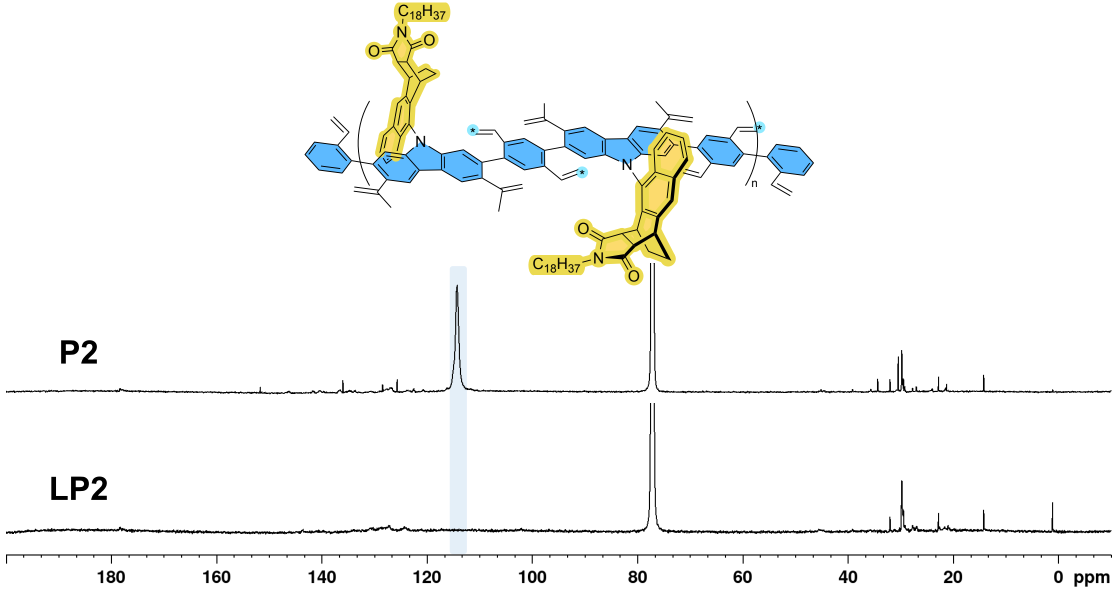

Figure S41. ^{13}C NMR (125 MHz, $CDCl_3$, 25 ºC) spectrum of **P2** and defect-free **LP2**. Resonance signals of ^{13}C-enriched vinyl groups are highlighted in blue.

4. Single-Crystal X-ray Diffraction

Single crystals of **7** and **9** were grown by slow evaporation technique from the respective solutions with mixed solvents of chloroform and acetonitrile at room temperature. The associated CIF files are available through The Cambridge Crystallographic Data Centre via www.ccdc.cam.ac.uk/data_request/cif. (**7**: CCDC-2456283; **9**: CCDC-2456284)

A Leica M80 microscope was used to identify suitable single crystals of **7** and **9** showing well defined faces from representative samples of crystals of the same habit. The crystals mounted on a nylon loop were then placed in a cold nitrogen stream (Oxford) maintained at T= 110.00 K. Crystal screening, unit cell determination, and data collection were carried out using a Bruker Venture (PHOTON III) diffractometer. The diffraction patterns were indexed and the total number of runs and images were based on the strategy calculation from the program APEX 3 (Bruker, 2018) for **7** and from CrysAlisPro 1.171.42.63a (Rigaku OD, 2022) for **9**. Data were measured using ϕ and ω scans with CuK_{α} radiation. The maximum resolutions that were achieved were Θ= 70.099° (0.82 Å) for **7** and Θ= 72.838° (0.81 Å) for **9**. The unit cells were refined using SAINT V8.38A (Bruker, 2018) on 9654 reflections, 22 % of the observed reflections for **7**, and SAINT V8.40B on 9921 reflections, 13% of the observed reflections for **9**.

Integrated intensity information for each reflection was obtained by reduction of data frames using SAINT V8.38A (Bruker, 2018) for **7** and SAINT V8.40B (?, 2016) for **9**. The final completeness is 99.70 % out to 70.099° in Θ for **7** and 100.00 % out to 72.838° in Θ for **9**. SADABS-2016/2 (Bruker, 2016/2) was used for absorption correction. wR2(int) was 0.1242 before and 0.0663 after correction for **7** and was 0.1474 before and 0.0951 after correction for **9**. The ratio of minimum to maximum transmission is

0.8287 for **7** and 0.7793 for **9**. The $\lambda/2$ correction factor is not present for both samples. The absorption coefficient μ of **7** is 0.603 mm^{-1} at this wavelength (λ= 1.54178Å) and the minimum and maximum transmissions are 0.485 and 0.586. The absorption coefficient μ of **9** is 0.506 mm^{-1} at this wavelength (λ= 1.54178Å) and the minimum and maximum transmissions are 0.587 and 0.754.

Systematic reflection conditions and statistical tests of the data suggested the space group $P2_1/n$ (# 14) for **7** and $P2_1/c$ (# 14) for **9** and were confirmed by ShelXT (Sheldrick, 2015) structure solution program using dual methods. The structures were refined by full matrix least squares minimization on $\boldsymbol{F^2}$ using version 2018/3 of XL (Sheldrick, 2008). All non-hydrogen atoms were refined anisotropically. Hydrogen atom positions were calculated geometrically and refined using the riding model. For **7**, elongated thermal ellipsoids on C3s suggested disordered solvent, which was modeled between two positions with an occupancy ratio of 0.31. Appropriate restraints and constraints were added to keep the bond distances, angles, and thermal ellipsoids meaningful.

For each sample, there is a single molecule in the asymmetric unit, which is represented by the reported sum formula. In other words: Z is 4 and Z' is 1.

Table S3. Summarized crystallographic data for **7** and **9**.

**Compound**	**7**	**9**
Formula	$C_{56}H_{47}N_3O_2$	$C_{54}H_{46}N_2O_2$
$D_{calc.}$/ g cm^{-3}	1.284	1.087
μ/mm^{-1}	0.603	0.506
Formula weight	793.96	754.93
Color	orange	orange
Shape	block-shaped	needle-shaped
Size/mm^3	0.51×0.26×0.15	0.10×0.01×0.01
T/K	110.00	110.00
Crystal System	monoclinic	monoclinic
Space Group	$P2_1/n$	$P2_1/c$
a/Å	8.5619(2)	12.6618
b/Å	18.4170(6)	23.4420
c/Å	26.1120(8)	15.7197
α/°	90	90
β/°	93.8110(10)	98.519
γ/°	90	90
V/Å^3	4108.4(2)	4614.4
Z	4	4
Z'	1	1
Wavelength/Å	1.54178	1.54178
Radiation type	Cu K_α	Cu K_α
Θ_{min}/°	2.938	3.411
Θ_{max}/°	70.099	72.838
Measured Refl's.	44765	73509
Indep't Refl's	7800	9132
Refl's I≥2 σ(I)	7264	5557
R_{int}	0.0416	0.0922
Parameters	577	527
Restraints	87	0
Largest Peak	0.284	0.715
Deepest Hole	-0.288	-0.396
GooF	1.030	1.053
wR_2 (all data)	0.1045	0.3106
wR_2	0.1025	0.2695
R_1 (all data)	0.0429	0.1343
R_1	0.0403	0.0950

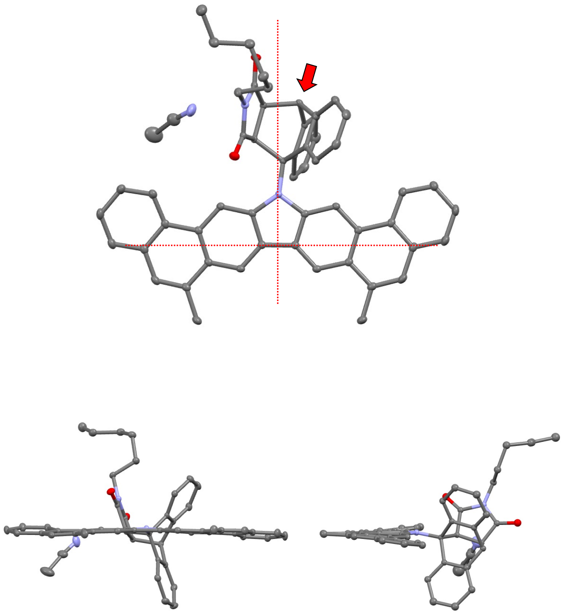

Figure S42. Thermal ellipsoids plot (50 % probability) of **7** in top and side view. Hydrogen atoms and selected atoms from solvent (acetonitrile) are omitted for clarity. The bridge head carbon of 9,10-AMA (red arrow) is distorted from the central axis of carbazole.

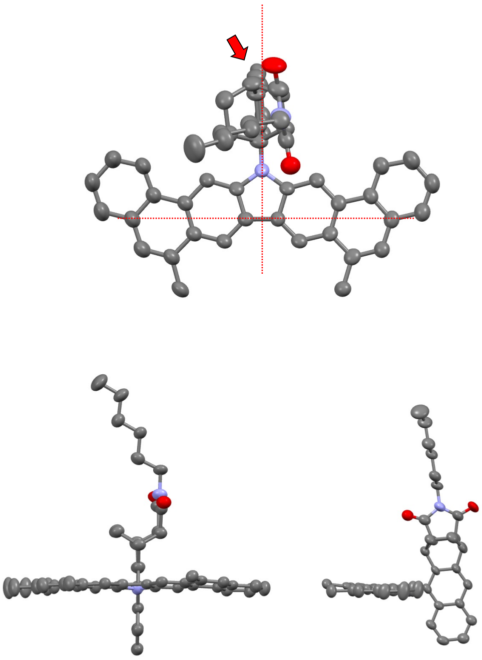

Figure S43. Thermal ellipsoids plot (50 % probability) of **9** in top and side view. Hydrogen atoms are omitted for clarity. The naphthalene moiety of 1,4-AMA (red arrow) does not distort and remains close to the central axis of carbazole.

5. Determination of Restricted Rotation

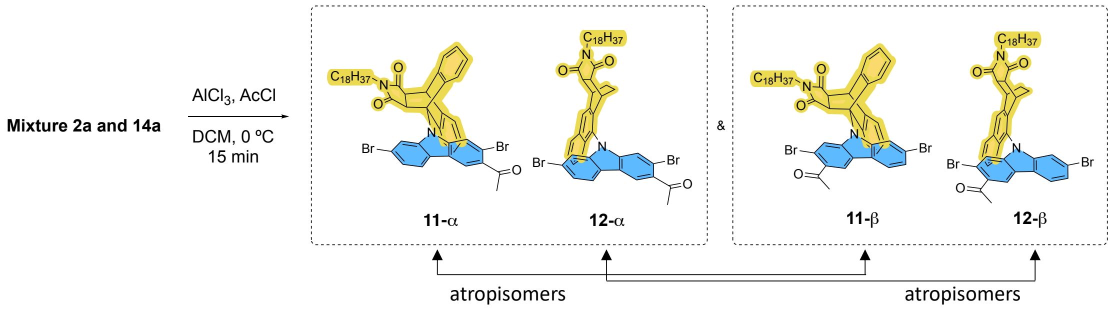

Scheme S2. Synthesis of mono-acylated atropisomers

To assess the bond rotation, mono-acylated **11** and **12** were synthesized through the following procedures. $AlCl_3$ (200 mg, 1.5 mmol) was suspended in anhydrous DCM (3 ml) at 0 ºC, to which acetyl chloride (105 μl, 1.5 mmol) was added dropwise. After stirring for 5 min, a mixture of **2a** and **S1a** (250 mg, 0.3 mmol) in DCM (3 ml) was added dropwise. The reaction mixture was stirred at 0 ºC for 15 min before quenched by 2 M $HCl_{(aq)}$ in an ice bath. The organic layer was further washed by 2 M $HCl_{(aq)}$, H_2O, and saturated $NH_4Cl_{(aq)}$ and dried over $MgSO_4$. After removing solvents under reduced pressure, the residue was purified through silica column chromatography (hexane/DCM: 1/1 to 1/9) to afford the first inseparable mixture **11-α** and **12-α** as a white solid (90 mg, 35% yield), and the second inseparable mixture **11-β** and **12-β** as a white solid (130 mg, 50% yield).

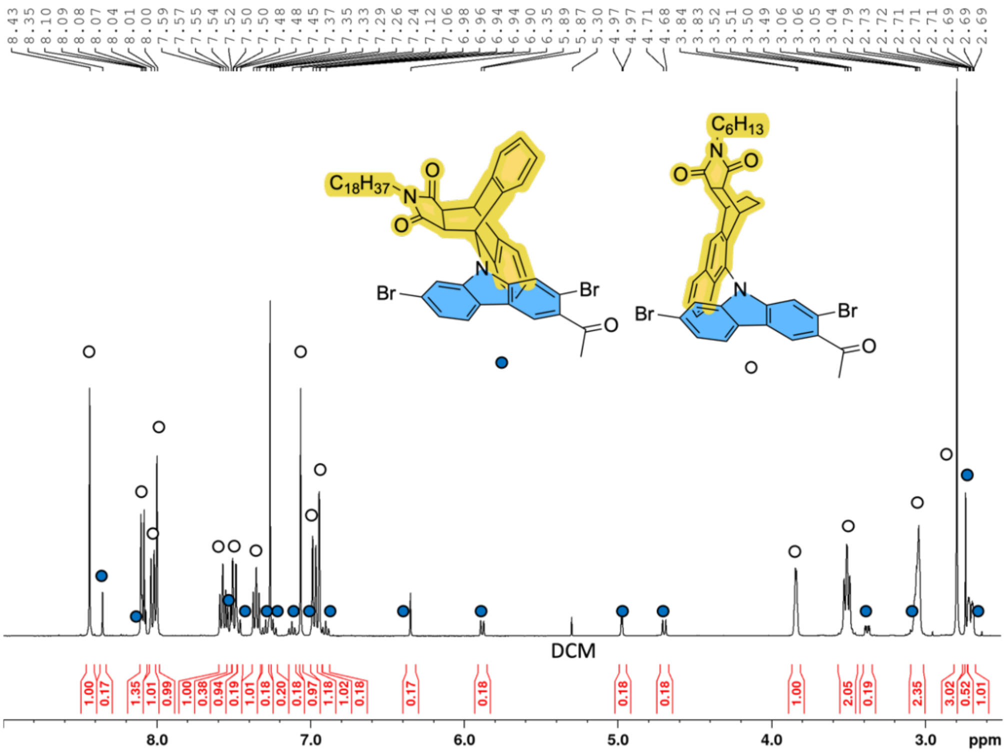

Figure S44. Partial ^{1}H NMR (400 MHz, $CDCl_3$, 25 ºC) spectrum of mixture **11-α** (blue circle) and **12-α** (white circle).

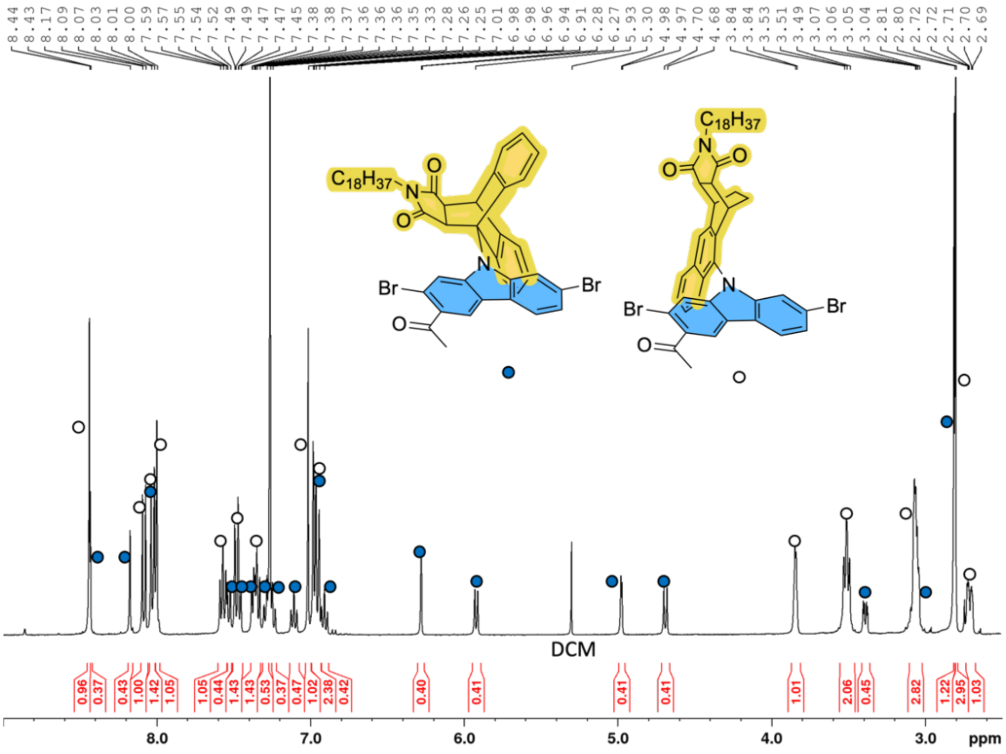

Figure S45. Partial ^{1}H NMR (400 MHz, $CDCl_3$, 25 ºC) spectrum of **11-β** (blue circle) and **12-β** (white circle).

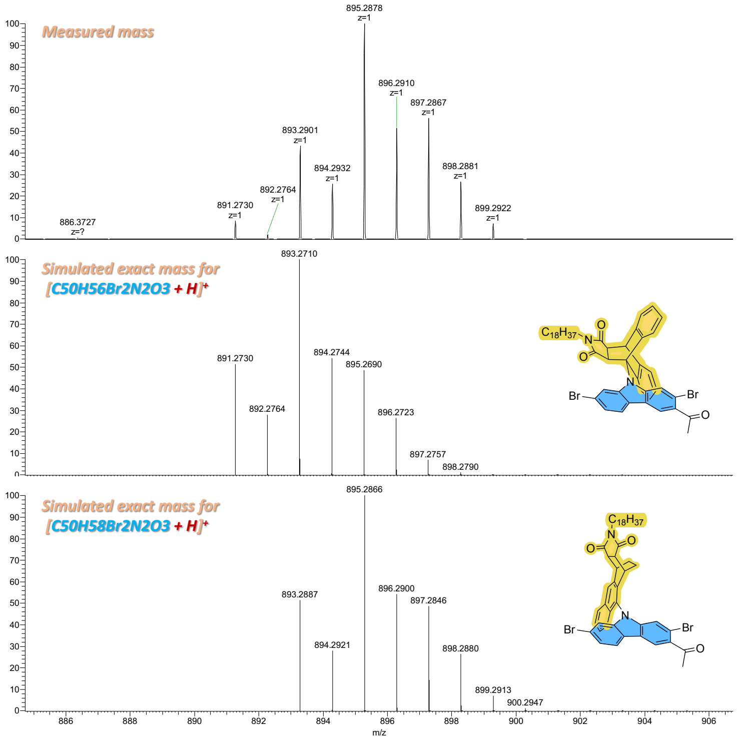

Figure S46. Measured APCI-HRMS of atropisomer mixture **11-*α*** and **12-*α*** and their simulated exact masses.

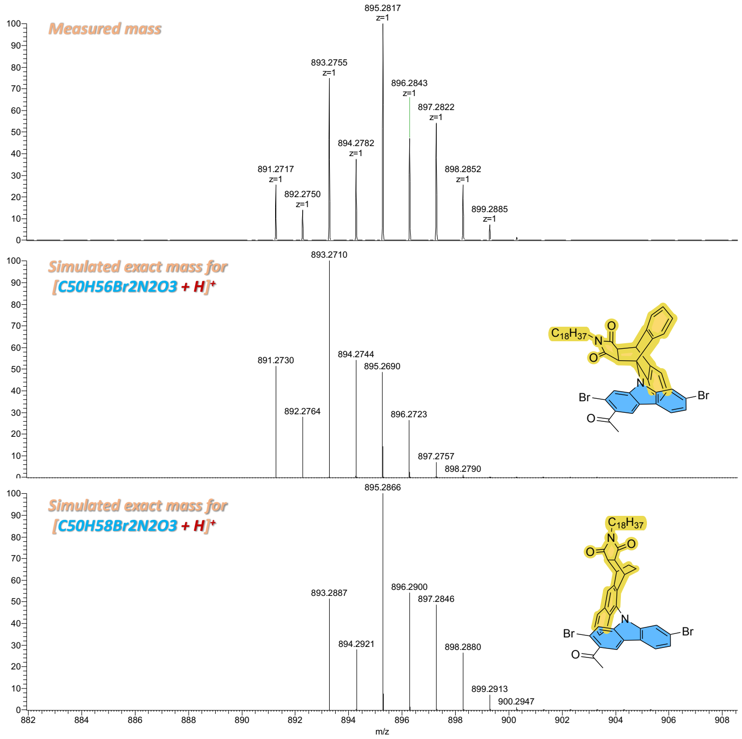

Figure S47. Measured APCI-HRMS of atropisomer mixture **11-*β*** and **12-*β*** and their simulated exact masses.

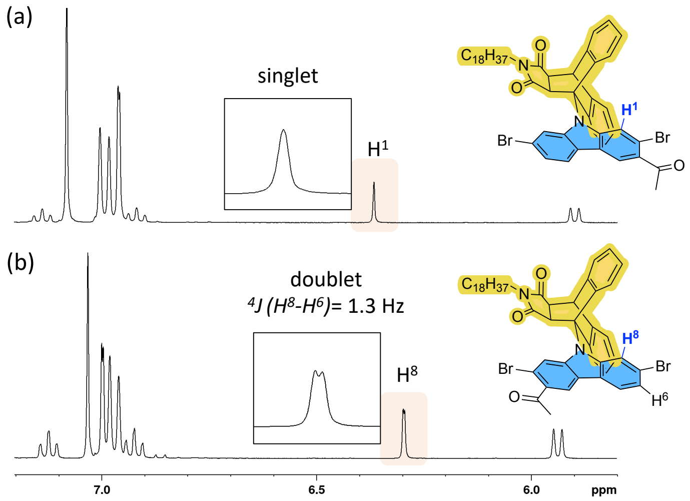

Figure S48. Partial ^{1}H NMR (500 MHz, $CDCl_3$, 25 ºC) spectra showing (a) the singlet H^1 of atropisomer **11-*α*** and (b) the doublet H^8 of atropisomer **11-*β***.

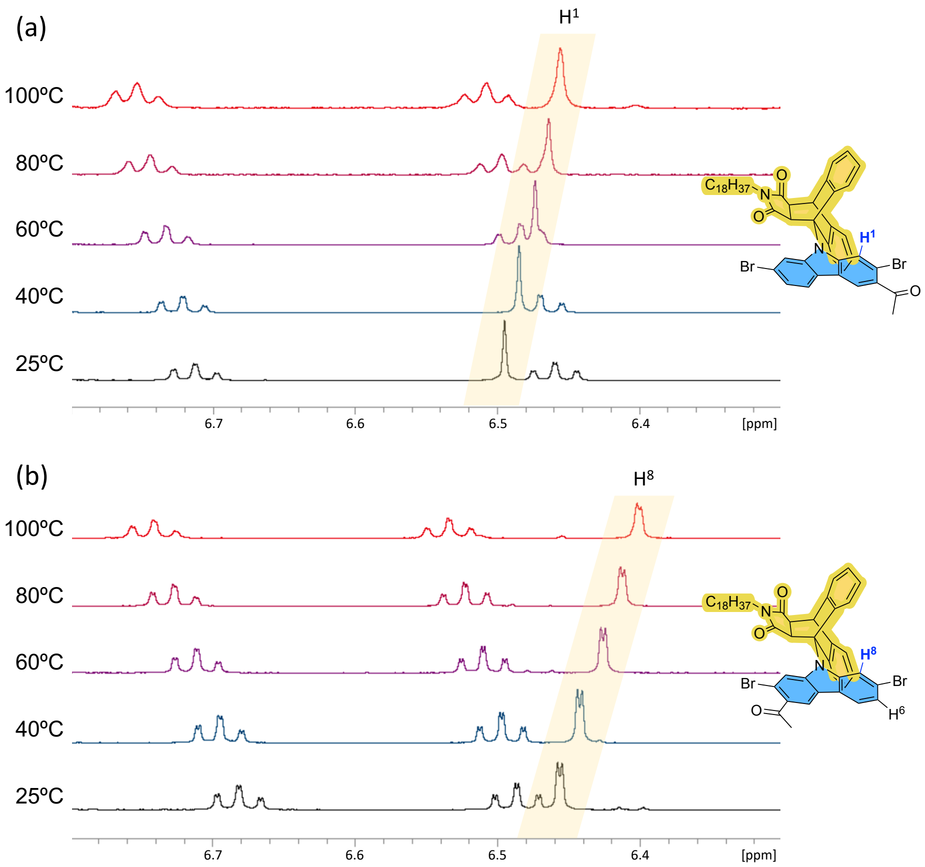

Figure S49. Partial variable-temperature ^{1}H NMR (500 MHz, toluene-d8) spectra showing (a) the singlet H^1 of atropisomer **11-*α*** and (b) the doublet H^8 of atropisomer **11-*β*** across 25–100ºC. Retention of multiplicity suggests the absence of atropisomer interconversion.

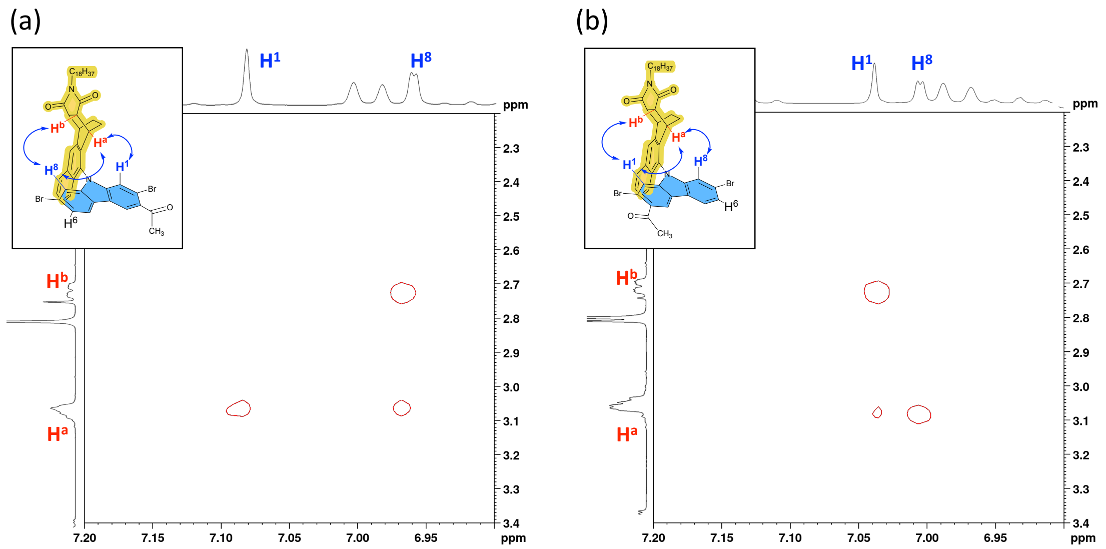

Figure S50. Partial ^{1}H-^{1}H NOESY (400 MHz, $CDCl_3$, 25 ºC) spectra showing **(a)** atropisomer **12-**α with a cross-peak from H^b to H^8 and **(b)** atropisomer **12-**β with a cross-peak from H^b to H^1.

6. Determination of Hindered Rotation

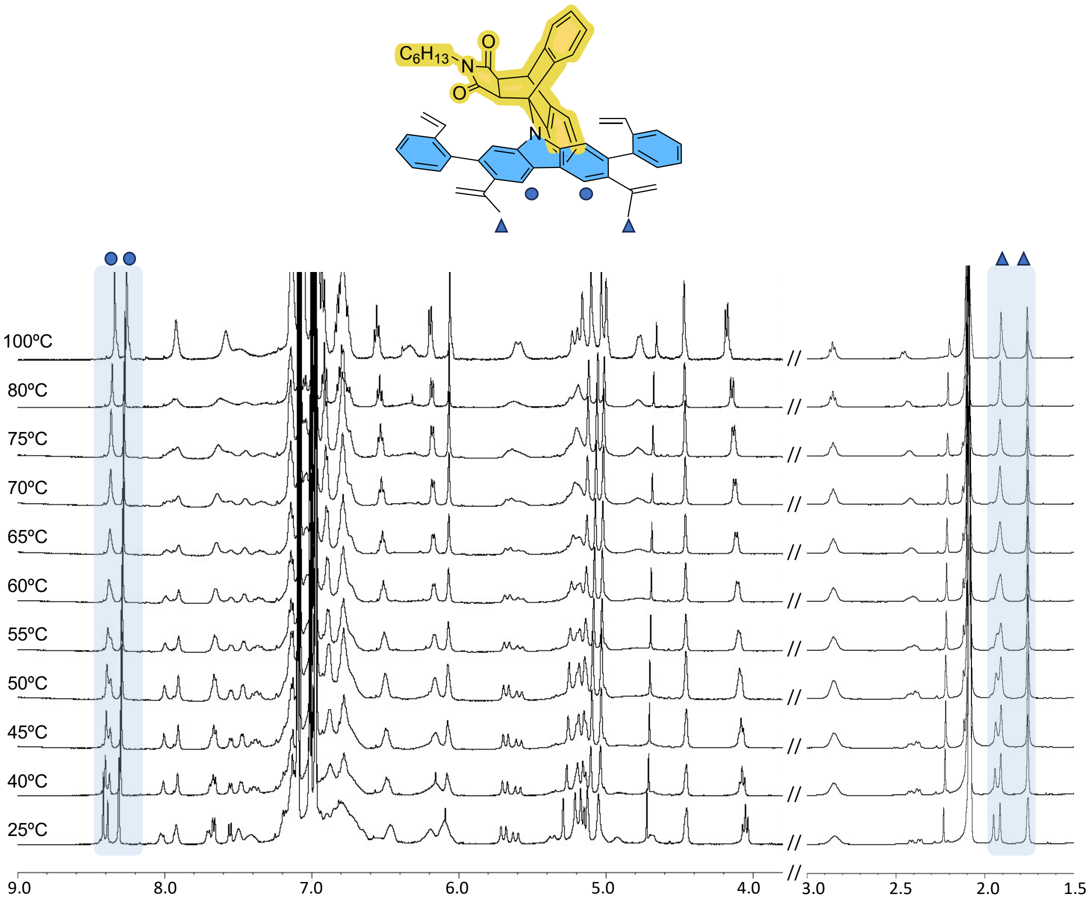

Figure S51. Partial variable-temperature ^1H NMR (500 MHz, toluene-d8) spectra of **6** showing the hindered rotation of end-capped styrene and its coalescence temperature at 60ºC.

7. Photophyisical Properties

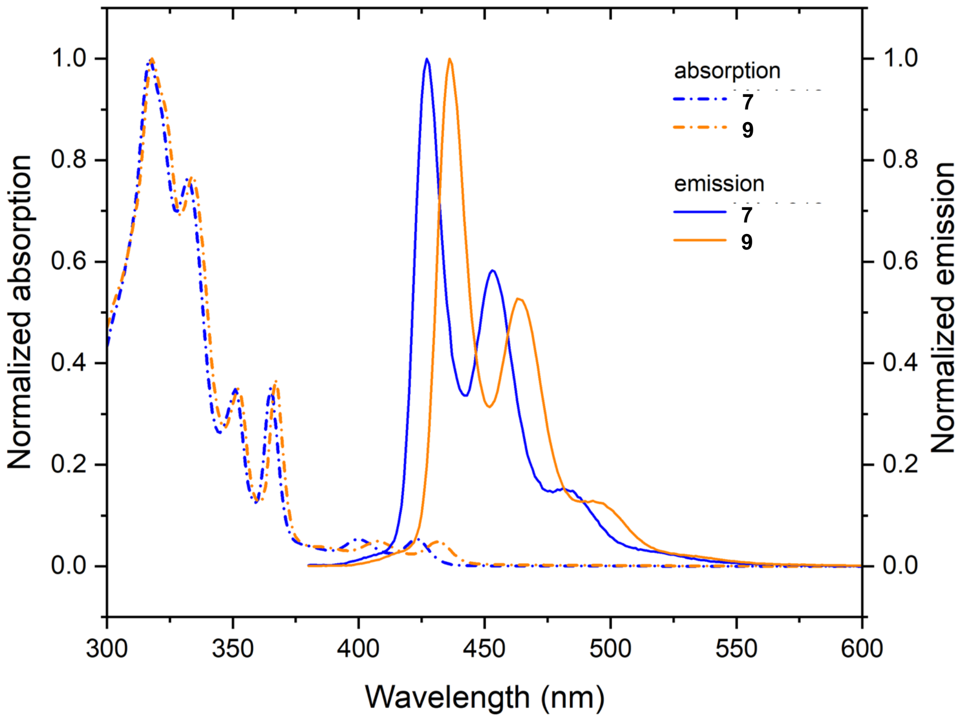

Figure S52. UV-Vis absorption (dashed lines) and photoluminescence (solid lines) spectra of **7** and **9**.

8. Determination of Retro-Diels-Alder Reaction

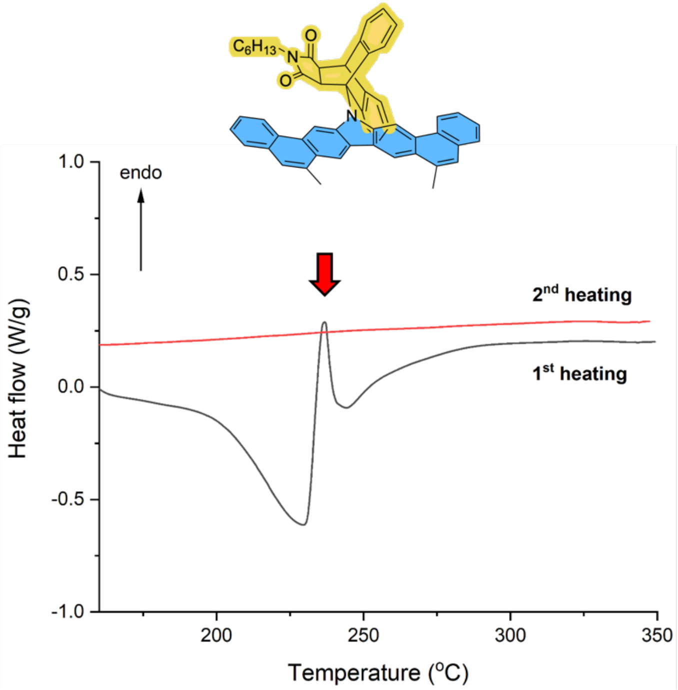

Figure S53. DSC thermogram of **7** shows the retro-Diels-Alder reaction proceeds at 240ºC, while the retro-Diels-Alder temperature of free AMA was found to be higher than 300ºC.

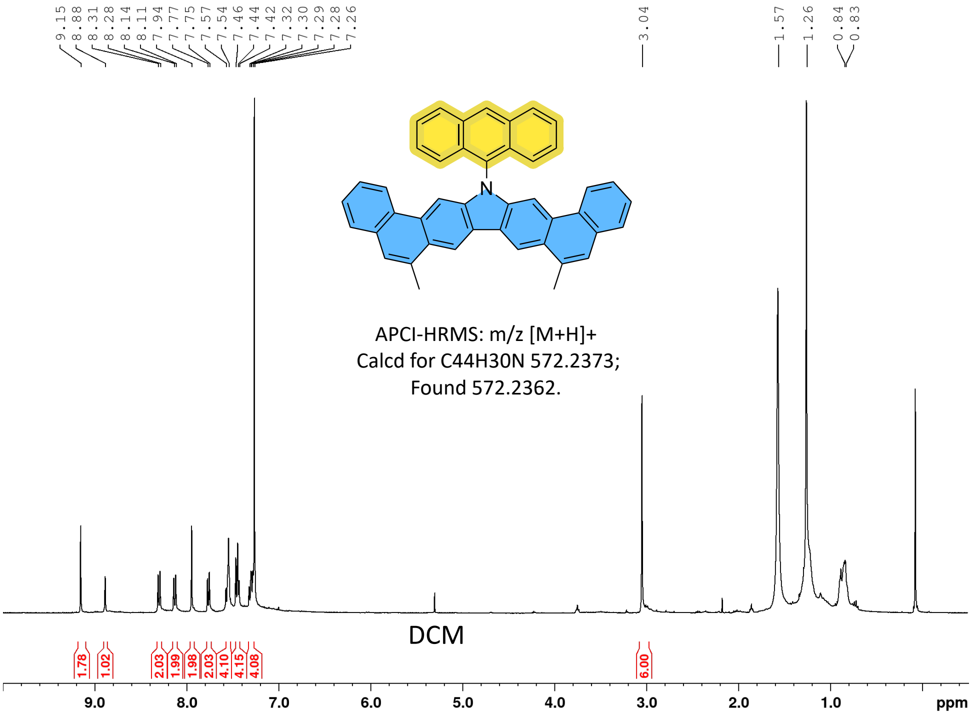

Figure S54. ^{1}H NMR (400 MHz, $CDCl_3$, 25 ºC) spectrum of thermolysis crude product of **7**, showing quantitative conversion to the retro-Diels-Alder product. The thermogram is shown in **Figure S53**.

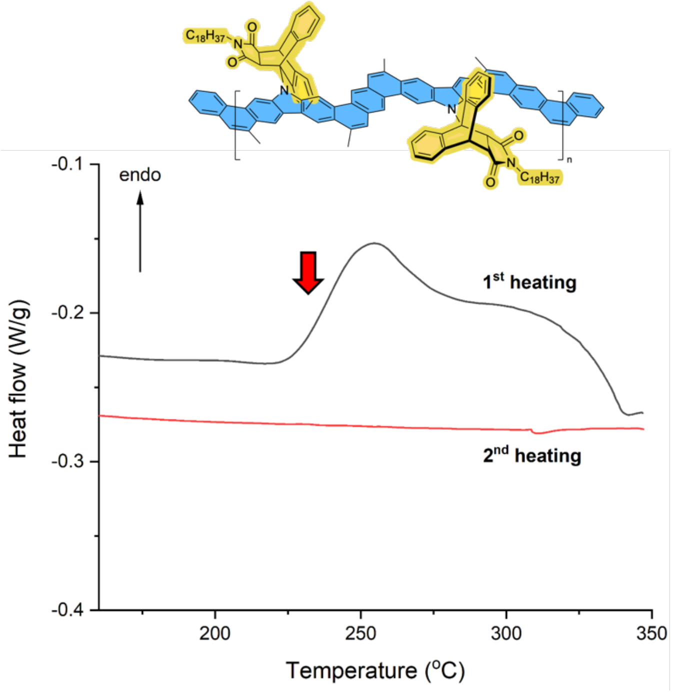

Figure S55. DSC thermogram of **LP-1** shows the retro-Diels-Alder (rDA) reaction proceeds at 240 ºC, similar to that of **7**.

9. Size Exclusion Chromatograms (SEC)

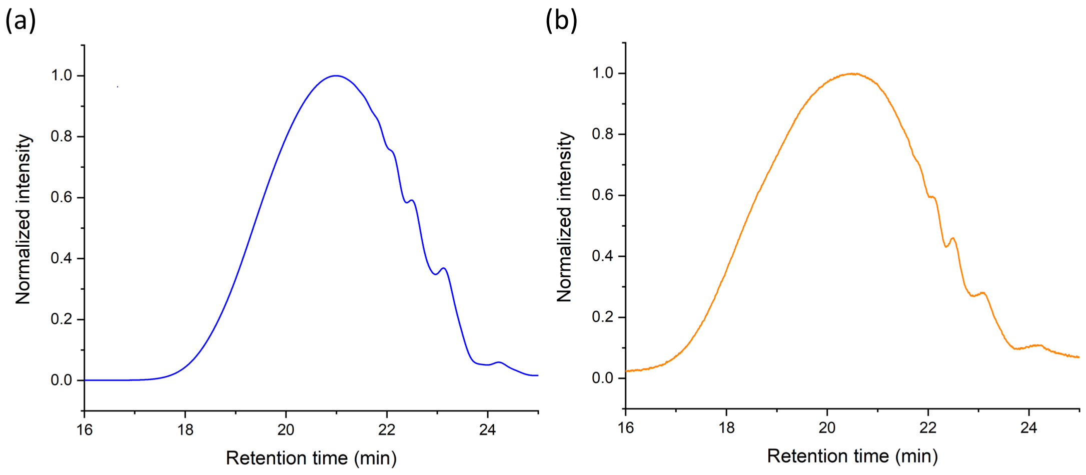

Figure S56. SEC trace of **(a) P1** (M_n= 8.2 kg/mol, $Đ$= 2.02) before fractionation and **(b) P2** (M_n= 10.7 kg/mol, $Đ$= 2.42) before fractionation.

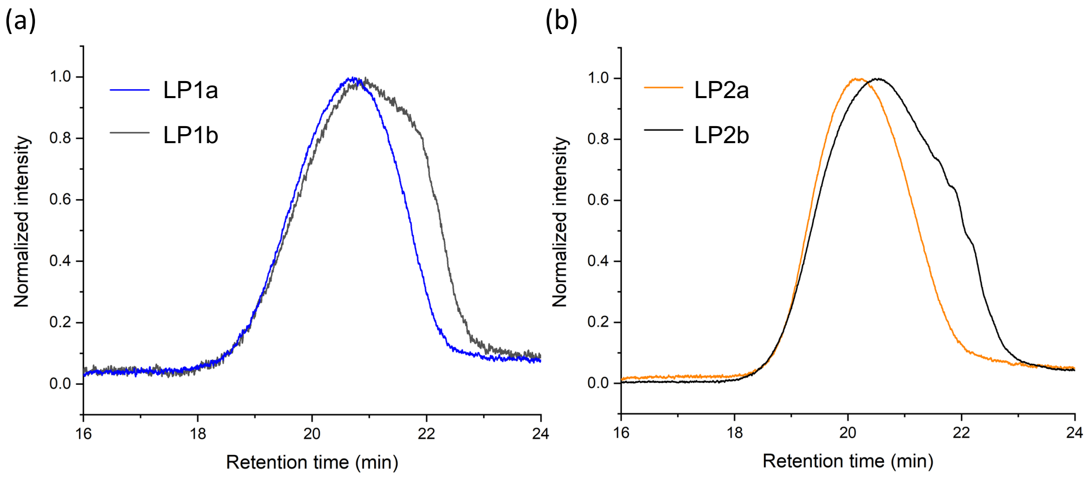

Figure S57. SEC traces of **(a) LP1a** (M_{n}= 16.7 kg/mol, $Đ$= 1.40) and **LP1b** (M_{n}= 10.0 kg/mol, $Đ$= 1.60) and **(b) LP2a** (M_{n}= 16.2 kg/mol, $Đ$= 1.30) and **LP2b** (M_{n}= 10.1kg/mol, $Đ$= 1.60). The polymer fractions were fractionated through recycling preparative SEC.

10. Dynamic Light Scattering (DLS)

LP1 and **LP2** were dissolved in chlorobenzene at 10 mg/ml and were heated at 80 ºC for 1 hr. After cooling to room temperature, the solutions were further filtered through syringe filters with a pore size of 200 nm, and the filtrates were used for DLS measurements. DLS was performed on a Brookhaven BI-200SM research goniometer with BI-APD avalanche photodiode detector and 35mW 633nm laser source (vertically polarized) with right-angle geometry. For depolarized DLS measurements, a rotatable Glan-Thompson polarizer was introduced in front of the detector for depolarization. Specifically, VV and VH denote the polarizer's orientation in the vertical and horizontal directions, respectively. An autocorrelation function, C(t), is calculated based on the fluctuation signal:

$$C(t) = A\, e^{\{-2\Gamma t\}} + B$$

where A is an optical constant determined by the instrument setup, Γ is the relaxation rate of the fluctuations, t is time, and B is a constant background offset. The relaxation rate Γ is related to the diffusion coefficient D and the scattering vector q by the relation $\Gamma = D^*q^2$. The scattering vector q is defined as:

$$q = \frac{4\pi n_0}{\lambda_0} \sin\frac{\theta}{2}$$

where n_0 is the refractive index of the medium, λ_0 is the laser wavelength in vacuum, and θ is the scattering angle. The size distribution of the particles was analyzed using Brookhaven software through cumulant analysis.

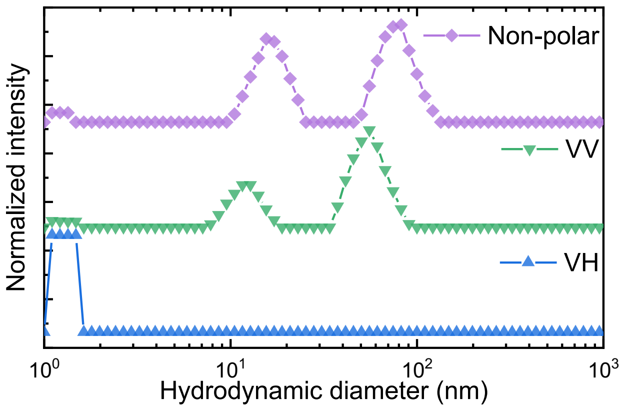

Figure S58. Depolarized DLS of **LP2** in chlorobenzene. The absence of signal in VH mode suggests that the bimodal distribution is not a result of anisotropy. The two signals at *c.a.* 20 nm and 100 nm are attributed to the non-aggregated polymer chains and polymer aggregates, respectively.

11. Small-Angle Neutron Scattering (SANS)

LP1a, **LP1b**, **LP2a**, and **LP2b** were dissolved in deuterated 1,2-dichlorobenzene (1,2-DCB-d4) at 10 mg/ml and then heated at 100 ºC for 3 hr. After cooling to room temperature, the solutions were further filtered through syringe filters with a pore size of 200 nm. The filtrates were placed in Banjo cells with a beam path of 2 mm and were used for SANS measurements. The experiments were performed at 130 ºC. The scattering intensity was reduced by subtracting the background from blank solvents and cells and placed on an absolute scale (cm^{-1}) using a standard porous silica sample. The scattering signal was analyzed using the SasView software and fitted with flexible cylinder model. In the modeling fitting, the contour length L_c, and persistence length L_p, as well as the radium of the cylinder R, were fitted based on the previous published procedures[6-8].

12. Orbital analysis of LP1 and LP2

Density functional theory (DFT) calculations were performed at the ωB97X-D3BJ/def2-TZVP[9, 10] level employing ORCA software[11] (version 6.1.0) using trimeric segments of the polymers, fully optimized for orbital analysis. Molecular orbital decomposition was carried out using Multiwfn[12], where the fused conjugated ring system was treated as one fragment and all substituents as another.

Table S4 shows the decomposition for fused rings and substituents as two fragments. For **LP1**, the conjugated backbone accounts for 97.2% and 94.3% of the HOMO and LUMO compositions, respectively, confirming that both orbitals remain delocalized across the π-conjugated core. In **LP2**, however, the HOMO remains predominantly on the conjugated ring system (99.7%), while the LUMO contribution from the backbone decreases dramatically to 13.2%, with the remaining density localized on the substituent moieties. The orbital isosurface (0.02 a.u) representations (**Figure S59** and **Figure S*60***) consistently show that the substituents in **LP2** perturb the LUMO through both electronic and steric effects: steric crowding around the C–N bond reduces conjugation of the backbone and side chain, while the electron-withdrawing nature of the substituent stabilizes its local LUMO level. Together, these effects result in an electronically decoupled side unit and a distinct backbone-to-substituent charge-transfer character. The orbitals were rendered using Chemcraft visualization program[13].

Table S4. Orbital contribution from conjugated backbone and substituents for **LP1** and **LP2**.

**Polymers**	***Conjugated backbone***		***Substituents***	
	HOMO %	LUMO %	HOMO %	LUMO %
LP1	97.2	94.3	2.8	5.7
LP2	99.7	13.2	0.3	86.8

LP1 (oligomer, trimer)

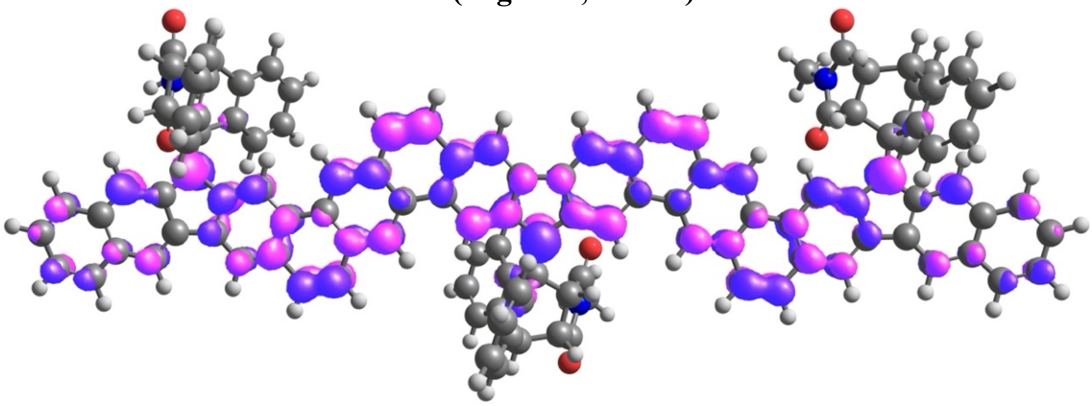

Top view of HOMO

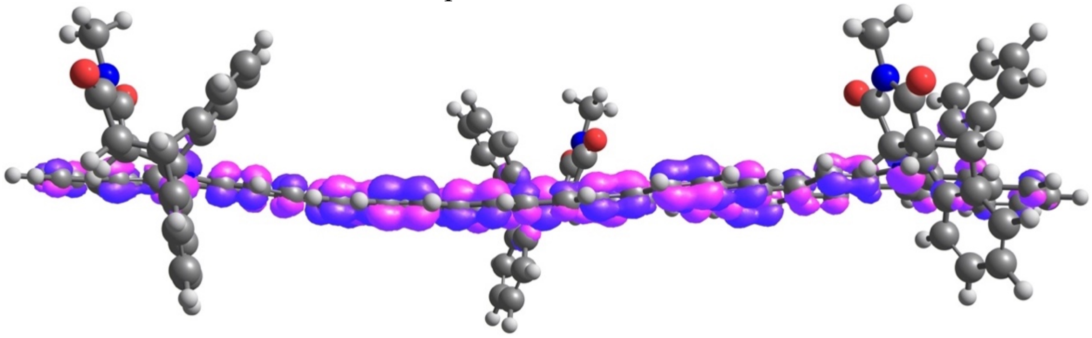

Side view of HOMO

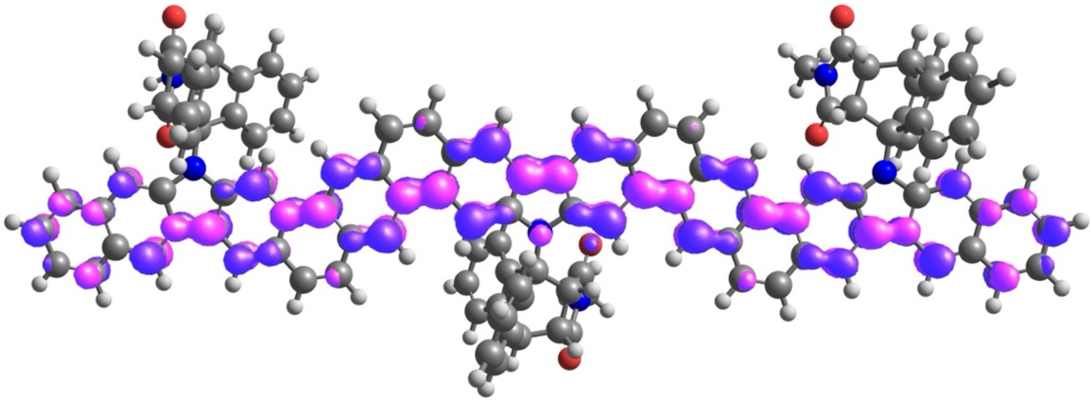

Top view of LUMO

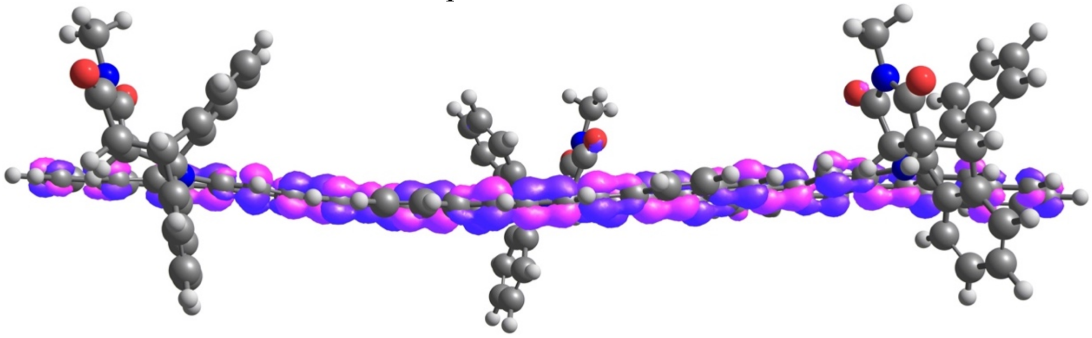

Side view of LUMO

Figure S59. HOMO and LUMO isosurface (0.02 a.u) images of **LP1.**

LP2 (oligomer, trimer)

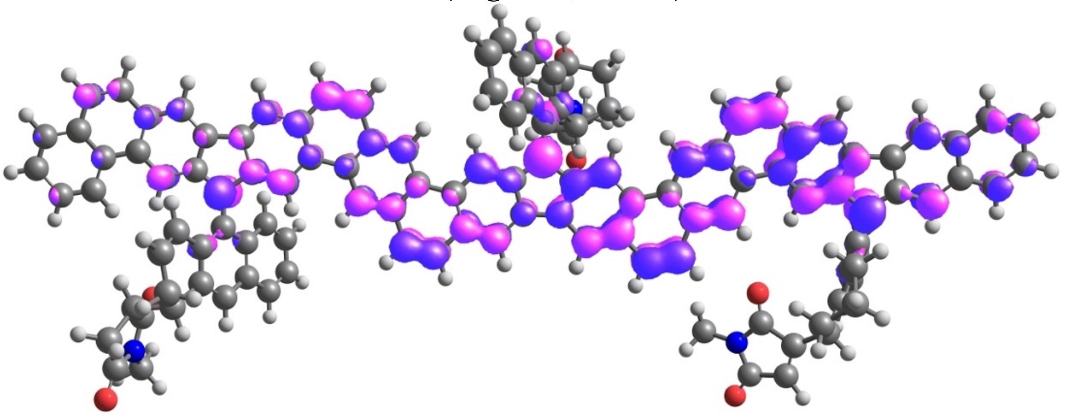

Top view of HOMO

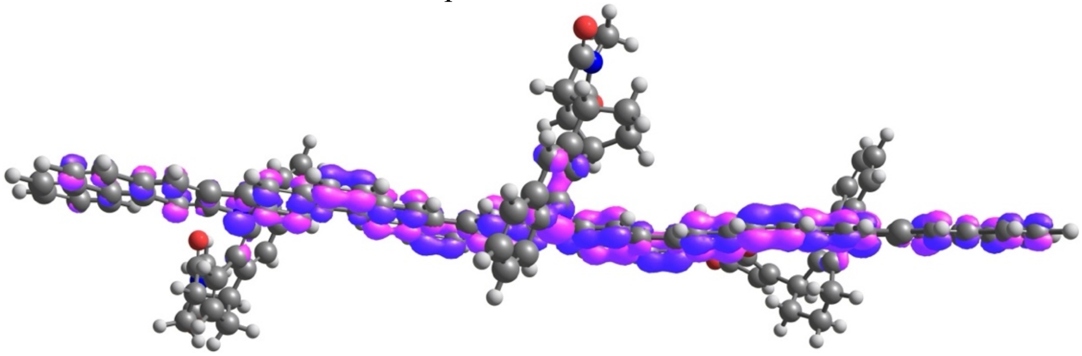

Side view of HOMO

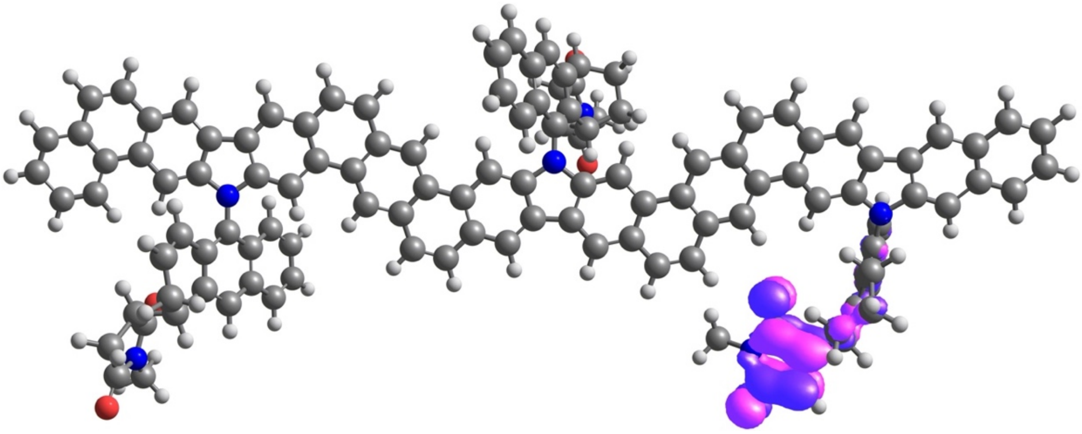

Top view of LUMO

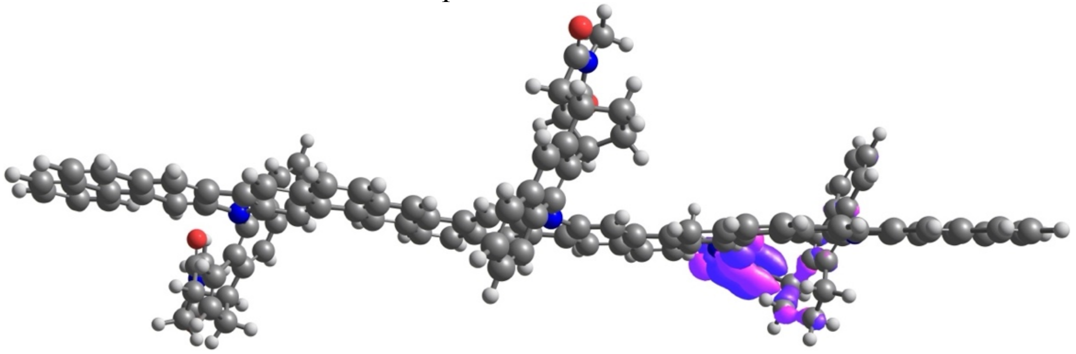

Side view of LUMO

Figure S60. HOMO and LUMO isosurface (0.02 a.u) images of **LP2**.

13. Molecular Dynamics Simulation

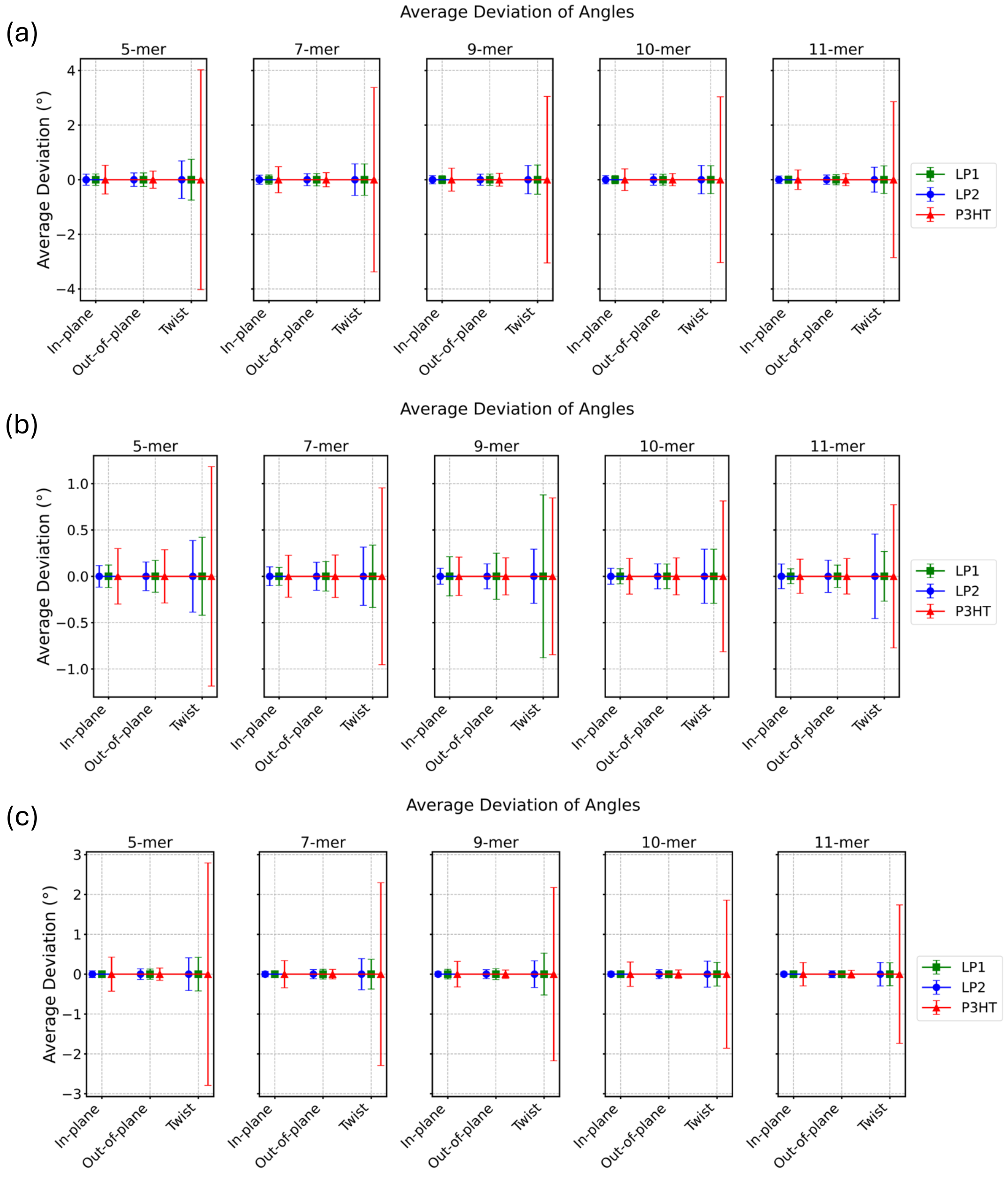

Figure S61. Deviations in angles from equilibrium values across different polymers, lengths for force fields: (a) OpenFF, (b) GAFF, (c) AIMNET2.

To assess the rigidity of **LP1** and **LP2** polymers relative to **P3HT**, molecular dynamics (MD) simulations were conducted across various chain lengths. Specifically, oligomers of 5-, 7-, 9-, 10-, and 11-mers for **LP1** and **LP2**, and corresponding **P3HT** oligomers of 14, 21, 26, 28, and 32 thiophene units were selected to match the contour lengths of

the ladder polymers. For uniformity, these **P3HT** oligomers are denoted as **P3HT-n-mers**, where n corresponds to the ladder polymer lengths.

Figure S61 shows the deviation in in-plane bending, out-of-plane, and twist angles for multiple lengths and type of polymers calculated at different forcefields. Using the OpenFF force field, in-plane bending angle (θ_{ipb}) deviations were modest, averaging 0.01–0.45°. Out-of-plane bending (θ_{oop}) deviations were even smaller, approximately 0.04–0.10°, indicating high rigidity in these degrees of freedom. In contrast, twist angle (θ_{twist}) deviations for **LP1** and **LP2** averaged around 0.75–0.94°, suggesting slightly higher torsional flexibility. **P3HT** exhibited significantly greater twist angle variability, averaging around 4.0°, highlighting pronounced torsional flexibility.

The GAFF force field simulations showed overall smaller angular deviations, indicative of increased rigidity compared to OpenFFT. For θ_{ipb}, **LP1** and **LP2** exhibited deviations of approximately 0.15º–0.20º, while **P3HT** showed slightly higher deviations at 0.65º–0.70º. θ_{oop} deviations for **LP1** and **LP2** ranged from 0.30º–0.34º, with **P3HT** comparable at 0.30º–0.45º. Twist angle deviations under GAFF were markedly lower for ladder polymers (0.36º–0.45º) relative to **P3HT** (0.75º–1.50º), reinforcing the conclusion that GAFF imposes greater torsional rigidity.

The AIMNET2 simulations provided complementary insights consistent with classical force field trends. Deviations in θ_{ipb} for **LP1** and **LP2** ranged from approximately 0.25º–0.50º. θ_{oop} deviations remained minimal, averaging 0.10º–0.15º across both ladder polymers and **P3HT**. However, θ_{twist} demonstrated substantially greater deviations for **P3HT**, ranging from 1.64º–2.74º, compared to ladder polymers, which exhibited significantly lower deviations of 0.43º–0.52º.

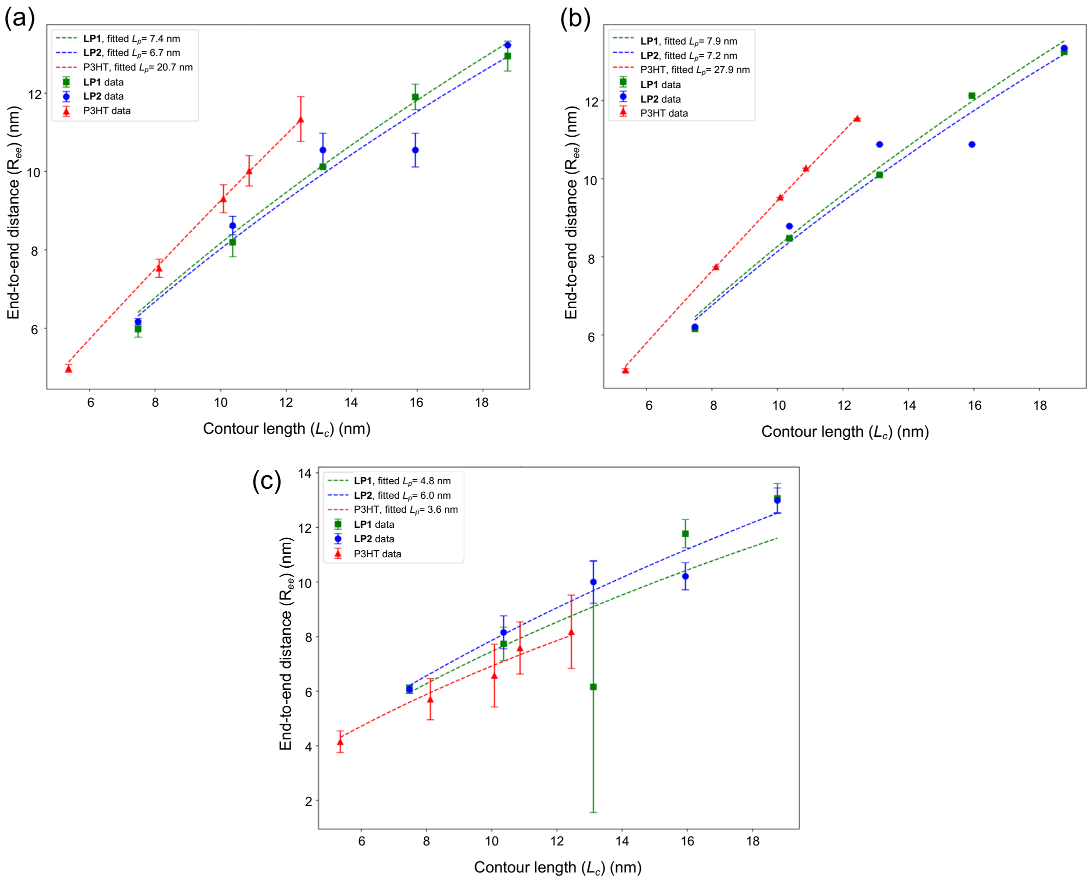

Figure S62. End-to-end distance (R_{ee}, nm) over contour length (L_c, nm) with WLC fit using (a) OpenFF, (b) GAFF, and (c) AIMNET2.

Figure S62 shows the fit of R_{ee} and L_c using the worm-like chain (WLC) model.[14, 15] For all methods, linear correlation between R_{ee} and L_c is observed. It is noteworthy that the polymer chain lengths in this study are shorter compared to those simulated elsewhere. Specifically, for **P3HT**, we observe a longer persistence length of 20.6–27.9 nm from OpenFF and GAFF simulations, indicating that the shorter chain lengths used here may not adequately estimate **P3HT**'s persistence length. Janke et al. observed a linear correlation between R_{ee} and chain length at similar temperatures for shorter polymer lengths.[16] Nevertheless, the plot shows that R_{ee} is less than the fully extended polymer length for all systems, indicating flexibility. However, OpenFF and GAFF results suggest that the R_{ee}/L_c ratio is smaller for ladder polymers, indicating that **LP1**

and **LP2** may be trapped in conformations with shorter R_{ee}, reducing their ability to move freely and resulting in lower R_{ee}. This is partly due to the force field's insufficiency in allowing polymer flexibility, as evident from **Figure S62c**. The fit obtained from AIMNET2 simulation data shows that this potential allows higher polymer flexibility, providing an approximate persistence length (L_p) of 3.6 nm for **P3HT**, closer to the experimental value of approximately 3.0 nm. AIMNET2 simulations also estimate persistence lengths of 4.8 nm and 6.0 nm for **LP1** and **LP2**, respectively, which are still larger than **P3HT**.

These findings highlight the effect of force fields on polymer persistence length. AIMNET2-based simulations allow greater polymer flexibility, yielding better persistence length estimates. Although the shorter 2-ns trajectory for AIMNET2 simulations may not provide high accuracy for longer timescale results, the qualitative comparisons effectively illustrate flexibility differences among the three polymers.

Computational Details

To investigate and compare the conformational flexibility of CLP polymers against poly(3-hexylthiophene) (P3HT), computational modeling and molecular dynamics simulations were carried out. Two CLP polymers, namely **LP1** and **LP2**, were modeled with oligomer lengths comprising 5, 7, 9, 10, and 11 repeating units and named as **LP1**-{n}mer and **LP2**-{n}mer where n = 5, 7, 9, 10 and 11. For comparative analysis, **P3HT** polymers were modeled with 14, 21, 26, 28, and 32 thiophene repeating units, selected to match the contour lengths of CLP polymers closely. For direct comparison between polymers the **P3HT** systems are names as P3HT-{n}mer, where n = 5, 7, 9, 10 and 11 corresponding to 14, 21, 26, 28 and 32 units respectively.

The polymer structures of varying lengths were originally drawn in ChemDraw and their xml data was converted to XYZ coordinates using Open Babel[17]. The structures were relaxed and further optimized by GFN2-xTB[18] method. Further conformer search for each length was carried out using CREST[19] method at GFN-FF level of theory. The lowest energy conformer serves as starting structure for molecular simulation.

All molecular dynamics (MD) simulations employed two force fields: the Open Force Field (OpenFF) unconstrained force field (version 2.2.0, openff_unconstrained-2.2.0.offxml[20]) and the General Amber Force Field[21] (GAFF version 2.11). Force-field typing was performed using the OpenFF Toolkit[22]. Atomic partial charges for both force fields were derived from restrained electrostatic potential (RESP)[23] fitting. RESP charges were computed from density functional theory (DFT)-optimized trimer structures terminated with hydrogens, using the ωB97X-D3BJ/def2-TZVP level of theory in ORCA software.[9, 10, 24] Boundary atomic charges were averaged to ensure charge continuity across repeating units, and excess charges were uniformly adjusted to maintain electroneutrality of each polymer segment.

Classical MD simulations were conducted using OpenMM[25]. Each polymer was simulated at a constant temperature of 403 K in the gas phase, employing an implicit solvent model with a dielectric constant (ε) of 5.7 to mimic chlorobenzene. Simulations included an initial equilibration period of 10 ns, followed by a production run of 100 ns with a 2 fs integration timestep with hydrogens constrained using SHAKE algorithm. For statistical robustness, three independent simulations were conducted for each polymer configuration, initiated with distinct random velocity distributions.

To validate the forcefields employed for ladder polymers, AIMNET2[26], an ML-based potential, was used to simulate the polymers under study. AIMNET2 potentials has

been shown to provide interaction energies with accuracies similar to that of DFT and semi-empirical methods. Initial coordinates and velocities for AIMNET2 simulations were extracted from the final snapshots of OpenMM trajectories. Each system was allowed a brief 50 ps equilibration period under AIMNET2 dynamics to stabilize structures following force-field transition. AIMNET2-based production simulations proceeded for 2-ns under Langevin dynamics at 403 K with a 1-fs timestep, without implicit solvent, utilizing the ASE interface[27]. Forces and energies were computed exclusively via the AIMNET2 model.

Analysis Methods

Structural analysis focused on polymer conformational parameters: the radius of gyration (R_g), end-to-end distance (R_{ee}), and inter-ring angles—in-plane bending (θ_{ipb}), out-of-plane bending (θ_{oop}), and twist angles (θ_{twist}).

For angular analysis, each set of three consecutive aromatic rings indexed as i, (i+1) and (i+2) was considered. The center of mass (COM) of each ring was computed to define vectors connecting consecutive ring COMs:

- $\boldsymbol{v}_{i,i+1}$: vector from COM of ring i to COM of ring i+1
- $\boldsymbol{v}_{i+1,i+2}$: vector from COM of ring i+1 to COM of ring i+2

Figure S63 presents the diagrammatic illustration of different angles analyzed in this study along with vectors defined above.

(a)

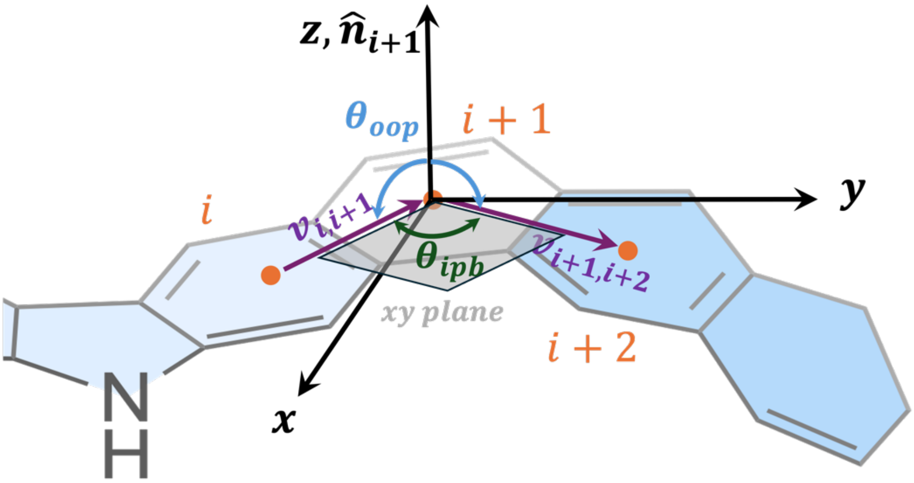

(b)

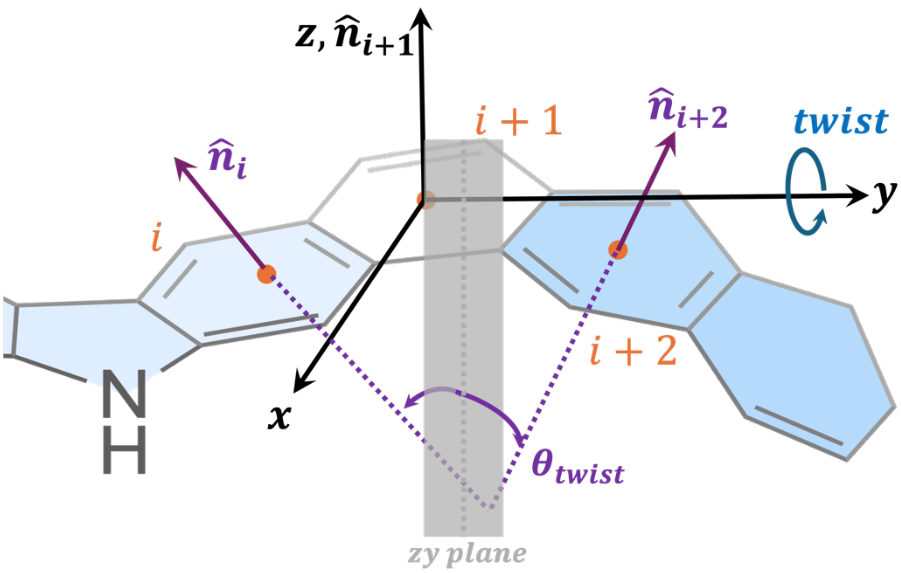

Figure S63. Diagrammatic illustration of angles analyzed in this work. (a) shows the consecutive rings i, i+1 and i+2 with COM represented as orange dots. θ_{ipb} and θ_{oop} formed by the vectors connecting COM are also illustrated. (b) presents the twist angle formed by the normal to rings i and i+2 along zy plane. The equations defining these angles are shown below.

The angular definitions are as follows:

- **In-plane bending angle (θ_{ipb})**:

$$\theta_{ipb} = cos^{-1}(\frac{v^{\parallel}_{i,i+1} \cdot v^{\parallel}_{i+1,i+2}}{|v^{\parallel}_{i,i+1} \cdot v^{\parallel}_{i+1,i+2}|}) \quad (1)$$

where vectors $\boldsymbol{v}^{\parallel}$ are projections onto the plane defined by the (i+1)th ring.

- **Out-of-plane bending angle (θ_{oop})**:

$$\theta_{oop} = sin^{-1}(\frac{(\boldsymbol{v}_{i,i+1} \times \boldsymbol{v}_{i+1,i+2}).\hat{\boldsymbol{n}}_{i+1}}{|\boldsymbol{v}_{i,i+1}||\boldsymbol{v}_{i+1,i+2}|}) \quad (2)$$

Where $\hat{\boldsymbol{n}}_{i+1}$ is the normal to the plane of ring (i+1).

- **Twist angle (θ_{twist})**:

$$\theta_{twist} = cos^{-1}(\hat{\boldsymbol{n}}_i . \hat{\boldsymbol{n}}_{i+2}) \quad (3)$$

where $\hat{\boldsymbol{n}}_i$ and $\hat{\boldsymbol{n}}_{i+2}$ are normal unit vectors to the planes of rings i and i+2, respectively.

Equilibrium angle values were obtained from the optimized DFT geometry. Angle deviations (Δθ) were computed as the difference between instantaneous MD-derived angles and the equilibrium values. Deviations were averaged over all trajectories and time steps, and error estimates were calculated using standard error propagation techniques.

The radius of gyration (R_g) and end-to-end distance (R_{ee}) were computed using MDAnalysis[28, 29]. The length (L) of each polymer was calculated from the initial, fully extended configurations using Chemcraft visualizer[30].

Results were visualized as time-evolution plots of angular deviations (Δθ), radius of gyration (R_g), and end-to-end distance (R_{ee}). Additionally, R_{ee} versus polymer length (L) data was fitted to the worm-like chain (WLC) equation[15] to understand the rigidity of polymer:

$$\langle R_{ee}^2 \rangle = 2L_pL\left[1 - \frac{L_p}{L}\left(1 - e^{-\frac{L}{L_p}}\right)\right] \quad (4)$$

Here L_p is the persistent length of the polymer. This fitting provided qualitative insight into polymer rigidity.

Trajectory snapshots of polymers of different lengths

Figure S60-S62(a-e) represents the trajectory snapshots of **LP1**, **LP2** and **P3HT** at different lengths. The snapshots were taken from the final configuration of production run from OpenFF simulation.

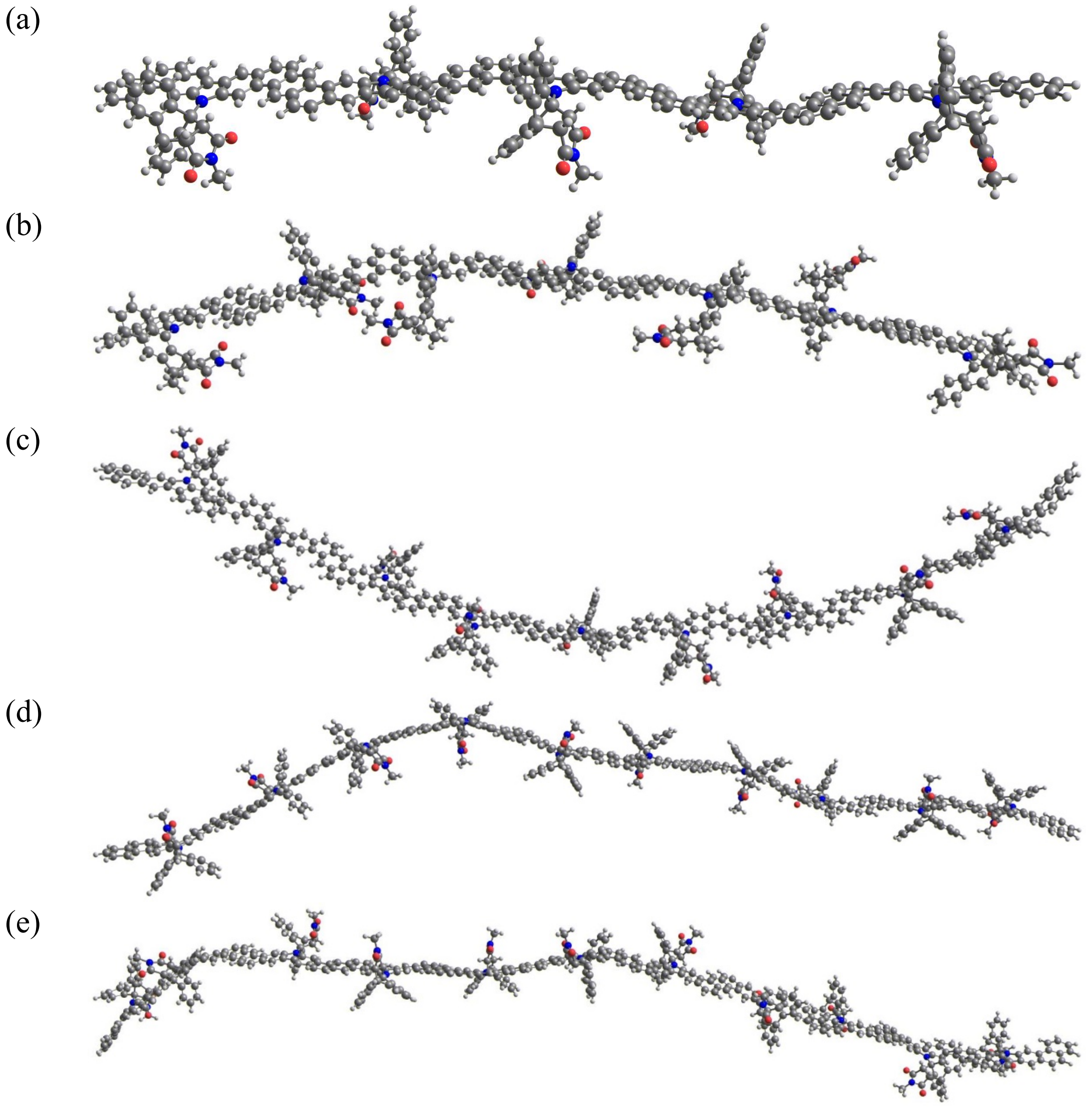

Figure S64. **(a-e)**. Snapshots of trajectory for **LP1** with a-e represents 5, 7, 9, 10, 11 mers

(a)
(b)
(c)
(d)
(e)

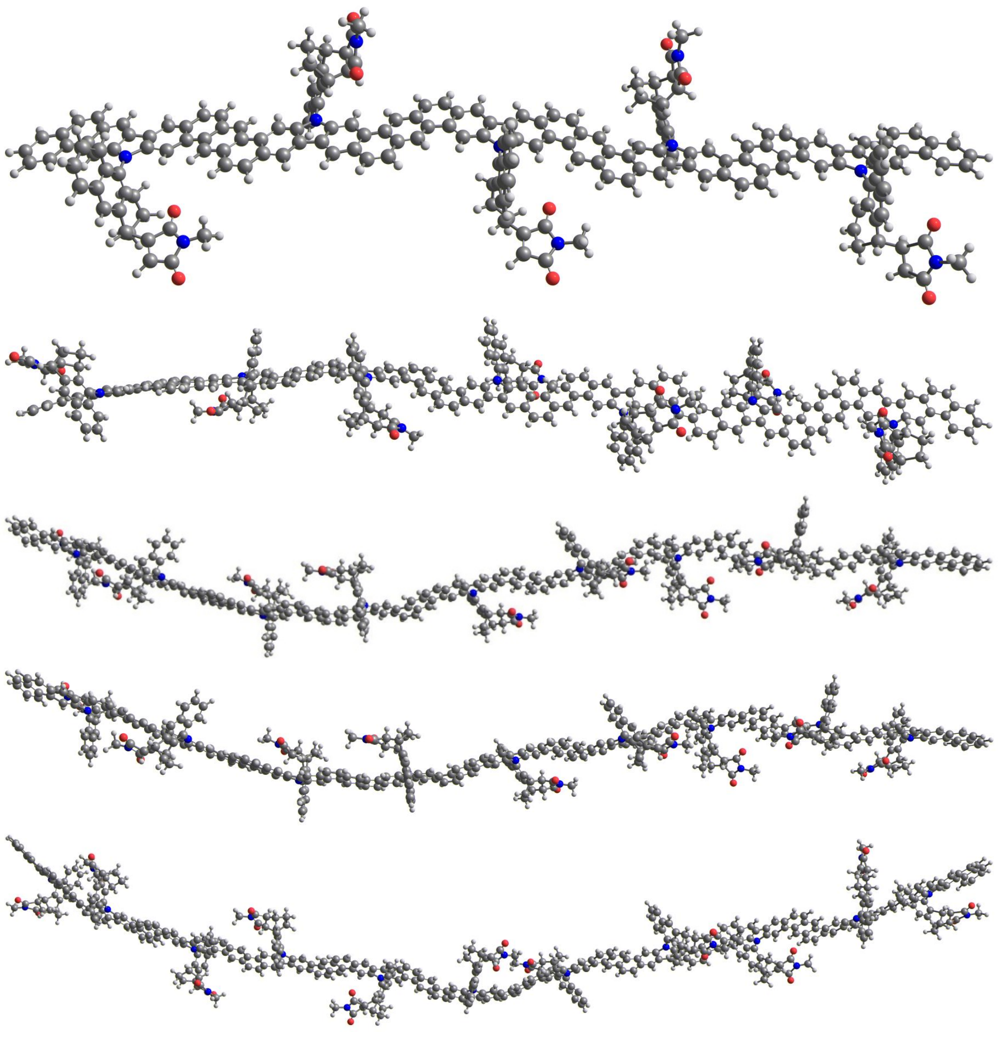

Figure S65. **(a-e)**. Snapshots of trajectory for **LP2** with a-e represents 14, 21, 26, 28 and 32 units

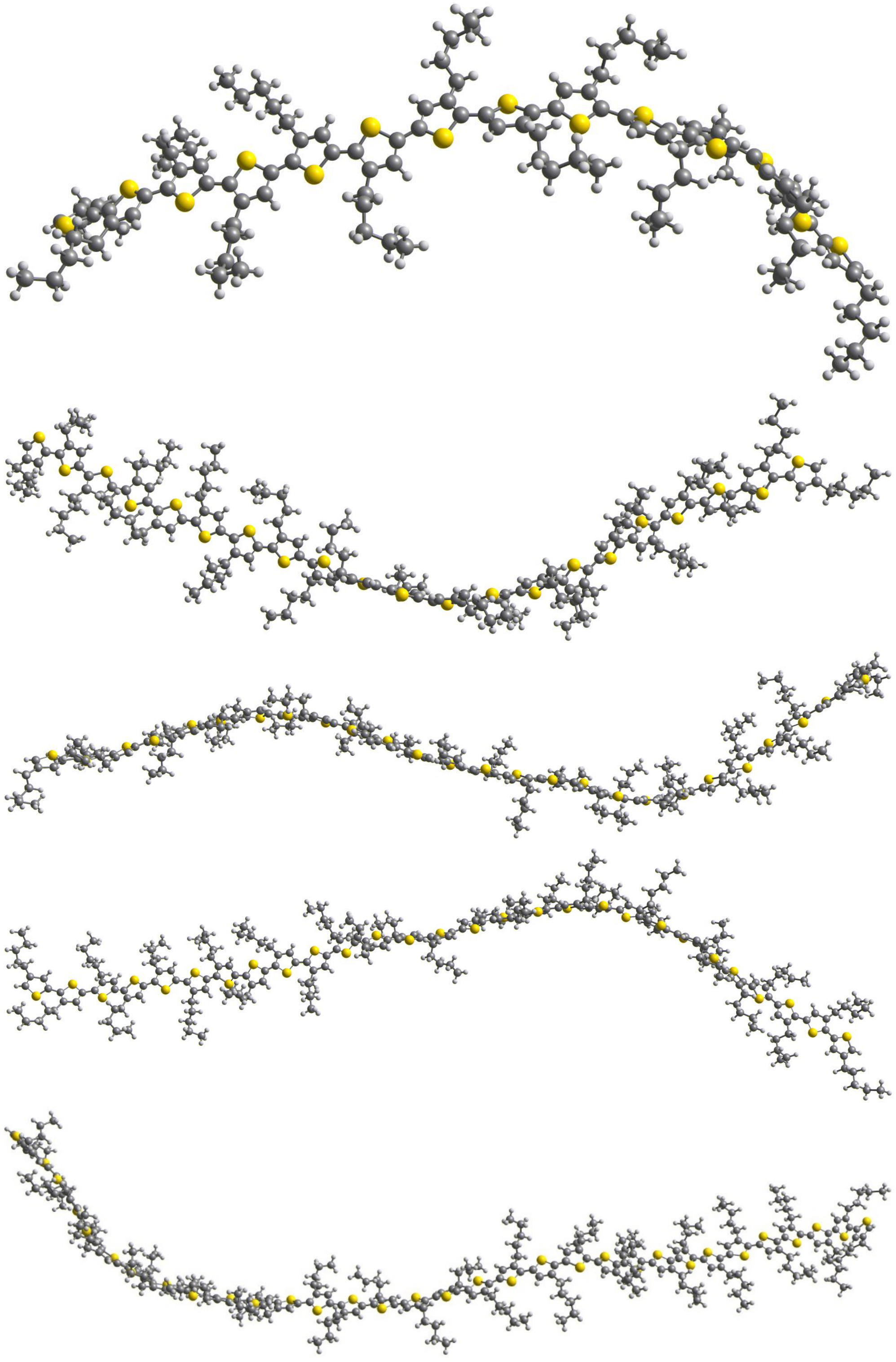

(a)

(b)

(c)

(d)

(e)

Figure S66. **(a-e)**. Snapshots of trajectory for P3HT with a-e represents 14, 21, 26, 28 and 32 units

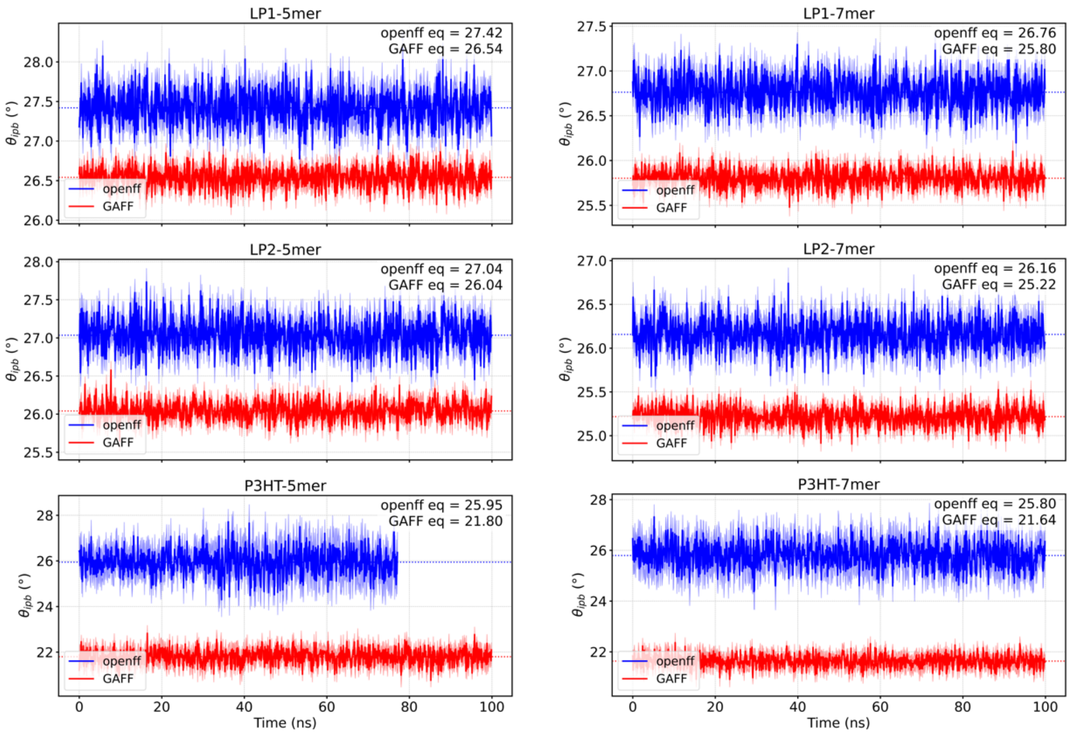
LP1-5mer
openff eq = 27.42
GAFF eq = 26.54
LP1-7mer
openff eq = 26.76
GAFF eq = 25.80
LP2-5mer
openff eq = 27.04
GAFF eq = 26.04
LP2-7mer
openff eq = 26.16
GAFF eq = 25.22
P3HT-5mer
openff eq = 25.95
GAFF eq = 21.80
P3HT-7mer
openff eq = 25.80
GAFF eq = 21.64
openff
GAFF
θipb (°)
Time (ns)

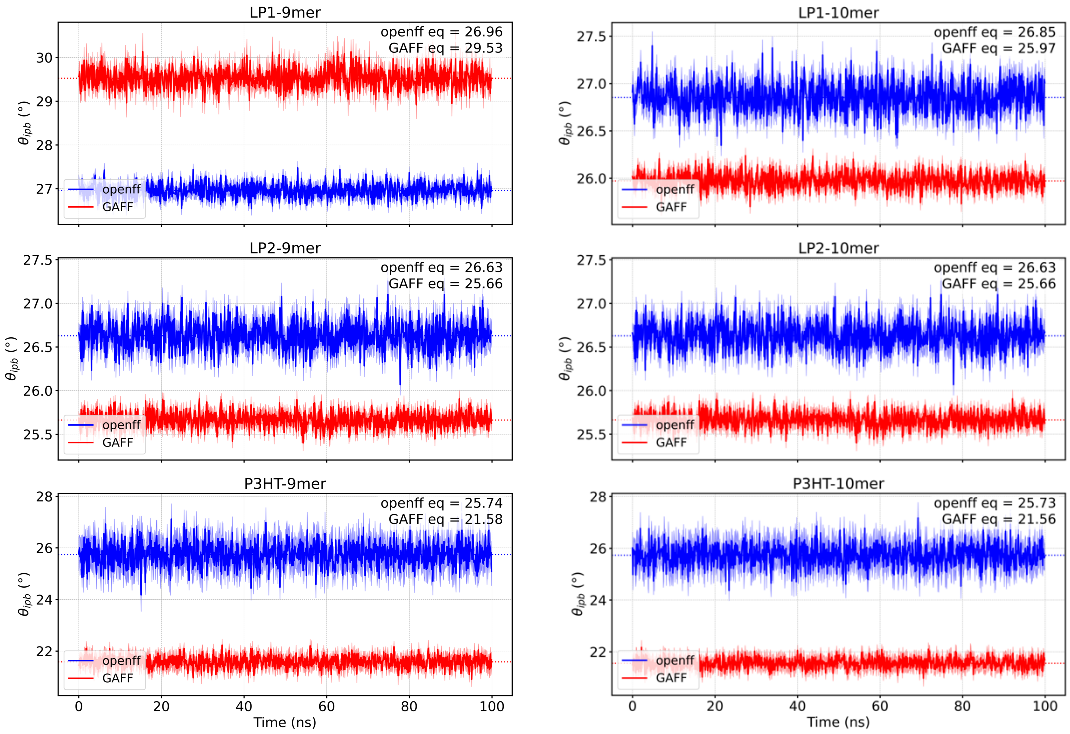
LP1-9mer
openff eq = 26.96
GAFF eq = 29.53
LP1-10mer
openff eq = 26.85
GAFF eq = 25.97
LP2-9mer
openff eq = 26.63
GAFF eq = 25.66
LP2-10mer
openff eq = 26.63
GAFF eq = 25.66
P3HT-9mer
openff eq = 25.74
GAFF eq = 21.58
P3HT-10mer
openff eq = 25.73
GAFF eq = 21.56
openff
GAFF
θipb (°)
Time (ns)

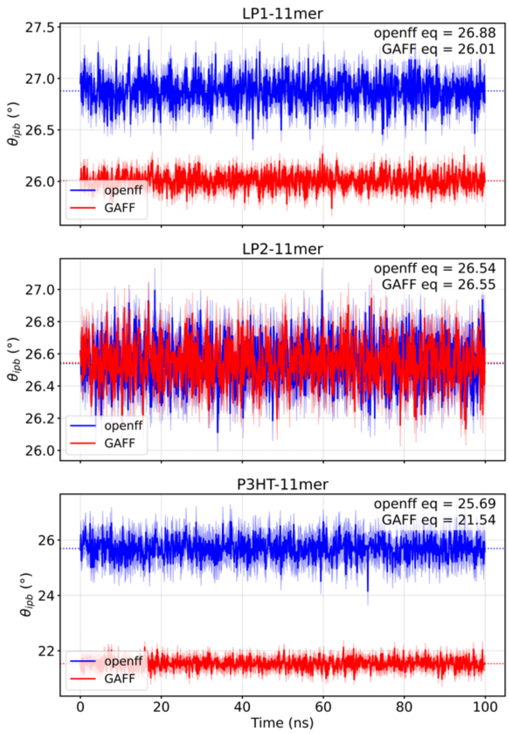
LP1-11mer
openff eq = 26.88
GAFF eq = 26.01
LP2-11mer
openff eq = 26.54
GAFF eq = 26.55
P3HT-11mer
openff eq = 25.69
GAFF eq = 21.54
openff
GAFF
θipb (°)
Time (ns)

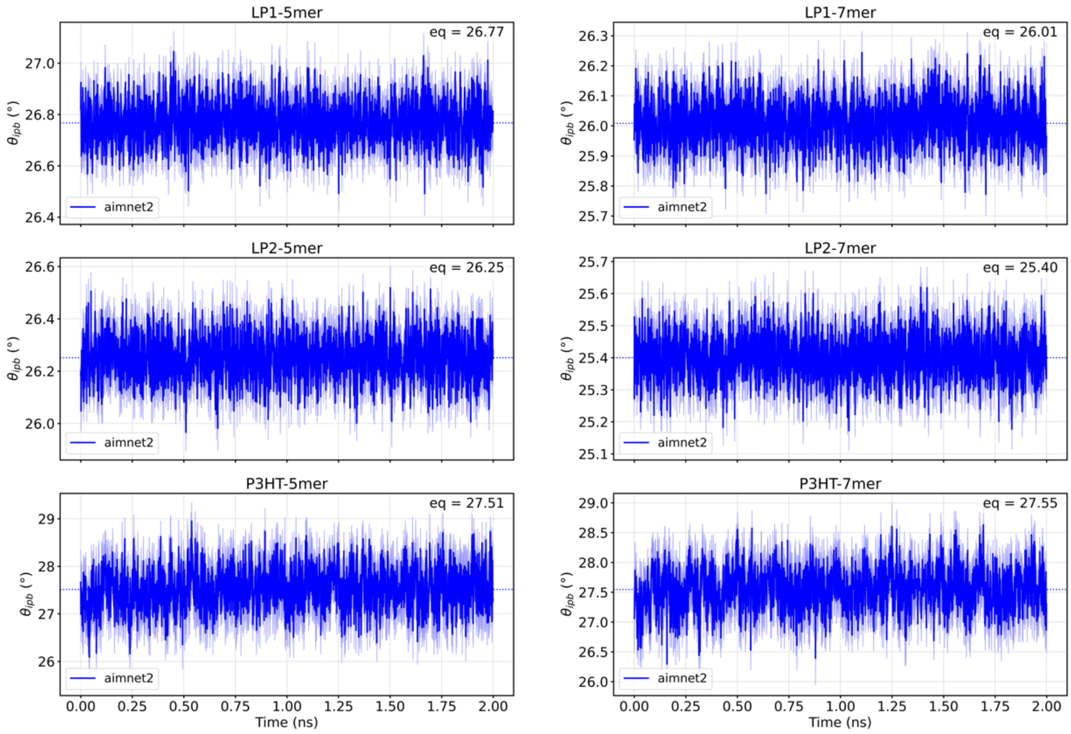
LP1-5mer
eq = 26.77
LP1-7mer
eq = 26.01
LP2-5mer
eq = 26.25
LP2-7mer
eq = 25.40
P3HT-5mer
eq = 27.51
P3HT-7mer
eq = 27.55
aimnet2
θipb (°)
Time (ns)

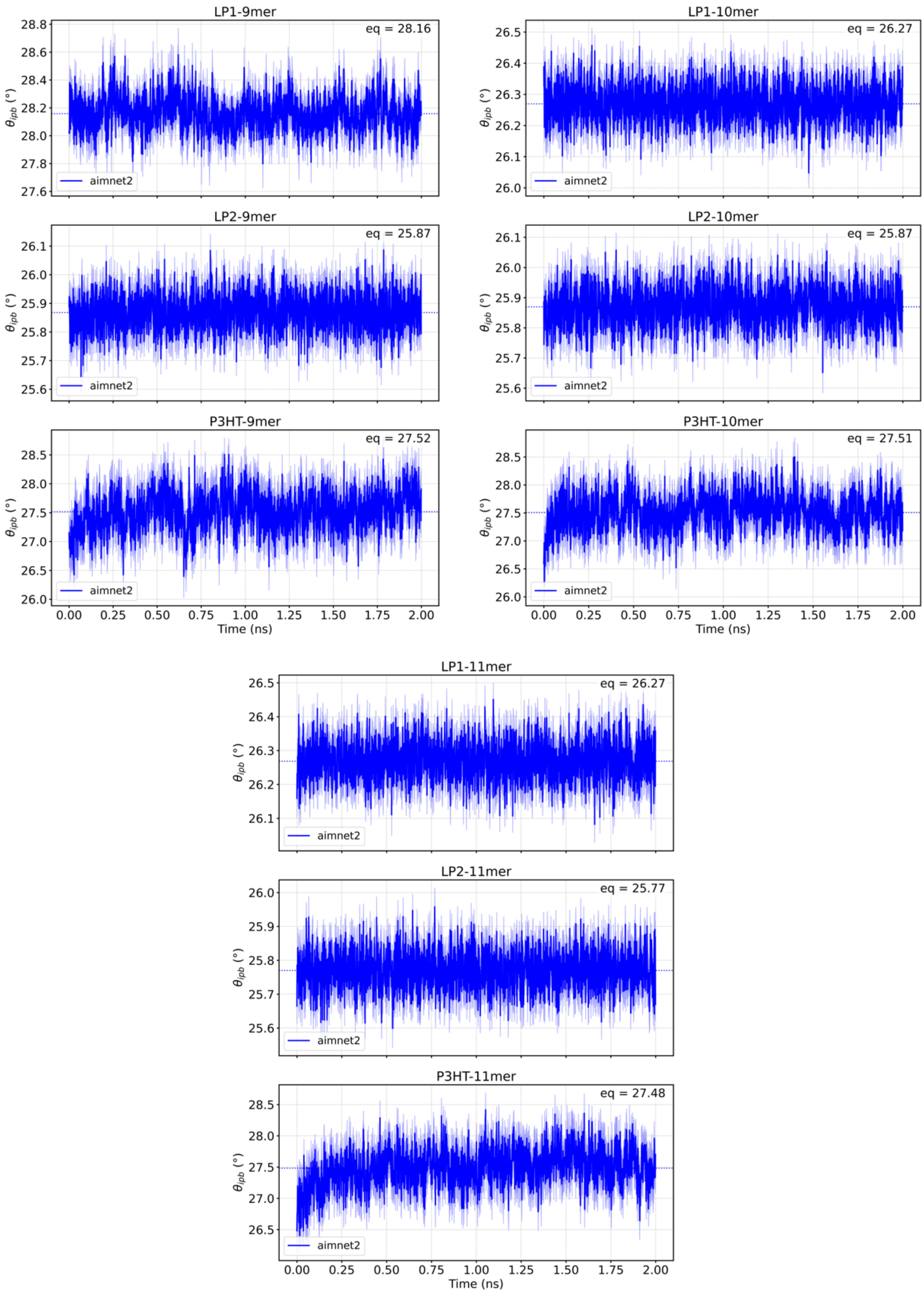

Figure S67. In-plane bending angles of multiple structures used in this study

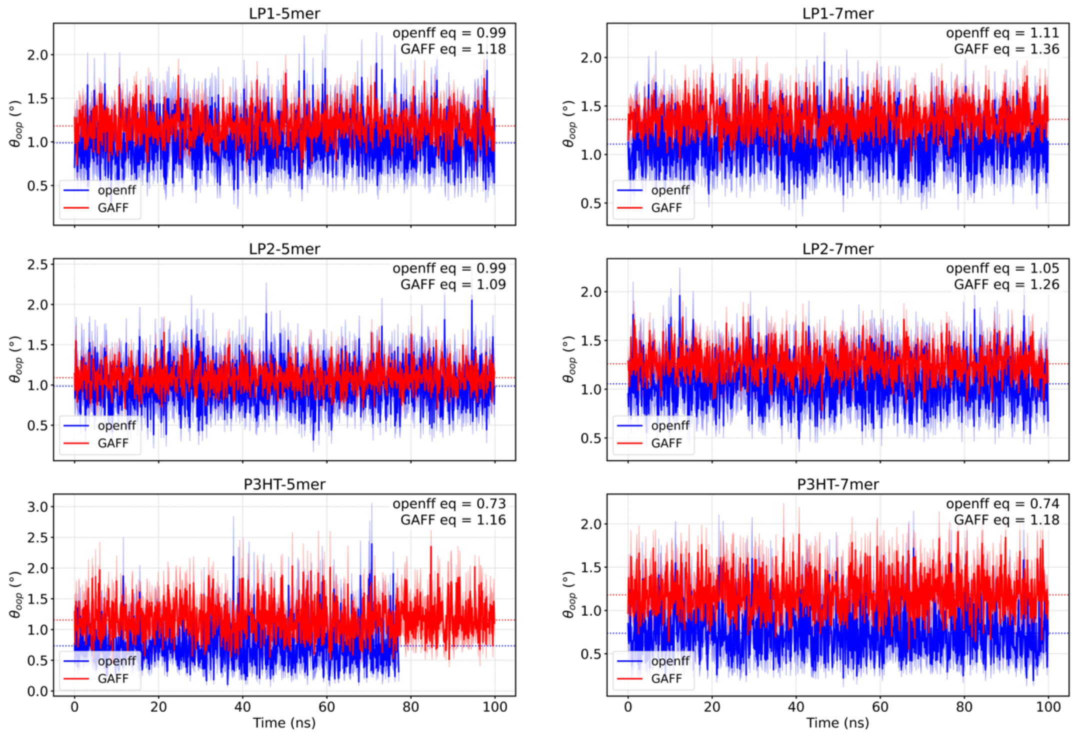
LP1-5mer
openff eq = 0.99
GAFF eq = 1.18
LP1-7mer
openff eq = 1.11
GAFF eq = 1.36
LP2-5mer
openff eq = 0.99
GAFF eq = 1.09
LP2-7mer
openff eq = 1.05
GAFF eq = 1.26
P3HT-5mer
openff eq = 0.73
GAFF eq = 1.16
P3HT-7mer
openff eq = 0.74
GAFF eq = 1.18
openff
GAFF
θ_{oop} (°)
Time (ns)

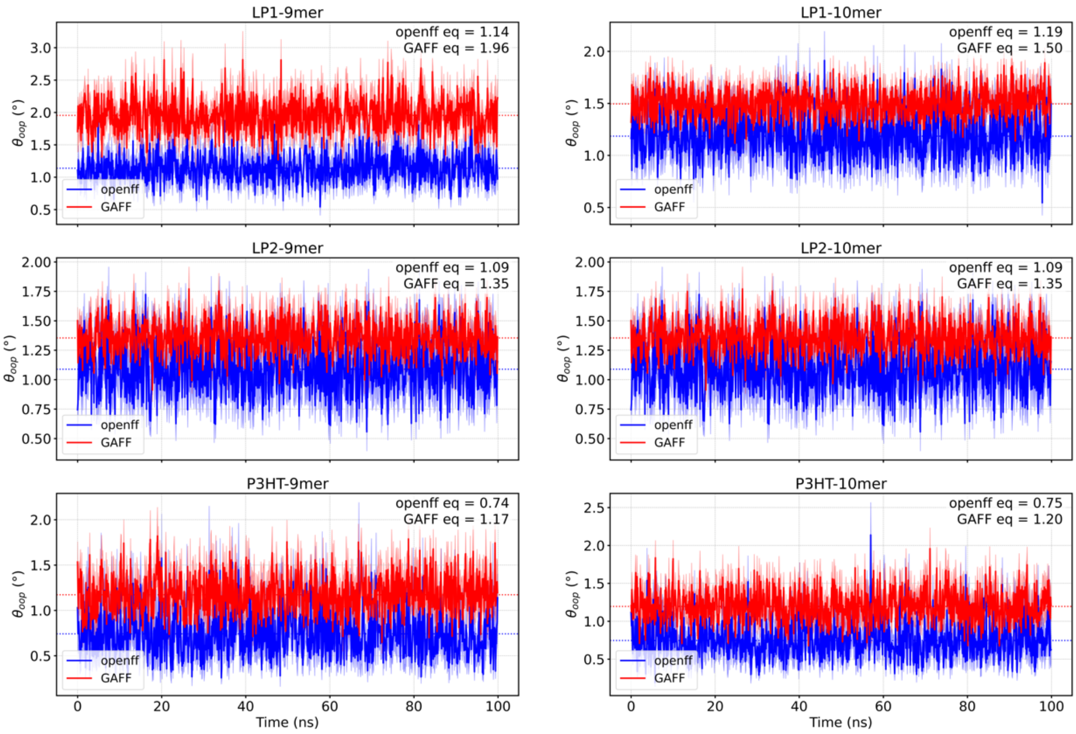
LP1-9mer
openff eq = 1.14
GAFF eq = 1.96
LP1-10mer
openff eq = 1.19
GAFF eq = 1.50
LP2-9mer
openff eq = 1.09
GAFF eq = 1.35
LP2-10mer
openff eq = 1.09
GAFF eq = 1.35
P3HT-9mer
openff eq = 0.74
GAFF eq = 1.17
P3HT-10mer
openff eq = 0.75
GAFF eq = 1.20
openff
GAFF
θ_{oop} (°)
Time (ns)

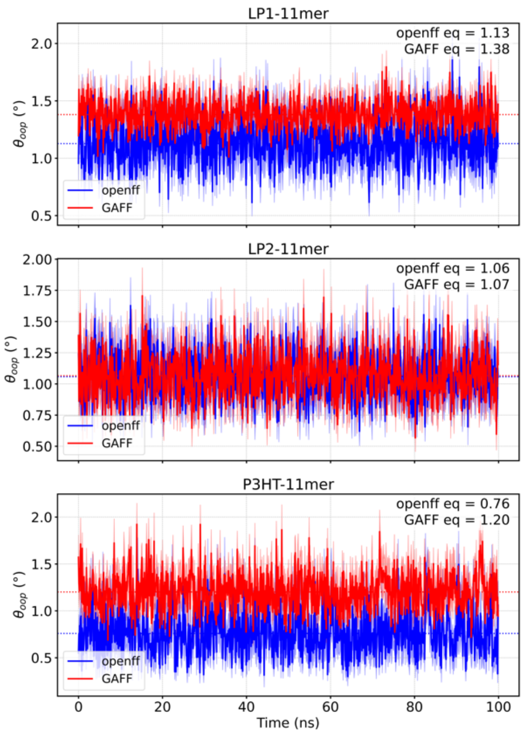
LP1-11mer
openff eq = 1.13
GAFF eq = 1.38
LP2-11mer
openff eq = 1.06
GAFF eq = 1.07
P3HT-11mer
openff eq = 0.76
GAFF eq = 1.20
openff
GAFF
θ_{oop} (°)
Time (ns)

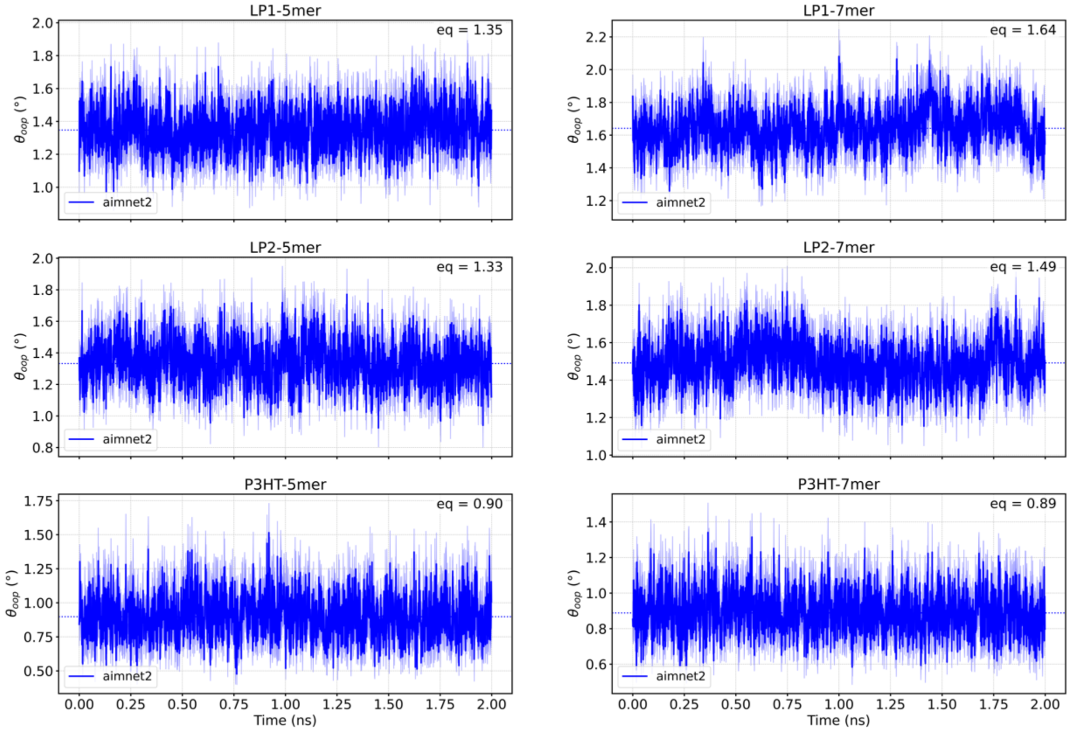
LP1-5mer
eq = 1.35
LP1-7mer
eq = 1.64
LP2-5mer
eq = 1.33
LP2-7mer
eq = 1.49
P3HT-5mer
eq = 0.90
P3HT-7mer
eq = 0.89
aimnet2
θ_{oop} (°)
Time (ns)

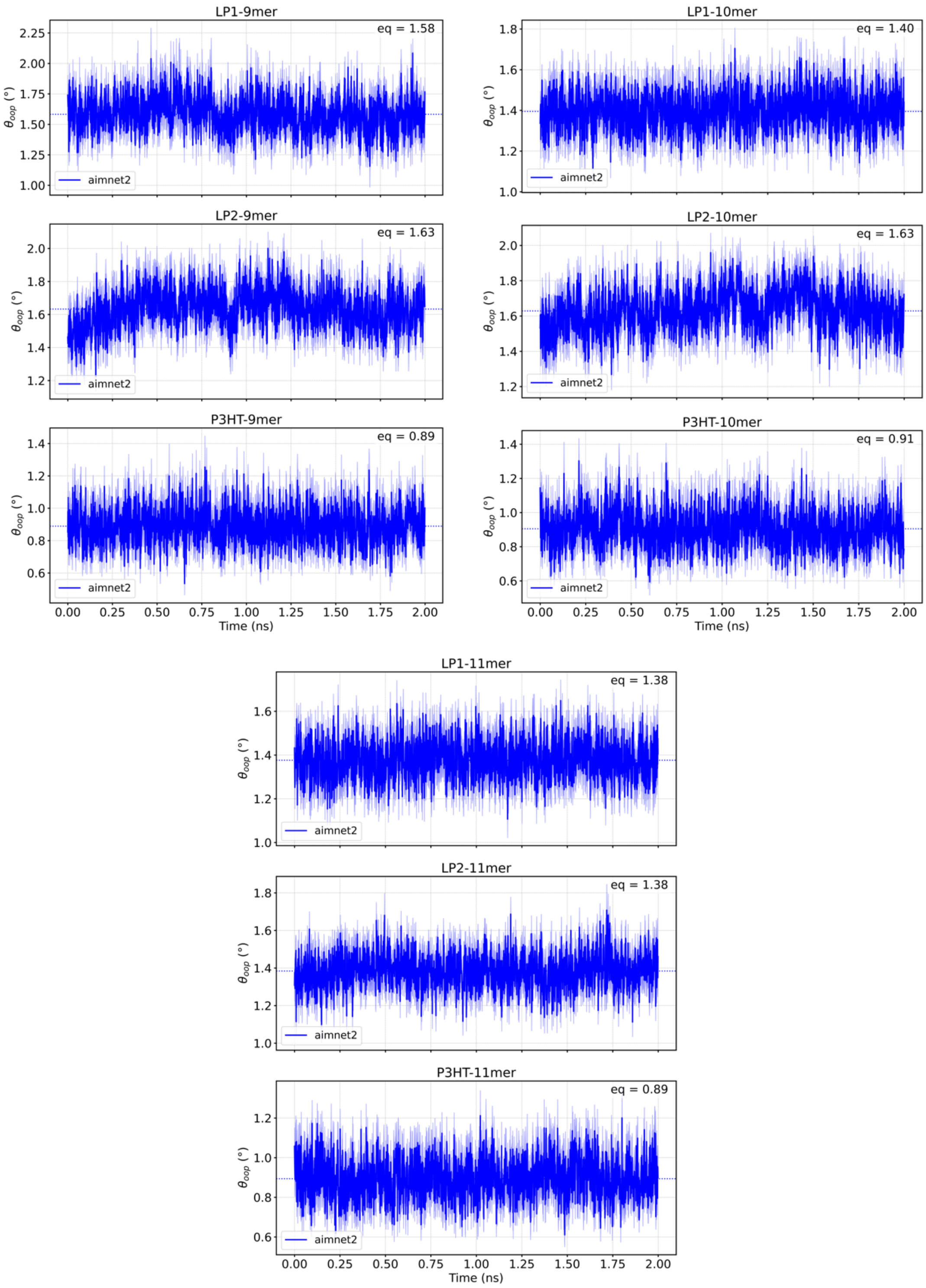

Figure S68. Out-of-plane bending angles of multiple structures used in this study

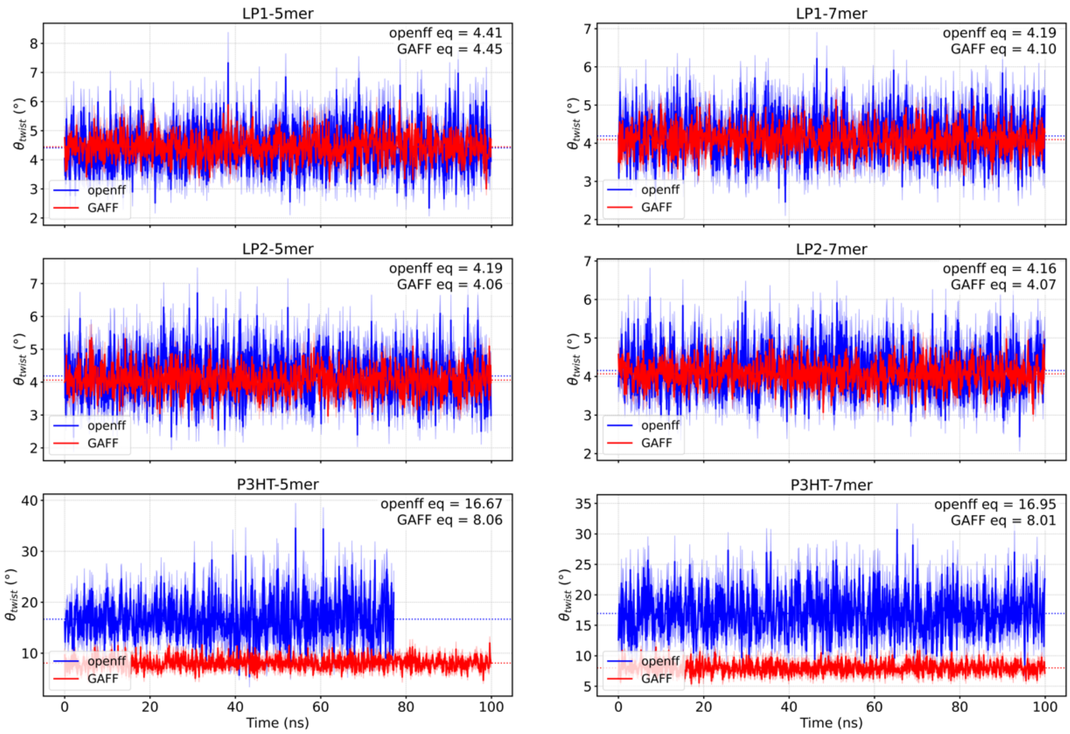
LP1-5mer
openff eq = 4.41
GAFF eq = 4.45
LP1-7mer
openff eq = 4.19
GAFF eq = 4.10
LP2-5mer
openff eq = 4.19
GAFF eq = 4.06
LP2-7mer
openff eq = 4.16
GAFF eq = 4.07
P3HT-5mer
openff eq = 16.67
GAFF eq = 8.06
P3HT-7mer
openff eq = 16.95
GAFF eq = 8.01
openff
GAFF
θ_{twist} (°)
Time (ns)

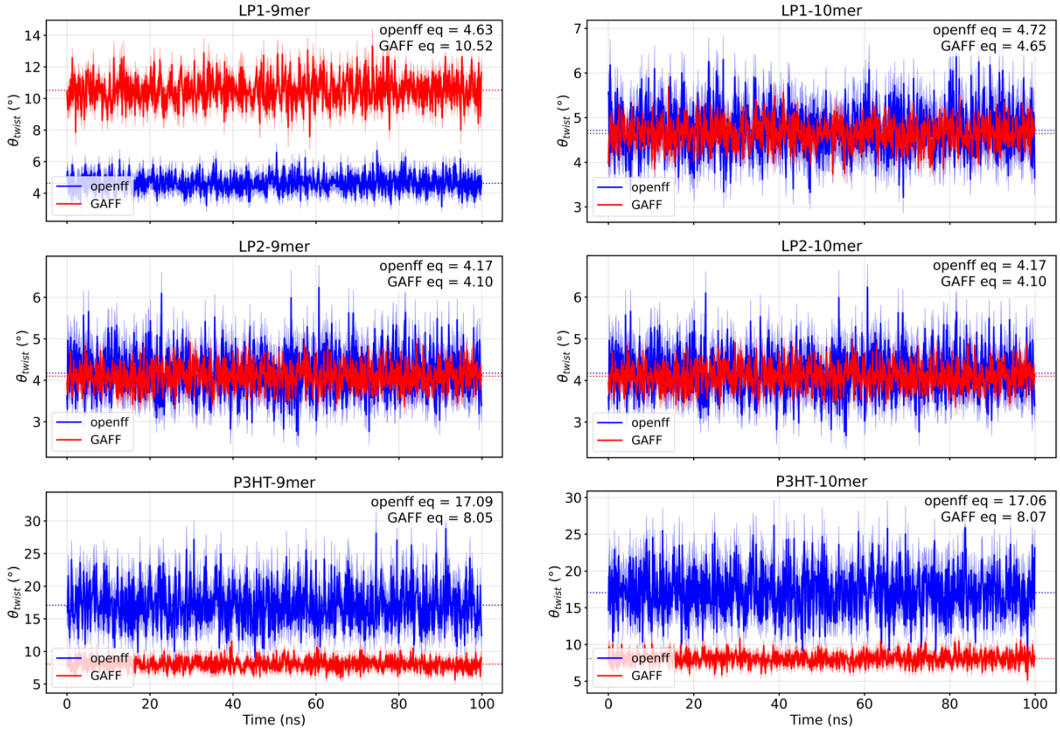
LP1-9mer
openff eq = 4.63
GAFF eq = 10.52
LP1-10mer
openff eq = 4.72
GAFF eq = 4.65
LP2-9mer
openff eq = 4.17
GAFF eq = 4.10
LP2-10mer
openff eq = 4.17
GAFF eq = 4.10
P3HT-9mer
openff eq = 17.09
GAFF eq = 8.05
P3HT-10mer
openff eq = 17.06
GAFF eq = 8.07
openff
GAFF
θ_{twist} (°)
Time (ns)

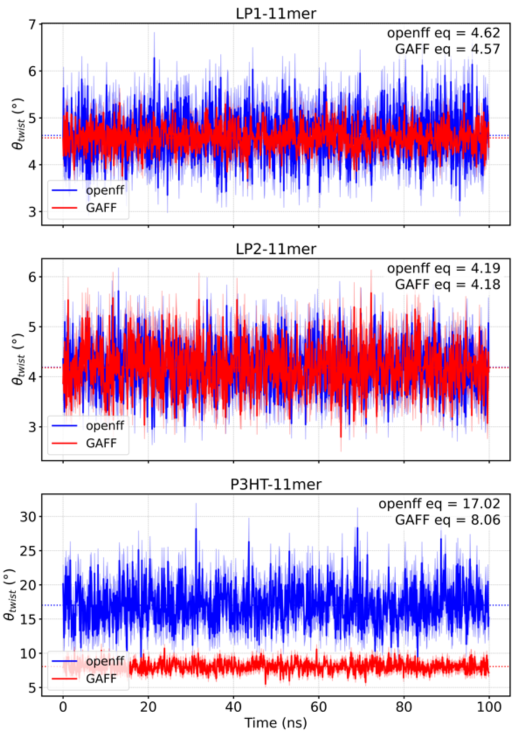
LP1-11mer
openff eq = 4.62
GAFF eq = 4.57
openff
GAFF
LP2-11mer
openff eq = 4.19
GAFF eq = 4.18
P3HT-11mer
openff eq = 17.02
GAFF eq = 8.06
θ_{twist} (°)
Time (ns)

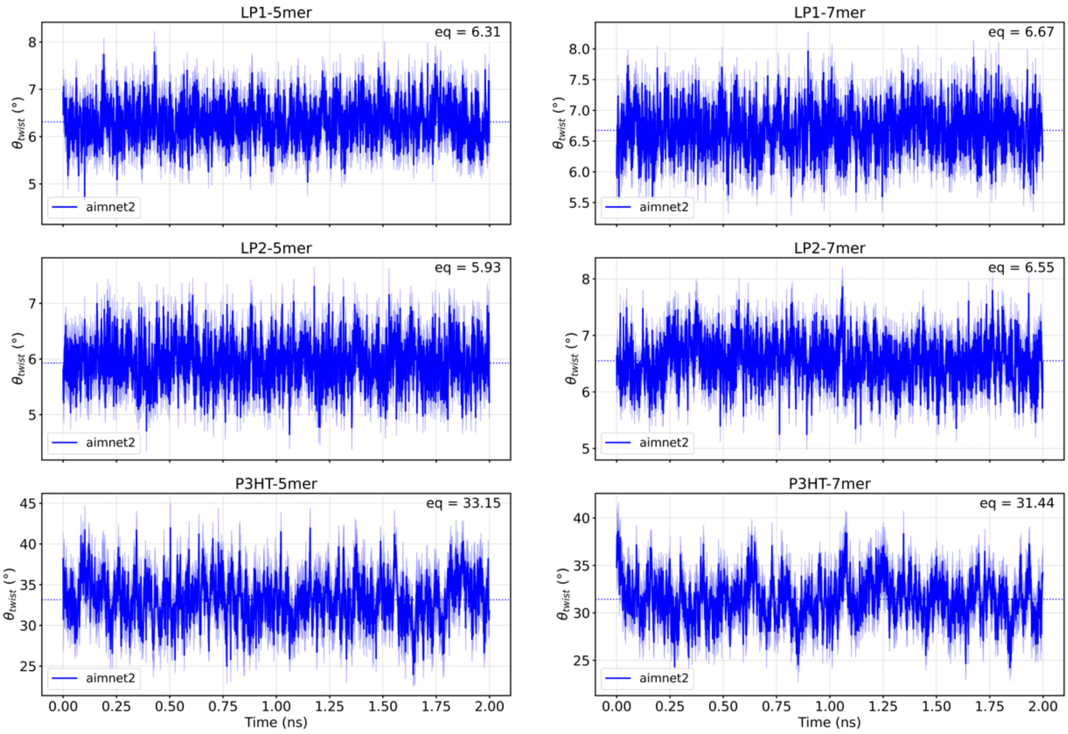
LP1-5mer
eq = 6.31
LP1-7mer
eq = 6.67
LP2-5mer
eq = 5.93
LP2-7mer
eq = 6.55
P3HT-5mer
eq = 33.15
P3HT-7mer
eq = 31.44
aimnet2
θ_{twist} (°)
Time (ns)

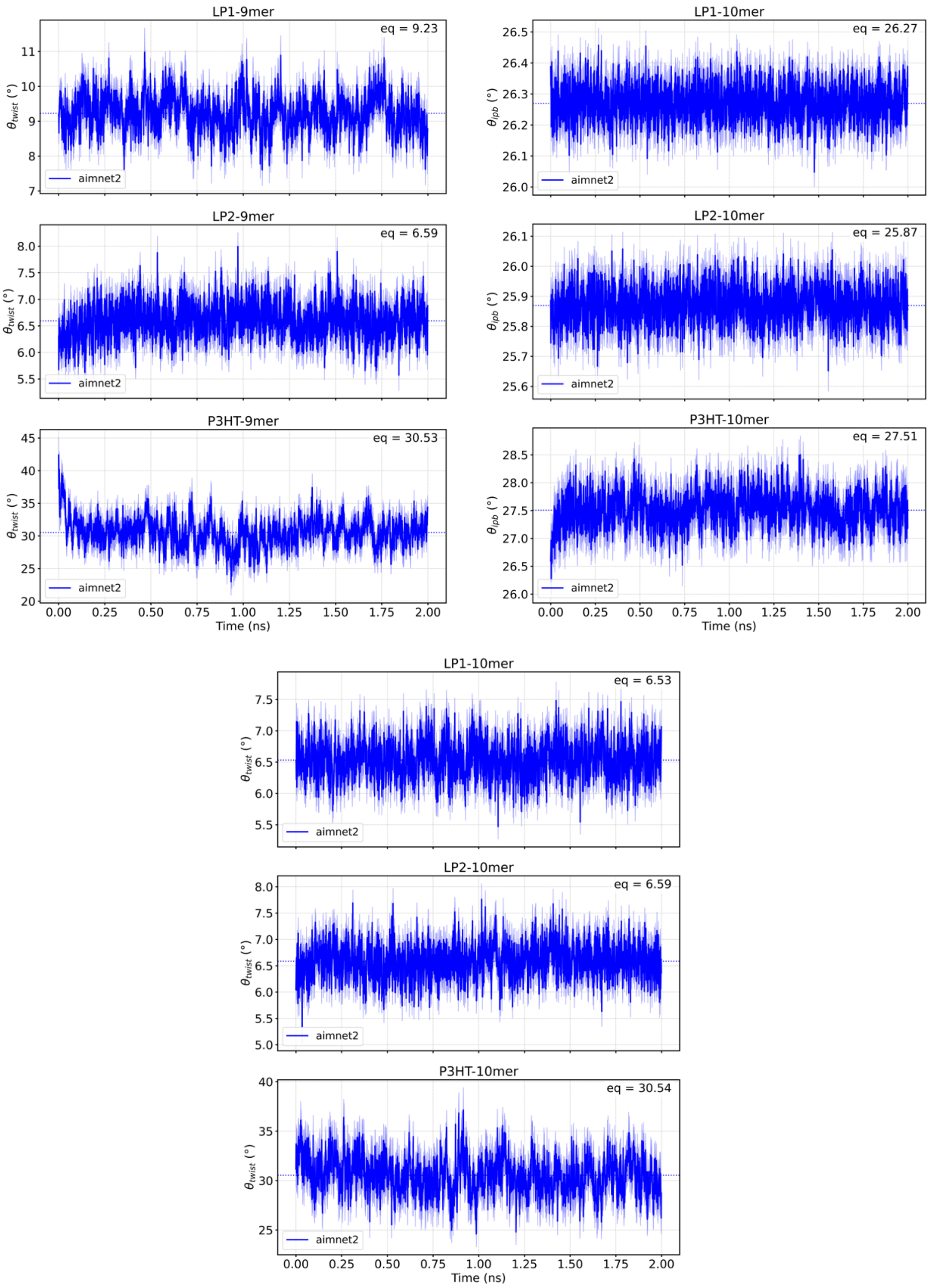

Figure S69. Twist angles of multiple structures used in this study

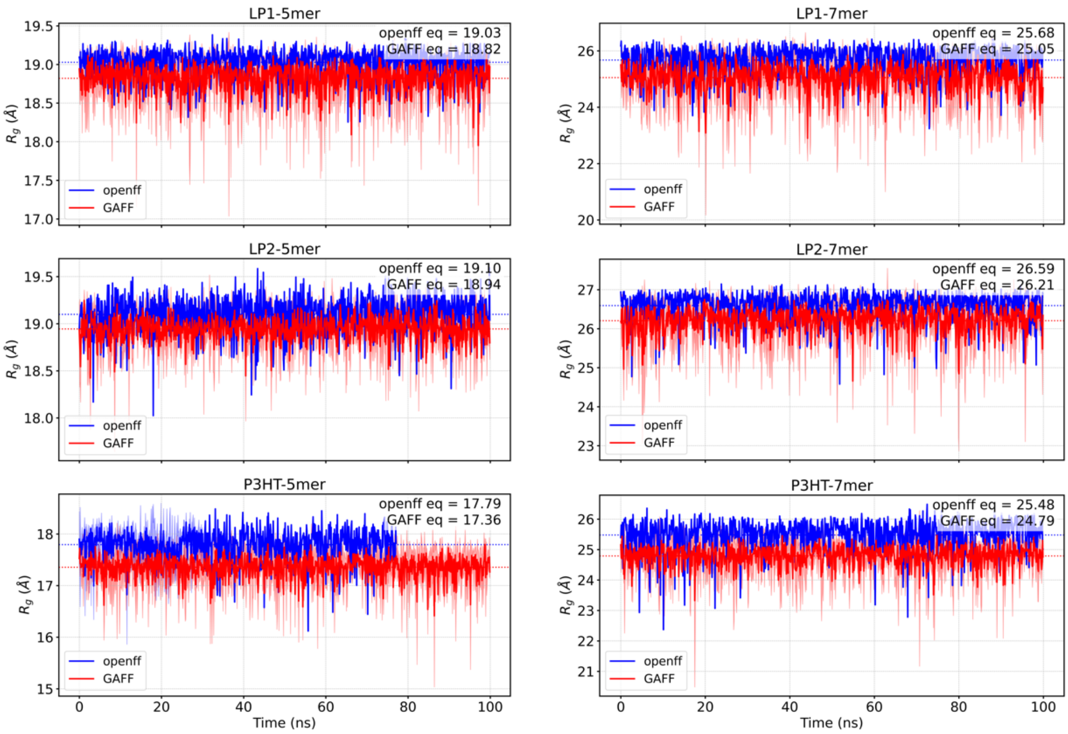

LP1-5mer
openff eq = 19.03
GAFF eq = 18.82
LP1-7mer
openff eq = 25.68
GAFF eq = 25.05
LP2-5mer
openff eq = 19.10
GAFF eq = 18.94
LP2-7mer
openff eq = 26.59
GAFF eq = 26.21
P3HT-5mer
openff eq = 17.79
GAFF eq = 17.36
P3HT-7mer
openff eq = 25.48
GAFF eq = 24.79
R_g (Å)
openff
GAFF
Time (ns)

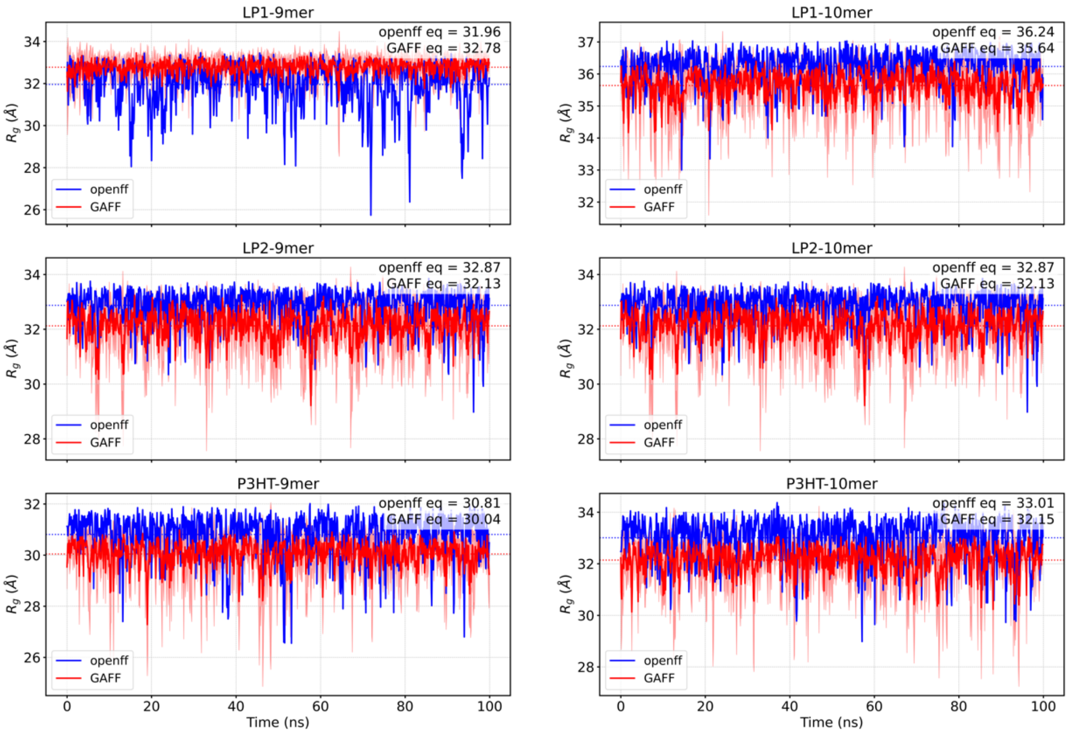

LP1-9mer
openff eq = 31.96
GAFF eq = 32.78
LP1-10mer
openff eq = 36.24
GAFF eq = 35.64
LP2-9mer
openff eq = 32.87
GAFF eq = 32.13
LP2-10mer
openff eq = 32.87
GAFF eq = 32.13
P3HT-9mer
openff eq = 30.81
GAFF eq = 30.04
P3HT-10mer
openff eq = 33.01
GAFF eq = 32.15
R_g (Å)
openff
GAFF
Time (ns)

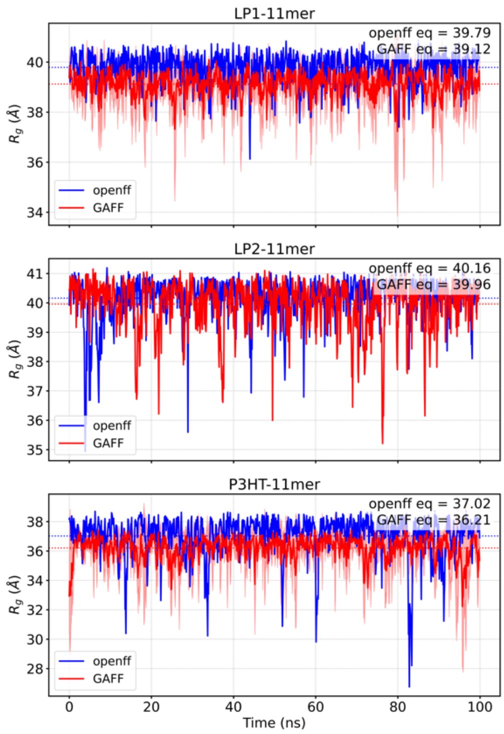
LP1-11mer
openff eq = 39.79
GAFF eq = 39.12
openff
GAFF
LP2-11mer
openff eq = 40.16
GAFF eq = 39.96
P3HT-11mer
openff eq = 37.02
GAFF eq = 36.21
R_g (Å)
Time (ns)

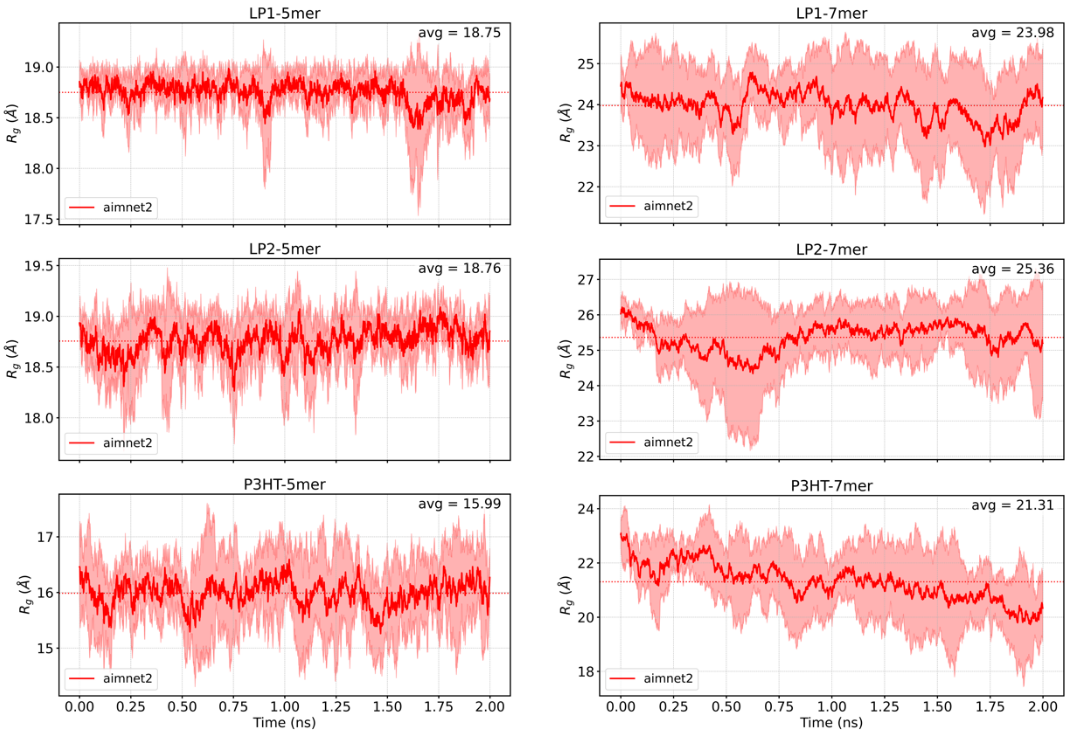
LP1-5mer
avg = 18.75
LP1-7mer
avg = 23.98
LP2-5mer
avg = 18.76
LP2-7mer
avg = 25.36
P3HT-5mer
avg = 15.99
P3HT-7mer
avg = 21.31
aimnet2
R_g (Å)
Time (ns)

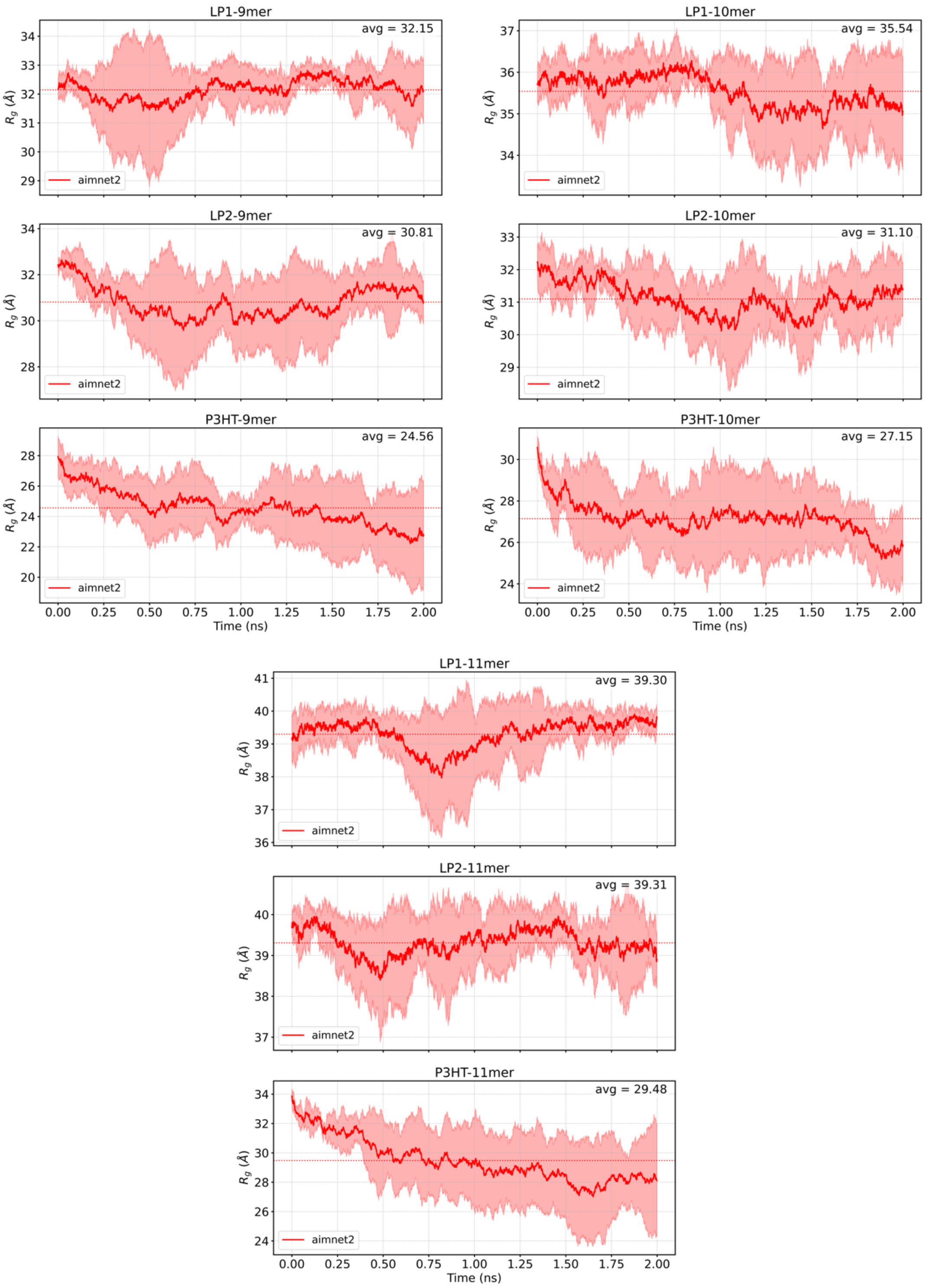

Figure S70. Radius of gyration (R_g) vs time of multiple structures used in this study

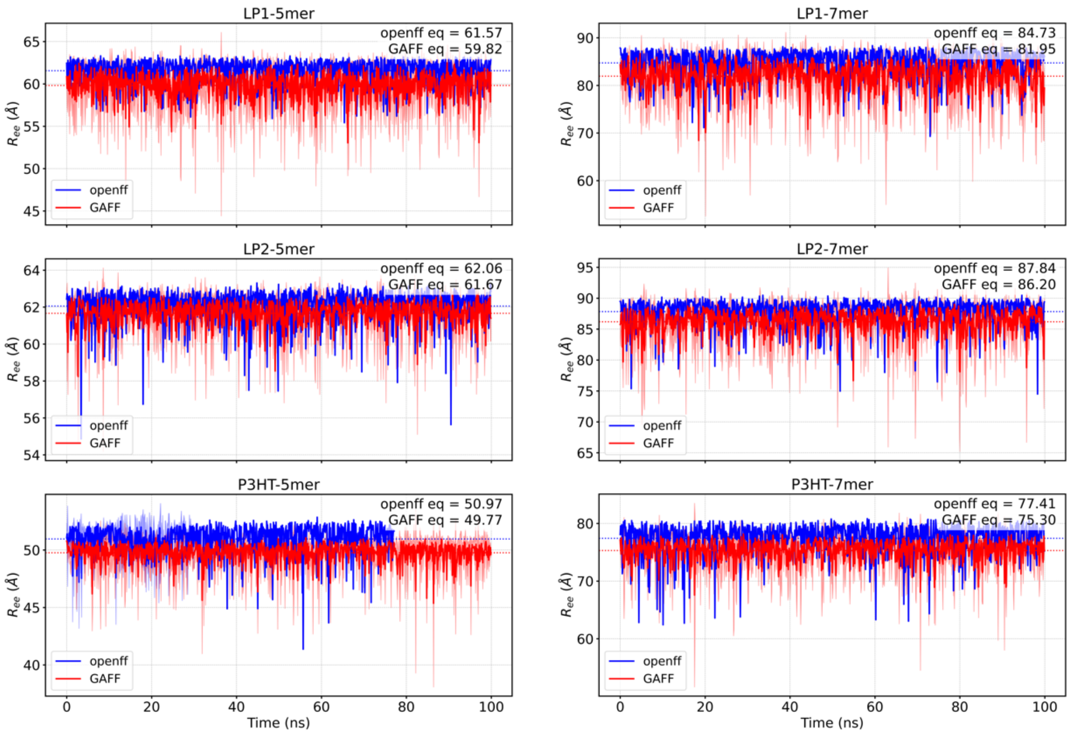
LP1-5mer
openff eq = 61.57
GAFF eq = 59.82
LP1-7mer
openff eq = 84.73
GAFF eq = 81.95
LP2-5mer
openff eq = 62.06
GAFF eq = 61.67
LP2-7mer
openff eq = 87.84
GAFF eq = 86.20
P3HT-5mer
openff eq = 50.97
GAFF eq = 49.77
P3HT-7mer
openff eq = 77.41
GAFF eq = 75.30
openff
GAFF
R_{ee} (Å)
Time (ns)

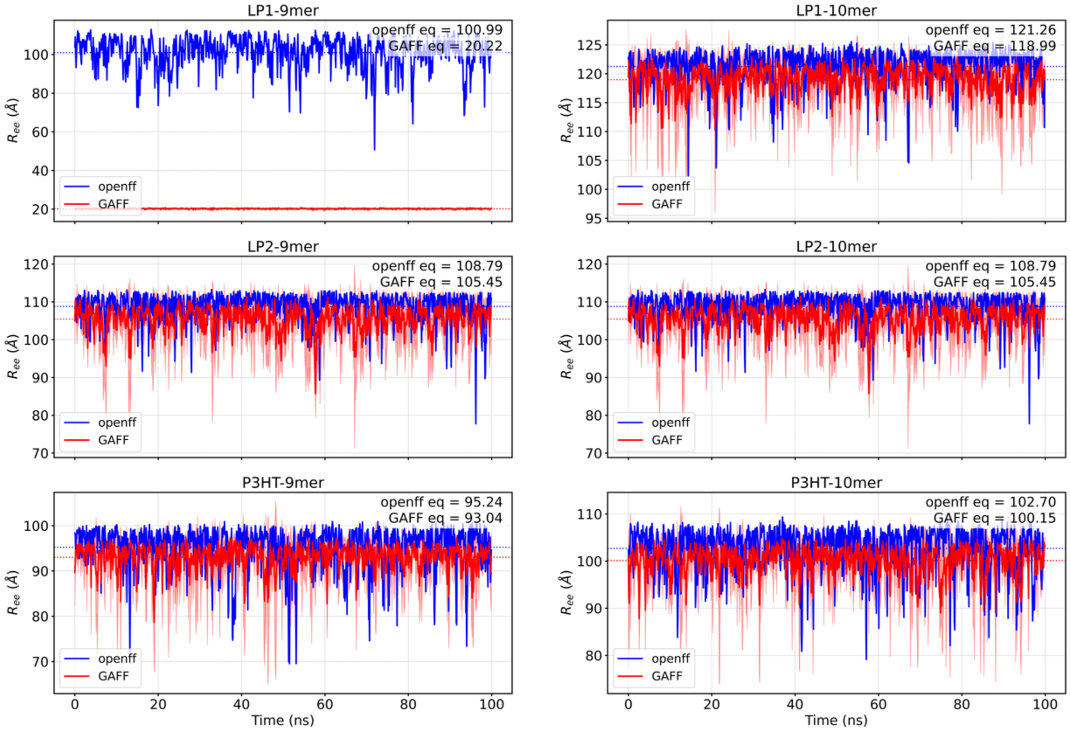
LP1-9mer
openff eq = 100.99
GAFF eq = 20.22
LP1-10mer
openff eq = 121.26
GAFF eq = 118.99
LP2-9mer
openff eq = 108.79
GAFF eq = 105.45
LP2-10mer
openff eq = 108.79
GAFF eq = 105.45
P3HT-9mer
openff eq = 95.24
GAFF eq = 93.04
P3HT-10mer
openff eq = 102.70
GAFF eq = 100.15
openff
GAFF
R_{ee} (Å)
Time (ns)

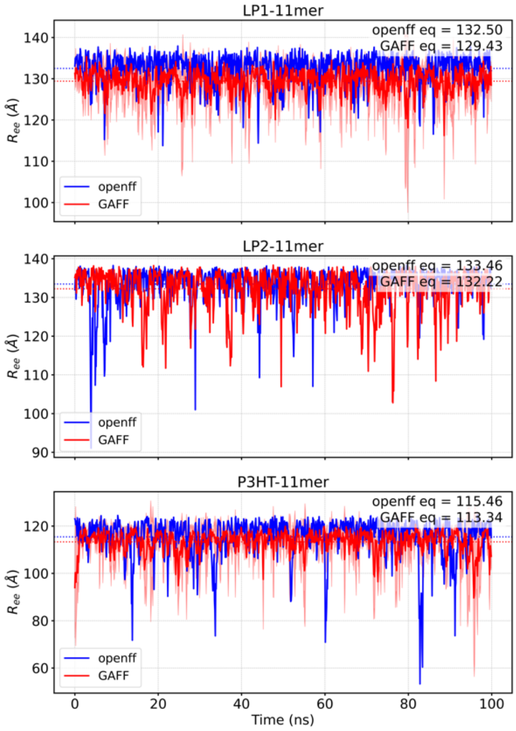
LP1-11mer
openff eq = 132.50
GAFF eq = 129.43
LP2-11mer
openff eq = 133.46
GAFF eq = 132.22
P3HT-11mer
openff eq = 115.46
GAFF eq = 113.34
openff
GAFF
R_{ee} (Å)
Time (ns)

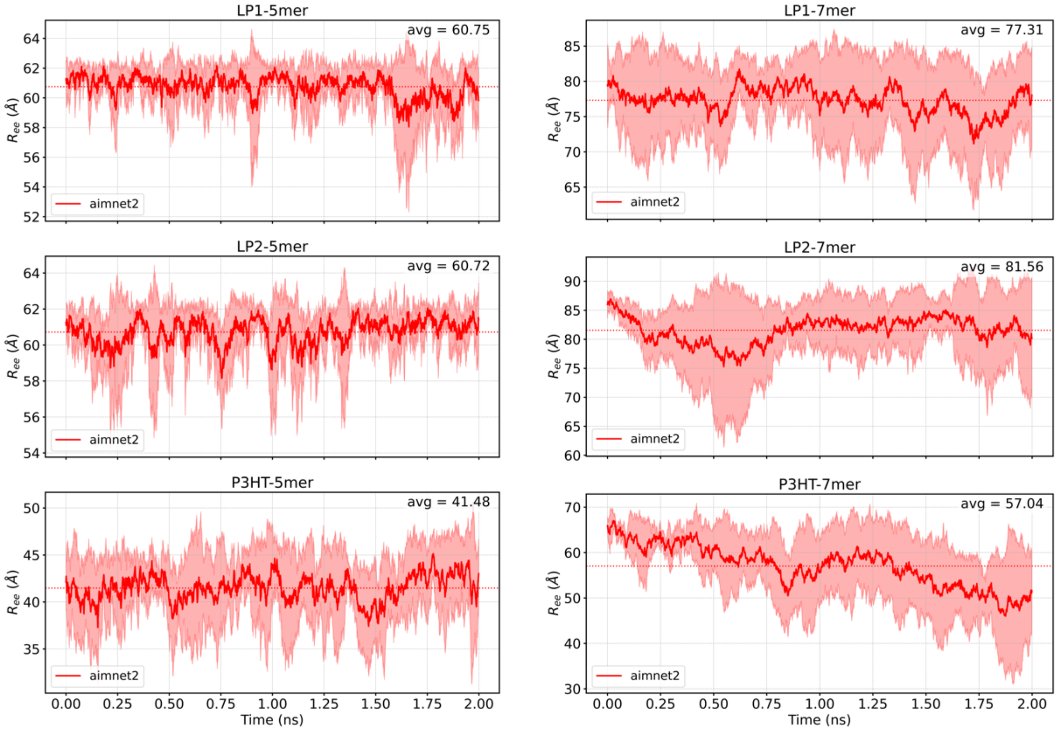
LP1-5mer
avg = 60.75
LP1-7mer
avg = 77.31
LP2-5mer
avg = 60.72
LP2-7mer
avg = 81.56
P3HT-5mer
avg = 41.48
P3HT-7mer
avg = 57.04
aimnet2
R_{ee} (Å)
Time (ns)

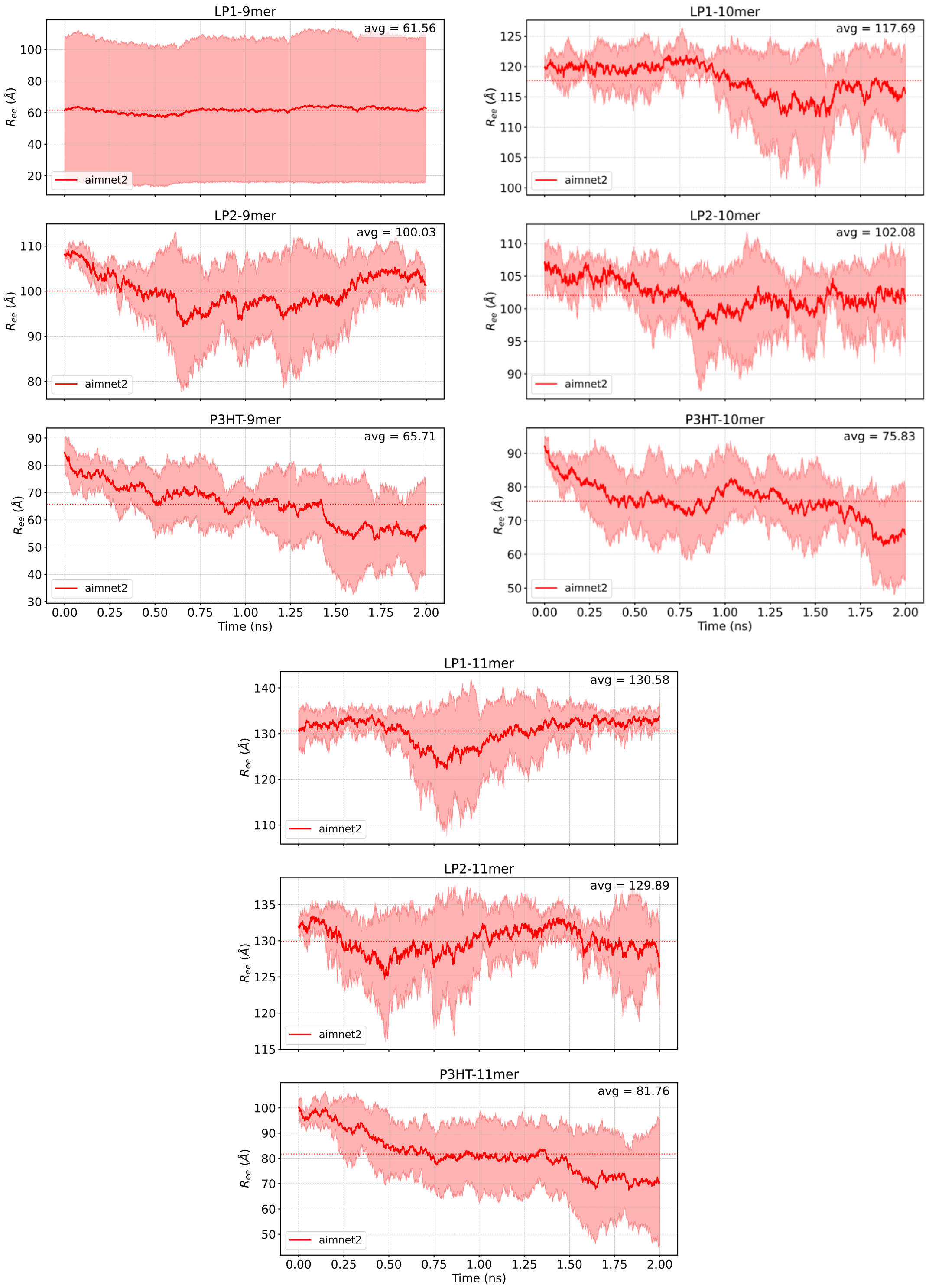

Figure S71. End to end distance (R_{ee}) vs time of multiple structures used in this study

14. References

1. Biswas, K., Chen, Q., Obermann, S., Ma, J., Soler-Polo, D., Melidonie, J., Barragán, A., Sánchez-Grande, A., Lauwaet, K., Gallego, J. M., Miranda, R., Écija, D., Jelínek, P., Feng, X. and Urgel, J. I. On-surface synthesis of non-benzenoid nanographenes embedding azulene and stone-wales topologies. *Angewandte Chemie International Edition* **2024**, *63*, e202318185. https://doi.org/10.1002/anie.202318185
2. Skolia, E. and Kokotos, C. G. Photochemical [2 + 2] cycloaddition of alkenes with maleimides: Highlighting the differences between n-alkyl vs n-aryl maleimides. *ACS Organic & Inorganic Au* **2023**, *3*, 96-103. https://doi.org/10.1021/acsorginorgau.2c00053
3. Vuong, W., Mosquera-Guagua, F., Sanichar, R., McDonald, T. R., Ernst, O. P., Wang, L. and Vederas, J. C. Synthesis of chiral spin-labeled amino acids. *Organic Letters* **2019**, *21*, 10149-10153. https://doi.org/10.1021/acs.orglett.9b04216
4. Chauvin, J.-P. R. and Pratt, D. A. On the reactions of thiols, sulfenic acids, and sulfinic acids with hydrogen peroxide. *Angewandte Chemie International Edition* **2017**, *56*, 6255-6259. https://doi.org/10.1002/anie.201610402
5. Leng, M., Cao, Z., Ma, G., Cao, Y., Hays, M., Gu, X. and Fang, L. Synthesis of isotope-substituted conjugated ladder polymers. *Journal of Polymer Science* **2024**, *62*, 4538-4545. https://doi.org/10.1002/pol.20240418
6. Pedersen, J. S. and Schurtenberger, P. Scattering Functions of Semiflexible Polymers with and without Excluded Volume Effects. *Macromolecules* **1996**, *29*, 7602-7612. https://doi.org/10.1021/ma9607630
7. Cao, Z., Li, Z., Tolba, S. A., Mason, G. T., Xiong, M., Ocheje, M. U., Alesadi, A., Do, C., Hong, K., Lei, T., Rondeau-Gagné, S., Xia, W. and Gu, X. Probing single-chain conformation and its impact on the optoelectronic properties of donor–accepter conjugated polymers. *Journal of Materials Chemistry A* **2023**, *11*, 12928-12940. https://doi.org/10.1039/D2TA09389H
8. Cao, Z., Tolba, S. A., Li, Z., Mason, G. T., Wang, Y., Do, C., Rondeau-Gagné, S., Xia, W. and Gu, X. Molecular Structure and Conformational Design of Donor-Acceptor Conjugated Polymers to Enable Predictable Optoelectronic Property. *Advanced Materials* **2023**, *35*, 2302178. https://doi.org/10.1002/adma.202302178
9. Chai, J.-D. and Head-Gordon, M. Long-range corrected hybrid density functionals with damped atom–atom dispersion corrections. *Physical Chemistry Chemical Physics* **2008**, *10*, 6615-6620. https://doi.org/10.1039/B810189B
10. Weigend, F. and Ahlrichs, R. Balanced basis sets of split valence, triple zeta valence and quadruple zeta valence quality for H to Rn: Design and assessment of accuracy. *Physical Chemistry Chemical Physics* **2005**, *7*, 3297-3305. https://doi.org/10.1039/B508541A
11. Neese, F. Software Update: The ORCA Program System—Version 6.0. *WIREs Computational Molecular Science* **2025**, *15*, e70019. https://doi.org/10.1002/wcms.70019
12. Lu, T. and Chen, F. Multiwfn: A multifunctional wavefunction analyzer. *Journal of Computational Chemistry* **2012**, *33*, 580-592. https://doi.org/10.1002/jcc.22885
13. Chemcraft - graphical software for visualization of quantum chemistry computations. Version 1.8, build 682. https://www.chemcraftprog.com.
14. Wilhelm, J. and Frey, E. Radial distribution function of semiflexible polymers. *Physical Review Letters* **1996**, *77*, 2581-2584. 10.1103/PhysRevLett.77.2581

15. Rubinstein, M. and Colby, R. H. Polymer physics; Oxford University Press, **2003**. DOI: https://doi.org/10.1093/oso/9780198520597.001.0001.
16. Ivanov, M., Gross, J. and Janke, W. Single-chain behavior of poly(3-hexylthiophene). *The European Physical Journal Special Topics* **2017**, *226*, 667-681. https://doi.org/10.1140/epjst/e2016-60348-7
17. O'Boyle, N. M., Banck, M., James, C. A., Morley, C., Vandermeersch, T. and Hutchison, G. R. Open Babel: An open chemical toolbox. *Journal of Cheminformatics* **2011**, *3*, 33. https://doi.org/10.1186/1758-2946-3-33
18. Bannwarth, C., Ehlert, S. and Grimme, S. GFN2-xTB—An accurate and broadly parametrized self-consistent tight-binding quantum chemical method with multipole electrostatics and density-dependent dispersion contributions. *Journal of Chemical Theory and Computation* **2019**, *15*, 1652-1671. https://doi.org/10.1021/acs.jctc.8b01176
19. Pracht, P., Grimme, S., Bannwarth, C., Bohle, F., Ehlert, S., Feldmann, G., Gorges, J., Müller, M., Neudecker, T., Plett, C., Spicher, S., Steinbach, P., Wesołowski, P. A. and Zeller, F. CREST—A program for the exploration of low-energy molecular chemical space. *The Journal of Chemical Physics* **2024**, *160*, 114110. https://doi.org/10.1063/5.0197592
20. Wang, L., McIsaac, A. R., Behara, P. K., Gokey, T., Cavender, C., Horton, J., Westbrook, B. R., Thompson, M. W., Osato, M., Baumann, H. M., Alibay, I., Jang, H., Wagner, J., Cole, D., Bayly, C., & Mobley, D. Openforcefield/openff-forcefields (2024.09.0). *Zenodo* **2024**, https://doi.org/10.5281/zenodo.12797450
21. Wang, J., Wang, W., Kollman, P. A. and Case, D. A. Automatic atom type and bond type perception in molecular mechanical calculations. *Journal of Molecular Graphics and Modelling* **2006**, *25*, 247-260. https://doi.org/10.1016/j.jmgm.2005.12.005
22. Mobley, D. L., Bannan, C. C., Rizzi, A., Bayly, C. I., Chodera, J. D., Lim, V. T., Lim, N. M., Beauchamp, K. A., Slochower, D. R., Shirts, M. R., Gilson, M. K. and Eastman, P. K. Escaping atom types in force fields using direct chemical perception. *Journal of Chemical Theory and Computation* **2018**, *14*, 6076-6092. https://doi.org/10.1021/acs.jctc.8b00640
23. Woods, R. J. and Chappelle, R. Restrained electrostatic potential atomic partial charges for condensed-phase simulations of carbohydrates. *Journal of Molecular Structure: THEOCHEM* **2000**, *527*, 149-156. https://doi.org/10.1016/S0166-1280(00)00487-5
24. Neese, F. The ORCA program system. *WIREs Computational Molecular Science* **2012**, *2*, 73-78. https://doi.org/10.1002/wcms.81
25. Eastman, P., Galvelis, R., Peláez, R. P., Abreu, C. R. A., Farr, S. E., Gallicchio, E., Gorenko, A., Henry, M. M., Hu, F., Huang, J., Krämer, A., Michel, J., Mitchell, J. A., Pande, V. S., Rodrigues, J. P., Rodriguez-Guerra, J., Simmonett, A. C., Singh, S., Swails, J., Turner, P., Wang, Y., Zhang, I., Chodera, J. D., De Fabritiis, G. and Markland, T. E. OpenMM 8: Molecular dynamics simulation with machine learning potentials. *The Journal of Physical Chemistry B* **2024**, *128*, 109-116. https://doi.org/10.1021/acs.jpcb.3c06662
26. Anstine, D. M. Z., R.; Isayev, O. AIMNet2: A neural network potential to meet your neutral, charged, organic, and elemental-organic needs. *Chemical Science* **2025**, *16*, 10228-10244. https://doi.org/10.1039/D4SC08572H
27. Hjorth Larsen, A., Jørgen Mortensen, J., Blomqvist, J., Castelli, I. E., Christensen, R., Dułak, M., Friis, J., Groves, M. N., Hammer, B., Hargus, C., Hermes, E. D., Jennings, P. C., Bjerre Jensen, P., Kermode, J., Kitchin, J. R., Leonhard Kolsbjerg, E.,

Kubal, J., Kaasbjerg, K., Lysgaard, S., Bergmann Maronsson, J., Maxson, T., Olsen, T., Pastewka, L., Peterson, A., Rostgaard, C., Schiøtz, J., Schütt, O., Strange, M., Thygesen, K. S., Vegge, T., Vilhelmsen, L., Walter, M., Zeng, Z. and Jacobsen, K. W. The atomic simulation environment—a Python library for working with atoms. *Journal of Physics: Condensed Matter* **2017**, *29*, 273002. https://doi.org/10.1088/1361-648X/aa680e
28. Michaud-Agrawal, N., Denning, E. J., Woolf, T. B. and Beckstein, O. MDAnalysis: A toolkit for the analysis of molecular dynamics simulations. *Journal of Computational Chemistry* **2011**, *32*, 2319-2327. https://doi.org/10.1002/jcc.21787
29. Gowers, R. J., Linke, M., Barnoud, J., Reddy, T. J. E., Melo, M. N., Seyler, S. L., Domanski, J., Dotson, D. L., Buchoux, S., Kenney, I. M. and Beckstein, O. MDAnalysis: A python package for the rapid analysis of molecular dynamics simulations. United States, **2019**.
30. Chemcraft—Graphical software for visualization of quantum chemistry computations. *Version 1.8*, Build 682. https://www.chemcraftprog.com
